# Immunogenicity and safety of self-amplifying mRNA COVID-19 vaccine (ARCT-2303), with or without co-administration of seasonal inactivated influenza vaccine in adults: a phase 3, randomised, controlled, observer-blind, multicentre study

**DOI:** 10.1016/j.eclinm.2025.103428

**Published:** 2025-08-20

**Authors:** Michelle L. Giles, Charissa Tabora, Carmen Baccarini, Leonela Barrientos, Javier Cespedes Vargas, May Emmeline Montellano, Paul Nguyen, Sachin Deshmukh, Munro Neville, Matthew Hohenboken, Josephine van Boxmeer, Hongfan Jin, Roberto Bugarini, Xuexuan Liu, Judd L. Walson, Carole Verhoeven, Igor Smolenov

**Affiliations:** a Peter Doherty Institute for Immunity and Infection, The University of Melbourne, Australia; b Tropical Disease Foundation Inc., Makati, Metro Manila, Philippines; c Arcturus Therapeutics, San Diego, USA; d Inversiones en Investigacion Medica S.a, Tegucigalpa, Honduras; e Clinica San Agustin Mostt S.a, San Jose, Costa Rica; f Far Eastern University Nicanor Reyes Medical Foundation, Quezon City, Philippines; g Emeritus Research Melbourne, Camberwell, Australia; h Griffith University Clinical Trials Unit, Gold Coast Campus, Australia; i Momentum Clinical Research, St Leonards, Australia; j CSL, Waltham, MA, USA; k Walson Consulting, Seattle, WA, USA

**Keywords:** Self-amplifying mRNA, COVID-19 vaccine, Co-administration, Immunogenicity, Safety

## Abstract

**Background:**

A recently licenced self-amplifying mRNA (sa-mRNA) COVID-19 vaccine induces a robust, broad, and long-lasting immune response, extending the arsenal of efficacious COVID-19 countermeasures. We ran a clinical study to assess the benefits of vaccine strain update and the feasibility of co-administration with influenza vaccines.

**Methods:**

Between March 27, 2024 and April 10, 2025, we performed a randomised, observer-blind, placebo-controlled, phase 3 study with 1499 adult participants to compare immune responses of sa-mRNA vaccine, encoding spike glycoprotein of the XBB.1.5 subvariant (ARCT-2303), with vaccine encoding the ancestral strain (ARCT-154), as measured by geometric mean titres of neutralising antibodies and SARS-CoV-2 neutralising antibody seroconversion rates against the Omicron XBB.1.5, and to assess the immunological non-inferiority of co-administered ARCT-2303 and influenza vaccines compared with separately administered vaccines, as measured by neutralising antibodies against Omicron XBB.1.5.6 and haemagglutinin inhibition against influenza vaccine strains. Reactogenicity (adverse events on Days 1–7) and safety (adverse events on Days 1–181) were also assessed. The trial was registered on ClinicalTrials.gov (identifier NCT06279871).

**Findings:**

The geometric mean ratio (ARCT-2303/ARCT-154) of neutralising antibodies against Omicron XBB.1.5.6 on Day 29 was 2.7 (95% confidence interval (CI): 2.3–3.2), and the seroconversion rate difference was 28.4% (21.8–34.9); both met the prespecified superiority criteria. Concomitant administration of ARCT-2303 had no impact on the immune response to the quadrivalent influenza vaccine antigens, whether the non-adjuvanted vaccine given to 18‒64 year-old adults or the adjuvanted vaccine given to adults 65 years and older. The non-inferiority of the immune response against Omicron XBB.1.5.6 was also demonstrated when ARCT-2303 was co-administered or administered separately.

**Interpretation:**

We conclude that ARCT-2303 induces a robust immune response against the vaccine variant of SARS-CoV-2 and can be co-administered with licenced influenza vaccines in adults with no impact on the safety or immunogenicity of either vaccine.

**Funding:**

The study is funded by 10.13039/100008322CSL under a collaboration agreement with Arcturus Therapeutics.

**Research in context**

**Evidence before this study**

Efficacy and safety of self-amplifying mRNA (sa-mRNA) COVID-19 vaccine were recently demonstrated, leading to the licensure of the first sa-mRNA COVID-19 vaccine (ARCT-154) in Japan in 2023. The administration of sa-mRNA COVID-19 vaccine was associated with a higher neutralising immune response and longer antibody persistence than conventional mRNA vaccines. A PubMed search covering the past 10 years, with no language restrictions, using the terms “self-amplifying RNA” and “self-replicating RNA” was conducted. This search identified four phase 1 clinical studies of sa-mRNA vaccines other than ARCT-154 in humans. Of these studies, one study focused on the treatment of metastatic solid tumours, another was a dose-ranging study of an experimental sa-mRNA vaccine against rabies, and two others investigated an sa-mRNA COVID-19 vaccine, administered as a booster dose. All four trials demonstrated the potential of sa-mRNA vaccines, with robust humoural or cell-mediated responses and generally favourable safety profiles. The results for sa-mRNA COVID-19 vaccines were limited to vaccines targeting the ancestral strain of SARS-CoV-2. Moreover, no data is available to support concomitant administration of sa-mRNA with other licenced vaccines.

**Added value of this study**

This study represents the first use of an sa-mRNA COVID-19 vaccine, encoding a recommended variant of SARS-CoV-2, as well as the first study evaluating concomitant administration study of sa-mRNA with other licenced vaccines. We show that strain replacement is associated with anticipated immunological benefits compared to the ancestral-strain-containing vaccine, and the sa-mRNA COVID-19 vaccine can be co-administered with licenced influenza vaccines without impacting the safety or immunogenicity of either vaccine.

**Implications of all the available evidence**

This study demonstrates that sa-mRNA COVID-19 vaccine provides consistent immunogenicity, safety, and tolerability when updated for new variants. It also shows that co-administration with licenced influenza vaccines does not affect either vaccine's safety or immune response. These findings support integrated vaccination strategies and offer practical guidance for healthcare providers aiming to optimise seasonal immunisation efforts.

## Introduction

Following the COVID-19 pandemic, mRNA vaccines have become established in the vaccination calendar to counter the ongoing emergence of novel SARS-CoV-2 variants. In most countries, COVID-19 vaccines and influenza vaccines may be administered at the same health visit, so safety and immunogenicity data on co-administration of these vaccines is of public health interest. Several recent studies have assessed the co-administration of conventional mRNA or recombinant protein COVID-19 vaccines with inactivated influenza vaccines,^1^, ^2^, ^3^ and companies have reported the development of combination COVID-19 and influenza vaccines using mRNA^4^ and recombinant protein.^5^

Recently, a self-amplifying mRNA (sa-mRNA) vaccine (Kostaive, Arcturus Therapeutics Inc., San Diego, CA, USA) has been licenced in Japan and the European Union.^6^ sa-mRNA vaccines include an mRNA replicase component to increase intracellular levels of expressed mRNA to in turn elicit sustained levels of target antigenic-protein. These, combined with enhanced innate immune responses, lead to potent and long-lasting antigen-specific humoural and cellular immunity and clinical protection.^7^^,^^8^ In clinical trials, Kostaive displayed a superior immune response in terms of the magnitude and durability of neutralising antibody responses with a 1.3–1.4-fold difference in antibody titres vs conventional mRNA at 4 weeks post-booster and a 1.7–2.4-fold difference at later time points up to 1-year post-vaccination.^9^, ^10^, ^11^ Kostaive also induced a higher immune response after primary immunisation series compared to adenovirus-vector vaccine with 1.6–3.7-fold intergroup differences at different time points up to 1 year post-vaccination and relative vaccine efficacy against symptomatic COVID-19 disease of 19.8%.^12^ A formulation containing sa-mRNA coding for S-protein from the D614G and Omicron BA.4/5 variants also demonstrated higher neutralising antibody responses with greater breadth against different COVID-19 strains compared with a bivalent conventional mRNA vaccine.^13^

In addition to primary vaccination and booster studies with the ancestral strain-encoding vaccine, the United States (US) Food and Drug Administration (FDA) requested the conduct of an additional pivotal clinical study with the updated variant vaccine, ARCT-2303, which targets the Omicron XBB.1.5 sub-variant, to confirm the value of strain replacement in the new vaccine in comparison with the original vaccine.^14^ This study was also designed to assess the feasibility of concomitant administration of ARCT-2303 with routinely recommended, age-appropriate influenza vaccines in young and older adult populations. Here we report the primary results of this study conducted in four different countries in adults previously vaccinated with COVID-19 vaccines. Participants were young adults (18–64 years) and older adults (65 years and older) who initially received one dose of ARCT-2303 and one dose of age-recommended quadrivalent influenza vaccine (QIV for young adults and adjuvanted QIV [aQIV] for older adults) either concomitantly or separately, with saline placebo used to maintain the blind.

## Methods

### Study design

This randomised, multi-centre, observer-blind, placebo-controlled, phase 3 study was conducted at 29 medical centres in Australia (22 sites), Costa Rica (2 sites), Honduras (3 sites), and the Philippines (2 sites) between March 27, 2024 and April 10, 2025. The trial was registered on ClinicalTrials.gov (identifier NCT06279871).

### Outcomes

Primary objectives were i) the demonstration of superior immunogenicity of a booster dose of ARCT-2303 vaccine, encoding the XBB.1.5 SARS-CoV-2 subvariant as recommended by the World Health Organization (WHO) and the US Centers for Disease Control and Prevention (CDC) for the 2023/24 season, compared with a booster dose of ARCT-154 vaccine encoding the ancestral SARS-CoV-2 strain (historical cohort), when measured as geometric mean titres (GMTs) of neutralising antibodies against Omicron XBB.1.5.6 sub-lineage, and ii) noninferior immunogenicity in terms of neutralising antibody seroconversion rate (SCR) against Omicron XBB.1.5.6 sub-lineage. Two other primary objectives were to show iii) non-inferiority of the immune responses as measured by GMTs of haemagglutinin inhibition (HAI) titres against influenza viruses included in the quadrivalent influenza vaccine and iv) non-inferiority of the immune responses of ARCT-2303, as measured by GMTs of neutralising antibodies against Omicron XBB.1.5.6 sub-lineage, when both vaccines were administered concomitantly and separately. Primary objectives iii and iv were assessed in individuals 18–64 years of age. Secondary objectives included assessments of the immunogenicity of all tested vaccines in older adults (≥65 years), the persistence of neutralising antibodies against Omicron XBB.1.5.6 sub-lineage, and the safety and reactogenicity of all study vaccines. Cross-neutralising responses against other SARS-CoV-2 variants were assessed as an exploratory objective. The full list of all study outcomes is shown in Supplementary Table S1, p.3.

### Participants

The study was conducted according to Good Clinical Practice guidelines, the Declaration of Helsinki, applicable regulatory requirements, and local regulations. Protocols and consent forms were approved by Independent Ethics Committees at each study site. All study participants provided written informed consent before any study-specific procedures.

Eligible participants were healthy adults (18–64 and 65 years and above) who had previously received at least three doses of mRNA COVID-19 vaccine, with the last dose received at least five months before recruitment into this study. The historical cohort for assessment of primary objectives i and ii consisted of adult participants in a published phase 3 trial (ARCT-154-J01) in Japan conducted to assess the immunogenicity and safety of the self-amplifying mRNA vaccine, ARCT-154, compared with an approved conventional mRNA vaccine.^9^ Any participant with a history of symptomatic COVID-19 disease within the previous five months or previous vaccination with an Omicron XBB1.5-containing vaccine was excluded; a SARS-CoV-2 rapid antigen test was performed at the time of enrolment, and positive persons were excluded. Female participants of childbearing potential were required to practice agreed contraceptive methods during the trial and were excluded if a pregnancy test prior to enrolment was positive. Other inclusion/exclusion criteria are shown in Supplementary, p.4.

### Randomisation and masking

After providing informed consent, eligible volunteers in each age cohort (18–64 years of age and ≥65 years of age) were enrolled and randomised 1:1:1 with a block size of 12, using an interactive response system (IRT) to one of the three study groups (ARCT-2301 + (a)QIV, ARCT-2303 + Placebo, and Placebo + (a)QIV). Randomisation was stratified by COVID-19 vaccination history (number and type of previous vaccinations). The computer-generated randomisation list was prepared by the contract research organization (CRO) Novotech.

An unblinded dosing team, not involved with study participant's evaluation, prepared and administered the study vaccines. Study vaccines syringes were masked to avoid unblinding of the participant. Participants and all other study personnel, including laboratory staff, the CRO, and the sponsor's representatives, remained blinded to allocation throughout the study.

### Procedures

At the first study visit, data on medical history and current health status were collected and the first vaccine/placebo doses were prepared and administered by a dosing team who were unblinded to the study allocation. Participants were monitored on site for 30 min post vaccination for immediate reactions and then completed electronic diaries to report solicited local reactions—injection site pain, erythema, and swelling—and systemic adverse events (AEs)—fatigue, headache, myalgia, arthralgia, nausea, dizziness, chills, and fever—for 7 days. Reports of unsolicited AEs were collected at the study visit 28 days after each vaccination. Severity of any reaction or AE was graded according to the FDA Toxicity Grading Scale^15^ (Supplementary Table S2, p.6). All serious AEs (SAEs), medically attended AEs (MAAEs), AEs of special interest (AESIs), and AEs leading to study termination were recorded throughout the duration of the study. AESIs were AEs potentially associated with COVID-19 or COVID-19 vaccination as defined by the Safety Platform for Emergency vACcines (SPEAC, 2022).^16^ A central cardiac adjudication committee (CCAC) met to assess any potential cases of myocarditis or pericarditis occurring after vaccination in accordance with the Brighton Collaboration case definition.^17^

**Fig. 1:**
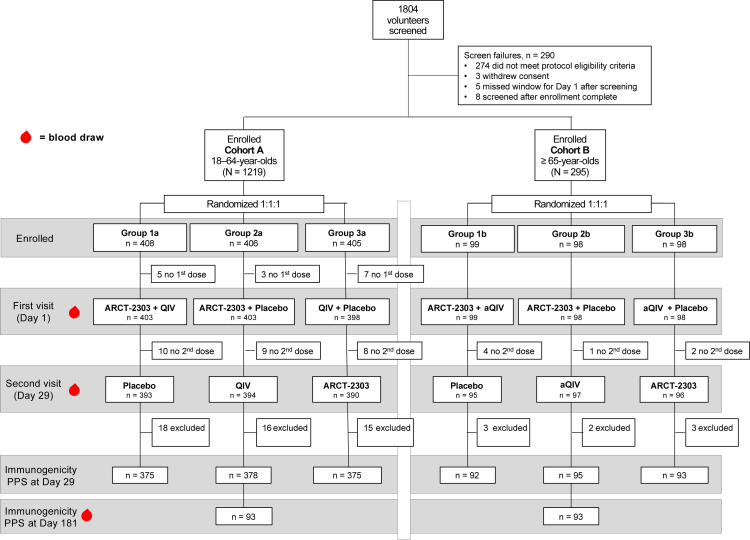
Study flow chart for the two age cohorts. Abbreviations: aQIV, adjuvanted quadrivalent influenza vaccine; PPS, per protocol set; QIV, quadrivalent influenza vaccine.

To ensure all participants received both COVID-19 and influenza vaccines while maintaining the blinded design of the study, all participants received a third vaccination (placebo, (a)QIV or ARCT-2303) after the blood draw on Day 29 (see Fig. 1).

### Vaccines

The ARCT self-amplifying mRNA vaccine construct consists of the replicase of Venezuela equine encephalitis virus in which mRNA coding for the viral structural proteins has been replaced with mRNA coding for the target antigen. ARCT-154 contained the mRNA encoding the spike glycoprotein (S protein) of the Wuhan-Hu-1 SARS-CoV-2 strain with the D614G mutation.^7^^,^^8^ ARCT-2303 is a variant vaccine in which the mRNA codes for the S-protein of the Omicron XBB.1.5-sublineage SARS-CoV-2. Both formulations contain 5 μg mRNA encapsulated in lipid nanoparticles in a final volume of 0.5 mL for injection. The QIV were Flucelvax Quad for adults aged 18–64 years, and the adjuvanted Fluad Quad (referred to as aQIV) for adults aged ≥65 years. Both influenza vaccines were the Southern Hemisphere 2024 season composition (Supplementary Material p.7) and were supplied in pre-filled syringes (0.5 mL) for intramuscular administration according to the instructions of the manufacturer (Seqirus Pty Ltd, Parkville, Victoria, Australia).

### Immunogenicity

Sera prepared immediately from blood samples drawn before the vaccinations on Days 1 and 29, and then on Day 181, were kept at −20 °C and shipped on dry ice to the immunogenicity laboratory, Vismederi (Siena, Italy). Neutralising antibodies against the Omicron XBB.1.5.6 sub-lineage were measured simultaneously in study subjects and historical samples of participants of the previously reported study ARCT-154-J01.^9^ The Omicron XBB.1.5.6 sub-lineage was used in these assays as representative of Omicron XBB 1.5 as it was available to the laboratory at the time of the study, and there is little or no immunologic assay difference within the XBB sub-lineages. The same laboratory measured haemagglutinin inhibition titres against the four influenza vaccine components. In addition, in a subset of participants, neutralising antibodies against the Wuhan (ancestral strain), Delta, and Omicron BA.2, JN.1, and KP 3.1.1 sub-lineages were tested on Days 1, 29, and 181 to characterise cross-neutralising activity and antibody persistence.

### Statistics

The analysis population for the four co-primary immunogenicity objectives was the per protocol (PP) set, which includes study participants who received all study vaccines according to the protocol and had no important protocol deviations. As fewer than 5% of participants were excluded from the modified Intended-To-Treat (mITT) population, we did not repeat the analyses on the mITT.

The success criterion of the study was the demonstration of co-primary objectives 1 and 2. If co-primary objectives 1 and 2 (GMT ratio and SCR difference) were both met, then testing would continue to the second step, which includes testing co-primary objectives 3 and 4 on Day 29.

The GMT was calculated as the mean of the antibody titres after the data were log-transformed, and then the antilog of the log mean was taken to present the results on the original scale.

The GMT ratio for each of the primary immunogenicity endpoints was assessed using an analysis of covariance (ANCOVA) model that included vaccine/treatment group as a factor and baseline antibody titre level (log-scale), age (continuous), and sex (categorical) as covariates. For objectives iii and iv, GMT ratios were also adjusted for the stratification factors: the number of previous COVID-19 vaccine doses and the composition of the last booster. The 95% Confidence Interval (CI) for GMT ratios was obtained by taking the antilog of the confidence limits for the adjusted mean difference of the logarithmically transformed assay results, calculated using t-distribution.

SCR was defined as a binary variable for participants with non-missing values at pre-vaccination and post-vaccination, if there is a post-vaccination titre ≥ 4xLLOQ (lower limit of quantification) for pre-vaccination titre <LLOQ, or there is at least 4-fold increase (post-vaccination) from pre-vaccination titre ≥LLOQ. SCR 95% CI for each group was calculated using the Clopper–Pearson method.^18^ Differences in SCRs between two groups and the 95% CI were calculated using the Mantel–Haenszel method (with factors as age group and sex).^19^ Descriptive immunogenicity analyses, including GMT, geometric mean fold rise (GMFR), and SCR, along with 2-sided 95% CIs, were produced for each group at all available time points.

The Safety Analysis set was used for all safety analyses, which comprised all participants who received their study Day 1 vaccinations. Descriptive summaries (counts, percentages) were calculated for safety and reactogenicity analysis.

Sample sizes of the treatment groups provided an overall power of at least 85% for all four primary objectives. The analysis of primary objectives i and ii with 1680 subjects who receive the ARCT-2303 booster compared with sera from 385 subjects in study ARCT-154-J01, would provide at least 97% power to demonstrate superiority of the GMT ratio (Lower Limit 95% CI [LL 95% CI > 1.0]) and noninferiority of the SCR difference (LL 95% CI > −5%). The analysis of objectives iii and iv, which compared the co-administration of ARCT-2303 and QIV, with 360 subjects per treatment group, provided at least 88% power to demonstrate noninferiority for all five comparisons (for the four influenza vaccine strains and one for ARCT-2303, with LL 95% CI >0.67 for the GMT ratios of co-administered versus standalone vaccine). An allowance for up to 10% non-evaluable subjects yielded 400 subjects per treatment group for these latter objectives. A more detailed description of the sample size, including information on adjustments for the four co-primary endpoints, is provided in the Supplementary Material, p.9. All statistical analyses were done using SAS version 9.4.

As only a small number of immunogenicity data were missing on Day 29 (≤1.7% participants in each study group), and missing samples were equally distributed across groups, no additional analyses or imputations of missing data were performed.

An independent data safety monitoring board (DSMB) had oversight of the study conduct and periodically reviewed all safety data during the trial period.

### Role of the funding source

The funder of the study was involved in the study design, data collection and analysis, and writing of the report.

## Results

### Study population demographics

The study was conducted between 27 March 2024 and 21 November 2024. Overall, 1514 adults of 1804 screened were recruited, 1219 in the 18–64 year-olds (Cohort A) and 295 in the ≥65 year-olds (Cohort B). Of these, 15 persons enrolled in Cohort A did not receive their first allocated vaccination (Fig. 1), leaving 1204 and 295 in Cohorts A and B, respectively, for the intention to treat analysis (ITT). The recruitment target for Cohort B was not reached due to the competition with COVID-19 national immunisation campaigns and a relatively small proportion of older adults who received the primary immunisation series with mRNA COVID-19 vaccines (key inclusion criteria).

Demographics show proportionally more females than males in both cohorts, 757 (62.9%) in Cohort A and 195 (66.1%) in Cohort B (Table 1). Mean ages were 37–39 years and 70 years in Cohorts A and B. Most of Cohort A were recruited in Australia (638, 53.0%), with 400 (33.2%) from the Philippines, while most of the older Cohort B were recruited in the Philippines (202, 68.5%). This is reflected in the breakdown by ethnicity, with 486 (40.4%) and 510 (42.4%) of the young adults reporting as Asian or White, respectively, while 214 (72.5%) of the older cohort reported as Asian. These demographic characteristics were evenly distributed across the respective study groups. The mean age of the historical cohort was 45 years, with more females (59.2%) than males (40.8%), 100% of whom were from Japan and reported Asian ethnicity.

**Table 1:** Demographics of the intention to treat (ITT) study population and the historical ARCT-154 population.

	Young adults 18–64 years of age			Older adults ≥65 years of age			Historical ARCT-154 cohort
COVID-19 vaccine	ARCT-2303	ARCT-2303	Placebo	ARCT-2303	ARCT-2303	Placebo	
Influenza vaccine	QIV	Placebo	QIV	aQIV	Placebo	aQIV	
	N = 403	N = 403	N = 398	N = 99	N = 98	N = 98	N = 385
**Age** (yrs)							
Mean ± SD	37.7 ± 12.0	36.8 ± 12.1	39.2 ± 11.9	69.6 ± 5.3	70.4 ± 5.4	70.4 ± 4.9	45.3 ± 11.9^b^
**Sex** n (%)							
Male	144 (35.7)	148 (36.7)	155 (38.9)	35 (35)	30 (31)	35 (36)	157 (40.8)
Female	259 (64.3)	255 (63.3)	243 (61.1)	64 (65)	68 (69)	63 (64)	228 (59.2)
**BMI**, kg/m^2^							
Mean ± SD	28 ± 6	28 ± 6	27 ± 6	26 ± 6	25 ± 5	25 ± 5	23 ± 4
**Country** n (%)							
Australia	216 (53.6)	217 (53.8)	205 (51.5)	15 (15)	11 (11)	13 (13)	Japan 385 (100)
Costa Rica	29 (7.2)	33 (8.2)	27 (6.8)	10 (10)	5 (5)	12 (12)	
Honduras	28 (6.9)	24 (6.0)	25 (6.3)	11 (11)	8 (8)	8 (8)	
Philippines	130 (32.3)	129 (32.0)	141 (35.4)	63 (64)	74 (76)	65 (66)	
**Ethnicity** n (%)							
Asian	150 (37.2)	159 (39.5)	177 (44.5)	69 (70)	77 (79)	68 (69)	385 (100)
White	179 (44.4)	173 (42.9)	158 (39.7)	9 (9.1)	8 (8)	10 (10)	0
Other^a^	74 (18.4)	71 (17.6)	63 (15.8)	21 (21)	13 (13)	20 (20)	0
**Prior SARS-CoV-2 infection**							
n (%)	25 (6.2)	30 (7.4)	31 (7.8)	2 (0.0)	0 (0.0)	0 (0.0)	101 (26.2)
**Time since last vaccine dose** (months)							
Median (min, max)	21.9 (5.1, 30.8)	21.1 (5.0, 39.4)	21.6 (5.1, 34.6)	22.8 (8.2, 32.5)	22.8 (5.1, 28.7)	21.1 (7.1, 31.2)	9.5 (4.4, 13.6)

Abbreviations: BMI, body mass index; QIV, quadrivalent influenza vaccine; SD, standard deviation.

a Includes Unknown, American Indian or Alaska Native, Native Hawaiian, Black of African American, and Multiracial.

b Includes 11 (3%) participants aged ≥65 years.

A further 27 participants in Cohort A and 7 in Cohort B did not receive their second vaccinations; and following withdrawals or exclusions for protocol violations (49 and 8 in Cohorts A and B, respectively). 1128 (93.7%) and 280 (94.9%) participants were eligible for the per protocol immunogenicity analyses, equally distributed across the respective study groups (Fig. 1).

### Immunogenicity analyses

The endpoints for the first co-primary immunogenicity objective, confirmation of the superiority of ARCT-2303 over ARCT-154, were the respective GMTs of neutralising antibodies against Omicron XBB.1.5.6; 718 (95% CI: 652–791) after ARCT-2303 compared with 263 (236–293) in the historical ARCT-154 cohort. The GMT ratio of 2.7 (95% CI: 2.3–3.2) met the prespecified criterion for superiority, with the lower limit of the 95% CI being greater than 1.0. The result of the sensitivity analysis, using the number of previous COVID-19 vaccine doses and the strain composition of the previous dose as stratification factors, was similar with a GMT ratio of 2.79 (95% CI: 2.37, 3.29).

Further, SCRs were 73.8% (349/473; 95% CI: 69.6–77.7) after ARCT-2303 and 45.5% (175/385; 40.4–50.6) after ARCT-154; a difference of 28.4% (21.8–34.9) with a lower 95% CI limit that was higher than the non-inferiority threshold of −5%, thereby also meeting the criterion for the second co-primary objective.

**Fig. 2:**
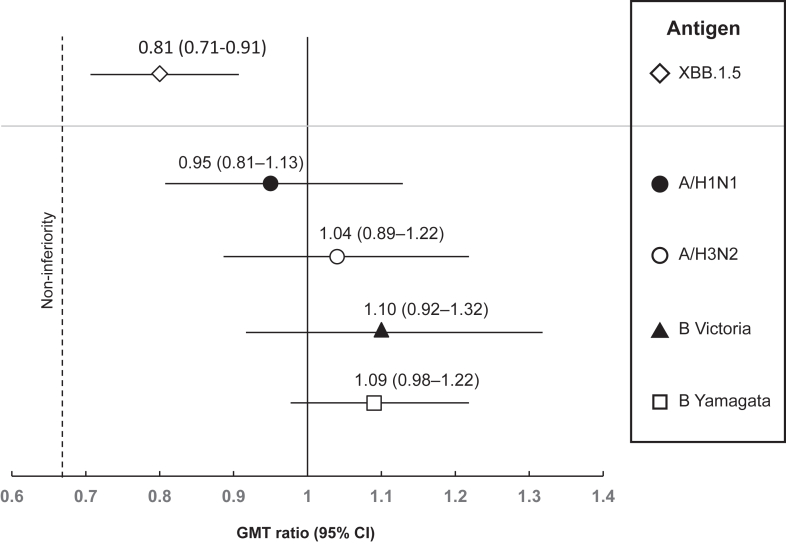
Geometric mean titre (GMT) ratios for the five primary antigens tests with 95% CI bars. Upper panels show the GMT ratio for neutralising antibodies against the Omicron XBB.1.5.6 antigen when ARCT-2303 was administered concomitantly vs separately from the quadrivalent influenza vaccine (QIV). Lower panel shows the GMT ratios for the four influenza antigens when QIV was administered concomitantly vs separately from ARCT-2303.

As the first two co-primary objectives were met, we assessed the immune responses against the four influenza strains in QIV between the co-administered group (1a) and the separately administered group (3a) in young adults. Fig. 2 shows that when QIV was administered concomitantly with ARCT-2303 vaccine, the response was non-inferior to that observed when QIV was administered alone, with the lower limits of the 95% CIs of the GMT ratios for all four influenza strains higher than the inferiority threshold of 0.67.

Table 2 confirms that concomitant administration with ARCT-2303 had no impact on the immune response to the four quadrivalent influenza vaccine antigens, whether the non-adjuvanted vaccine given to the young adults in Groups 1a and 3a, or the adjuvanted vaccine given to older adults in Groups 1b and 3b. Baseline HAI antibody titres against A/H1N1 and A/H3N2 were higher in the older cohort, but immune responses were similar in terms of mean-fold increases in both age groups (data not shown). A high proportion (94–100%) of all groups achieved HAI titres ≥40 against both A strains.

**Table 2:** Parameters of the immune response haemagglutinin inhibition antibodies (HAI) at Days 1 and 29 in the different PPS study groups after receiving quadrivalent influenza vaccines concomitantly with ARCT-2303 (Groups 1a and 1b) or standalone (Groups 3a and 3b).

Influenza strain	A/H1N1		A/H3N2		B Victoria		B Yamagata	
**18–64 years**								
**Group**	**1a**	**3a**	**1a**	**3a**	**1a**	**3a**	**1a**	**3a**
**Vaccines**	**ARCT-2303 + QIV**	**Placebo + QIV**	**ARCT-2303 + QIV**	**Placebo + QIV**	**ARCT-2303 + QIV**	**Placebo + QIV**	**ARCT-2303 + QIV**	**Placebo + QIV**
N	375	376	375	376	375	376	375	376
**GMT**^a^ (95% CI)								
Day 1	38.2 (32.9–44.4)	37.4 (32.3–43.3)	32.2 (27.9–37.2)	36.2 (31.5–41.6)	13.5 (12.1–15.0)	14.9 (13.4–16.6)	28.8 (25.7–32.4)	33.6 (30.2–37.4)
Day 29	429 (374–493)	433 (381–491)	331 (291–378)	334 (295–377)	109 (95.3–125)	102 (90.0–117)	132 (119–145)	126 (115–138)
**SCR**^a^ n (%)	267 (71)	259 (69)	266 (71)	266 (71)	233 (62)	215 (57)	197 (53)	167 (44)
**HAI ≥ 40**^a^ n (%)	354 (94)	367 (98)	361 (96)	364 (97)	299 (80)	294 (78)	345 (92)	347 (92)
**≥ 65 years**								
**Group**	**1b**	**3b**	**1b**	**3b**	**1b**	**3b**	**1b**	**3b**
**Vaccines**	**ARCT-2303 + aQIV**	**Placebo + aQIV**	**ARCT-2303 + aQIV**	**Placebo + aQIV**	**ARCT-2303 + aQIV**	**Placebo + aQIV**	**ARCT-2303 + aQIV**	**Placebo + aQIV**
N	92	93	92	93	92	93	92	93
**GMT**^a^ (95% CI)								
Day 1	115 (82.9–160)	109 (78.2–153)	78.5 (56.0–110)	92.5 (67.6–127)	19.**7** (14.7–26.4)	28.8 (20.3–41.0)	25.0 (18.6–33.6)	26.6 (20.0–35.4)
Day 29	1064 (827–1369)	961 (773–1194)	475 (368–613)	453 (342–600)	121 (91.3–160)	132 (98–178)	138 (108–176)	132 (102–170)
**SCR**^a^ n (%)	59 (64)	59 (63)	50 (54)	42 (45)	47 (51)	45 (48)	48 (52)	47 (51)
**HAI ≥ 40**^a^ n (%)	92 (100)	93 (100)	90 (98)	91 (98)	76 (83)	80 (86)	84 (91)	81 (87)

Abbreviations: aQIV, adjuvanted quadrivalent influenza vaccine; CI, confidence interval; GMT, geometric mean titre; PPS, Per Protocol Set; QIV, quadrivalent influenza vaccine; SCR, seroconversion rate.

a From unadjusted calculations.

Baseline HAI titres against the B Victoria and B Yamagata strains were lower in all four groups but increased after influenza vaccination, with no observable difference in GMT between the young and older adult cohorts (Table 2). Responses in age-matched groups who received influenza vaccine concomitantly with or separately from ARCT-2303 were similar in most cases; across the four groups, 78–92% had HAI titres ≥40 against B Victoria and B Yamagata (Table 2).

For the final co-primary objective, we evaluated the non-inferiority of the immune response against Omicron XBB.1.5.6 sub-lineage elicited when ARCT-2303 was co-administered with the quadrivalent influenza vaccine (Group 1a) compared with standalone administration (Group 2a). The GMT ratio was 0.81 (95% CI: 0.71–0.91) and the lower 95% CI was higher than the inferiority threshold of 0.67 (Fig. 2), despite the magnitude of the neutralising response appearing to be lower in the co-administration group than with the separate administration (GMFR 6.7 vs 7.7, SCR 64.5% vs 73.8%).

Immunogenicity against XBB.1.5.6 was similar in both young and older adults, both when administered concomitantly with QIV and aQIV, respectively, or when administered separately (Table 3). There was a tendency to higher GMTs in the standalone groups, as noted above, while GMFR and SCR rates were essentially the same.

**Table 3:** Geometric mean titres (GMT**s**), geometric mean fold rises (GMFR**s**), and seroconversion rates (SCR**s**) of neutralising antibodies against the Omicron XBB.1.5.6 sublineage 28 days after vaccination with ARCT-2303 concomitantly with or separately from QIV in young and older adult cohorts.

Group	1a	2a
Vaccines	ARCT-2303 + QIV	ARCT-2303 + Placebo
**Young adults** 18–64 years		
N	375	378
**GMT**^a^ (95% CI)	789 (708–880)	1072 (957–1199)
**GMFR**^a^ (95% CI)	6.69 (5.98–7.49)	7.66 (6.85–8.55)
**SCR**^a^ n (%)	242 (64.5)	279 (73.8)
**Group**	**1b**	**2b**

Vaccines	ARCT-2303 + aQIV	ARCT-2303 + Placebo

**Older adults** ≥ 65 years		
N	92	95
**GMT**^a^ (95% CI)	932 (742–1170)	1420 (1126–1791)
**GMFR**^a^ (95% CI)	6.77 (5.32–8.61)	6.89 (5.42–8.77)
**SCR**^a^ n (%)	66 (72)	70 (74)

Abbreviations: CI, confidence interval; QIV, quadrivalent influenza vaccine.

a From unadjusted calculations.

Subgroup analysis by gender showed no meaningful differences in neutralising antibodies at Day 29 against XBB.1.5.6 following ARCT-2303 vaccination between males and females (Supplementary Table S3). Analysis by country, showed variations in neutralising antibodies at Day 29, with titres of 835 in Australia (n = 207) compared with 2440 in Honduras (n = 27) (Supplementary Table S4). It was notable that baseline values also differed more than > 2-fold between countries. These differences in neutralising antibody titres may reflect differences in SARS-CoV-2 exposure history across the study population.

**Fig. 3:**
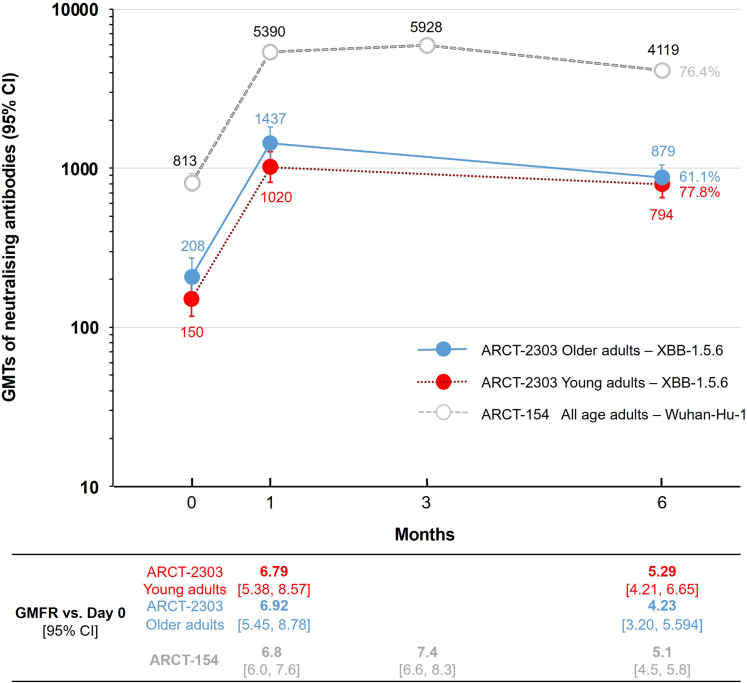
Persistence of the immune response to the vaccine antigen: neutralising antibodies against XBB.1.5.6 are shown for young and older adults who received ARCT-2303 (n = 93 per group) and against Wuhan-Hu-1 ancestral strain in all adults in the historical study after receiving ARCT-154 (n = 385).^10^ Respective GMFR are shown below the figure, and proportions of each group who still had a titre at 6 months equal to the peak titres measured 1-month post-vaccination are shown as percentages. Abbreviations: GMFR, geometric mean fold rise; GMT, geometric mean titer.

Persistence of the immune response against Omicron XBB.1.5.6 was assessed up to Day 181 in a randomly selected subset of participants who received ARCT-2303 and placebo on Day 1 (Groups 2a and 2b, n = 93 per group). Neutralising antibodies against Omicron XBB.1.5.6 persisted for 6 months after vaccination with ARCT-2303 at Day 1 in both age groups. This persistence was marked by GMFR of 5.3 in young adults and 4.2 in older adults at Day 181, which was marginally lower than GMFR at Day 29 (6.8 and 7.0) (Fig. 3). This persistence was similar to that previously observed with ARCT-154, the sa-mRNA vaccine based on the ancestral strain.^10^ At 6 months post-vaccination, GMTs represent 78.0% and 61.3% of peak GMTs measured one month post-vaccination in young and older adult groups, compared with 76.4% in the ARCT-154 study in Japanese adults.

**Fig. 4:**
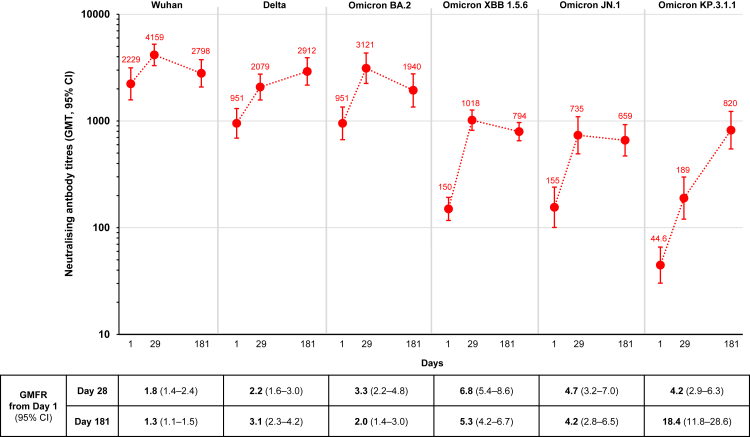
Geometric mean titres (GMTs) with 95% CI bars of neutralising antibodies against the indicated SARS-CoV-2 variant at Days 1, 29, and 181 following vaccination with ARCT-2303 (n = 35, n = 93 for XBB 1.5.6), with GMFR from Day 1 and 95% CI (all unadjusted) shown below. Abbreviations: GMFR, geometric mean fold rise; GMT, geometric mean titer; CI, confidence interval.

An exploratory immunogenicity analysis to assess cross-neutralisation of the immune response against some historical and new emergent SARS-CoV-2 strains was performed on sera from a randomly selected subset of participants who received ARCT-2303 and placebo on Day 1 (Groups 2a and 2b, n = 35). Neutralising antibodies were measured against a panel of SARS-CoV-2 variants—the ancestral Wuhan strain, Delta, Omicron BA.2, Omicron JN.1, and Omicron KP.3.1.1 (Fig. 4). Baseline titres were highest against the ancestral Wuhan strain, and then the Delta and Omicron BA.2 variants, and progressively decreased for the two most recent Omicron sub-lineages, JN.1 and the KP.3.1.1 variant which started to predominate in circulation in mid-2024 which had the lowest baseline titres. Against this background ARCT-2303 elicited a range of GMFR from 1.8 (95% CI: 1.4–2.4) for Wuhan and 2.2 (1.6–3.0) for the Delta variant, 3.3 (2.2–4.8) for Omicron BA.2 and increased to 4.7 (3.2–7.0) and 4.2 (2.9–6.3) for the more recent Omicron JN.1 and KP.3.1.1 sub-lineages. In all cases, these GMTs remained higher than baseline at Day 181, with GMFR against baseline ranging from 1.3 to 4.2, illustrating the persistence of the immune response. But the GMT of neutralising antibodies against KP.3.1.1 displayed a greater than 4-fold increase between Days 29 and 181, with a final GMFR of 18.4 (95% CI: 11.8–28.6). We observed > 3-fold increases in antibody titres against Omicron KP.3.1.1 subvariant between Days 29 and 181 in 17/35 participants. Similar increases in antibody titres were observed also in 8 of 17 participants against Omicron JN.1, 4 of 17 participants against Delta variant, 2 of 17 participants against Omicron BA.2, and 3 of 12 participants against Omicron XBB.1.5. The results may indicate that a significant number of study participants might have asymptomatic SARS-CoV-2 infection caused by dominant circulating strain (KP.3.1.1) between Days 29 and 181. This natural infection also induced cross-reactive neutralising antibodies against other SARS-CoV-2 strains. In a small subset (n = 9) of placebo recipients, there were no changes in GMTs up to Day 29, but these then responded to the dose of ARCT-2303 they received on Day 29 with an increase to GMTs similar to those in the initially vaccinated group (not shown).

### Safety and reactogenicity

**Fig. 5:**
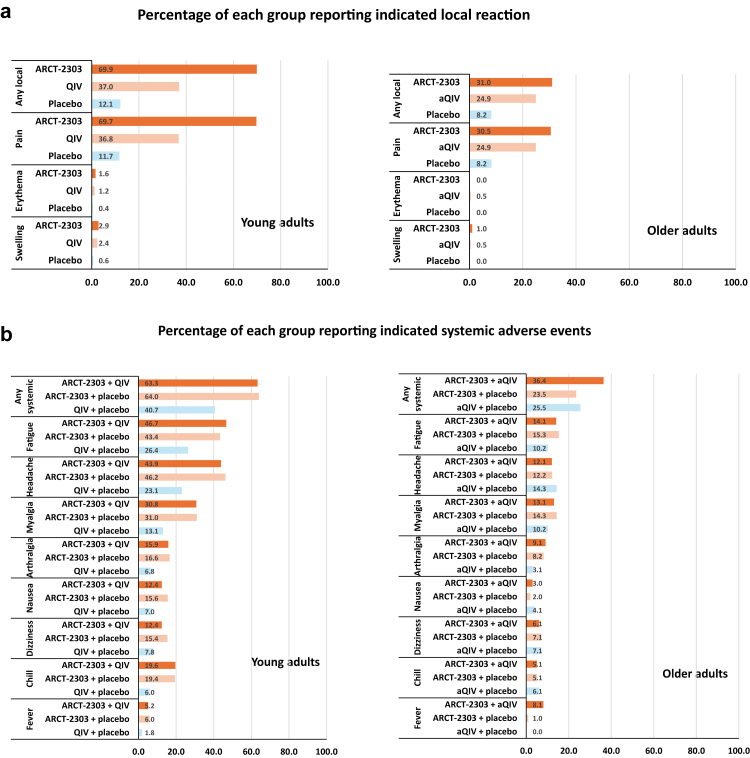
a. Solicited local reactions occurring within 7 days after ARCT-2303 or influenza vaccines in the young and older adult study cohorts. The local reactions at the injection site for ARCT-2303, (a)QIV, and placebo from co-administered and the standalone groups were pooled, as frequencies were similar between the treatment groups. Abbreviations: aQIV, adjuvanted quadrivalent influenza vaccine; QIV, quadrivalent influenza vaccine. b. Solicited systemic adverse events occurring in the 7 days after ARCT-2303 or influenza vaccines in the young and older adult study cohorts.

Solicited local reactions occurring in the 7 days after the first vaccinations in the young and older adult cohorts are illustrated in Fig. 5a. The most frequent reactions observed after both ARCT-2303 and the quadrivalent influenza vaccines, were mild or moderate pain at the injections site, with frequencies unaffected by concomitant administration of the other vaccine (data not shown). Erythema and swelling were infrequent, occurring in fewer than 3% of participants.

Systemic solicited adverse events were less frequent but consistently occurred more frequently after ARCT-2303 than influenza vaccination and were unaffected by separate or concomitant administration of the two vaccines (Fig. 5b). The most frequent systemic AEs were fatigue, headache and myalgia. All such adverse events were transient and resolved within the reporting period.

Unsolicited AEs were reported by 17–20% of young adults and 8–16% of older participants within 28 days after the first vaccinations (Table 4). The related AEs occurred with similar incidences across vaccine groups, 2.5–6.5% in the young adults and 1–2% in the older adults. Few adverse events (<1%) were assessed as severe. Serious adverse events were relatively infrequent (1–2% among young adults and 2–5% among older participants).

**Table 4:** Safety in each of the study groups and two age cohorts as numbers (%) of participants reporting adverse events—unsolicited AE within 28 days of the first vaccination (Day 1–Day 29), SAE, MAAE, AESI and fatalities over the whole study period (Day 1–Day 181).

	Young adults 18–64 years of age			Older adults ≥65 years of age		
COVID-19 vaccine Influenza vaccine	ARCT-2303 QIV	ARCT-2303 Placebo	Placebo QIV	ARCT-2303 aQIV	ARCT-2303 Placebo	Placebo aQIV
Incidence of events, n (%)	N = 403	N = 403	N = 398	N = 99	N = 98	N = 98
**Unsolicited AEs** (within Days 1–29)	79 (19.6)	81 (20.1)	68 (17.1)	13 (13)	8 (8)	16 (16)
Severe unsolicited AEs	3 (0.7)	3 (0.7)	2 (0.5)	0	0	0
Unsolicited AE related to vaccination	26 (6.5)	18 (4.5)	10 (2.5)	1 (1)	1 (1)	1 (2)
**SAEs**	7 (1.7)	5 (1.2)	6 (1.5)	2 (2)	3 (3)	5 (5)
SAE related to vaccination	0	0	1 (0.3)	0	1 (1)	0
**MAAEs**	103 (25.6)	95 (23.6)	100 (25.1)	19 (19)	13 (13.3)	19 (19.4)
MAAE related to vaccination	4 (1.0)	2 (0.5)	6 (1.5)	2 (2)	0	4 (4)
**AESIs**	1 (0.2)	1 (0.2)	3 (0.8)	1 (1)	1 (1)	1 (1)
AEs of special interest related to vaccination	0	0	1 (0.3)	0	1 (1)	0
**AEs leading to withdrawal**	0	1 (0.2)	0	0	0	1 (1)
**AEs with fatal outcome**	0	0	0	0	0	1 (1)

Abbreviations: AE, adverse event; AESI, adverse event of special interest; aQIV, adjuvanted quadrivalent influenza vaccine; MAAE, medically attended adverse event; QIV, quadrivalent influenza vaccine; SAE, serious adverse event.

Two SAEs (pericarditis and myopericarditis), one in each of the age cohorts, were considered to be related to vaccination by investigators (pericarditis after QIV vaccination and myopericarditis 60 days after ARCT-2303 and 34 days after aQIV vaccination). One fatal SAE (cardiogenic shock) was reported by an older participant, who initially received aQIV and placebo, 139 days after receiving the last study vaccination (ARCT-2303). This event was categorised by the investigator as not related to vaccination. There was one other withdrawal from the study due to an adverse event in the young adult group by a participant who initially received ARCT-2303 and placebo and had a glioblastoma 106 days after the last study vaccination (QIV); the event was categorised by the investigator as not related to vaccination.

A total of 12 AESIs were reported by eight participants. These included three cases of pericarditis or myopericarditis. The two pericarditis events were assessed by the CCAC as ‘probable pericarditis’ based on a review of medical records. One event of pericarditis was reported in a young adult seven days after vaccination with QIV and placebo (also reported as a vaccination-related SAE by the investigator). This event was resolved with treatment, and the subsequent scheduled vaccination with ARCT-2303 was not administered. A second case of pericarditis was reported in a young adult with a history of pericarditis; the event occurred 88 days after vaccination with ARCT-2303, was assessed as not related to vaccination, and was resolved with treatment. An event of myopericarditis was reported in an older adult with no cardiac history who also presented with acute myocardial infarction. The event occurred 60 days after receiving ARCT-2303 and 34 days after vaccination with aQIV and was also reported as a vaccination-related SAE by the investigator. The event resolved with sequelae of cardiac failure, which was ongoing and resolving at the end of the study. The event was assessed by the CCAC as ‘definitive myocarditis’ and ‘definitive pericarditis’.

## Discussion

New vaccines, particularly those using mRNA to encode for the S-protein of the SARS-CoV-2 virus, played a major role in controlling the COVID-19 pandemic. But the continuing emergence of new variant strains of SARS-CoV-2 with mutations in the S-protein changing the antigenicity has resulted in decreased vaccine efficacy. These factors necessitate ongoing booster vaccinations and the development of updated variant vaccines that not only target the new strains but also improve on the immunogenic efficiency of the original. ARCT-2303 is a sa-mRNA vaccine that targets the S-protein of the Omicron XBB.1.5-sublineage of SARS-CoV-2. ARCT-2303 is based on the ancestral strain encoding vaccine, ARCT-154, which has been shown to elicit a superior immune response compared with conventional mRNA vaccines with a longer duration of the response and improved cross-reactivity against other strains when administered as a booster dose.^2^, ^3^, ^4^, ^5^, ^6^, ^7^ The vaccine is currently licenced in Japan and the EU under the trade name of Kostaive®.

This study confirms that ARCT-2303 is highly immunogenic as a booster dose in adults previously vaccinated with an mRNA COVID vaccine and with an acceptable tolerability and safety profile. The immune response to ARCT-2303 was superior to that of ARCT-154 when measured as neutralising antibodies against the Omicron XBB.1.5.6 variant. Whether ARCT-2303 was administered concomitantly with an age-appropriate quadrivalent influenza vaccine or separately, the immune response was robust in all ages, and any slight differences in neutralising GMTs are unlikely to be clinically significant. Previously, in the pivotal efficacy study of ARCT-154 (encoding the ancestral strain) we used the same assays to assess immune responses after a 2-dose primary vaccination series in SARS-CoV-2 naïve adults and confirmed that a postvaccination titre of 368 (95% CI: 329–411) was associated with 56% efficacy against COVID-19 disease caused by heterologous vaccine strains (mainly Delta) and 95% efficacy against severe disease. Antibody titres observed following a booster dose of ARCT-2303 were significantly higher in both settings (co-administration and separate administration).

Concomitant vaccination with ARCT-2303 has no impact on the immunogenicity of the influenza vaccines. Concomitant vaccination did not affect the overall safety profile, with no increase in reactogenicity or frequency of serious adverse events. This means that ARCT-2303 can be safely used to meet the current recommendation of annual seasonal booster vaccination together with influenza vaccines, particularly in the target age group of 65 years and older.^20^

We found that high titres of neutralising antibodies against XBB.1.5.6 persisted up to six months after vaccination. The persistence of the response was similar in both young and older adults. The kinetics of antibody waning were similar to those observed for the ancestral strain encoding sa-mRNA vaccine, ARCT-154.^10^

We also demonstrated that the ARCT-2303 induced a broad immune response, with neutralising antibodies against a panel of historical and newly emergent SARS-CoV-2 strains. It was notable that the immediate response against the most recent subvariant tested, Omicron KP.3.1.1, when measured 28 days post-vaccination, displayed a GMFR of 4.2 (95%: 2.9–6.3), which was further increased six months post-vaccination when the GMFR was 18.4 (95%: 11.8–28.6). This was most probably due to the response to Omicron KP.3.1.1 being further boosted during the 5-month period from Day 29 to Day 181, presumably through natural exposure to the circulating virus as 49% of those assessed displayed a 3-fold or greater increase in neutralising titre between Days 29 and 181. Some of these individuals have also demonstrated an increase in antibody titres against other SARS-CoV-2 variants, but with a lower magnitude. As no participant reported COVID-19 disease in the subset of subjects selected for cross-neutralisation testing, we can speculate that ARCT-2303 prevented symptomatic COVID-19 disease caused by this variant but not asymptomatic infection, which naturally boosted the immune response.

Safety surveillance was focused on detecting possible myocarditis and pericarditis cases, and all study participants who reported chest pain or shortness of breath during the study period underwent a comprehensive cardiovascular assessment. In total, two cases of pericarditis and one case of myopericarditis were diagnosed in the study. One of these cases was reported within a biologically plausible window (28 days) for cardiac events associated with COVID-19 vaccination or SARS-CoV-2 infection.^21^ This case occurred 7 days after QIV vaccination, and no other vaccines were administered to this individual. The onset time for two other cases (60- and 88-days post ARCT-2303 dose) does not indicate a potential association of these events with vaccination.

Overall, the immunogenicity, reactogenicity and safety profile seen in this study with ARCT-2303 was consistent with that seen with the ancestral strain sa-mRNA vaccine.^8^, ^9^, ^10^, ^11^, ^12^^,^^22^ There are several limitations to our study. Immunogenicity has only been measured as the neutralising antibody response, and no attempt has been made to assess cellular immunity. However, the primary indicator of protection afforded by SARS-CoV-2 vaccines appears to be the humoural response, which has been found to correlate with the measured protective efficacy of several COVID-19 vaccines.^23^^,^^24^ This immediate response may be a more important factor, particularly in the context of proposed annual revaccination with SARS-CoV-2 vaccines. Another issue is that for ethical reasons we administered a dose of ARCT-2303 at Day 29 in those originally assigned to receive placebo on Day 1, which did not allow us to track the immune response to six months in the absence of ARCT-2303. Thus, we cannot confirm that the observed increase in titres against Omicron KP.3.1.1 between Days 29 and 181 was due to natural exposure to the circulating virus rather than a vaccine-induced immune response. We did not meet the recruitment target for Cohort B but this had no impact on the overall study power, although it limits our ability to provide a comprehensive descriptive analysis of immunogenicity and safety in older adults.

In summary, we showed that the sa-mRNA vaccine, encoding the XBB.1.5 variant, induces a robust immune response against the vaccine variant and broad cross-neutralisation of historical and new emergent SARS-CoV-2 strains. The magnitude of the immune response and relative immunogenicity compared with the ancestral strain-containing vaccine are consistent with regulatory requirements. We also demonstrated that the sa-mRNA COVID-19 vaccine can be co-administered with licenced age-appropriate inactivated influenza vaccines in adults with no impact on the safety or immunogenicity of either vaccine. This new data further supports the inclusion of the vaccine in the COVID-19 immunisation programs.

## Contributors

C.B, C.V, R.B, J.v.B, and I.S participated in the design, protocol development, and conduct of the study. M.L.G, J.L.W, X.L, R.B, C.B, C.V, and I.S had access to and verified the underlying data reported in the manuscript. M.L.G, J.L.W, C.V, C.B, I.S, and M.H reviewed the study results and provided a critical review of the manuscript. M.L.G, C.T, L.B, J.C.V, M.E.M, P.N, S.D, and M.N oversaw and participated in data acquisition, study management, and study operations. X.L, R.B, C.B, and C.V participated in the data analysis plan and data analysis. C.B and H.J oversaw the laboratory testing. All authors reviewed and commented on all versions of the manuscript. All authors gave final approval of the version to be published.

## Data sharing statement

The data generated in this study will be made available to suitably qualified scientific researchers who make a request to the study sponsor with a suitable protocol for a valid research project.

## Declaration of interests

C Baccarini, H Jin, R Bugarini, X Liu, C Verhoeven, and I Smolenov are full-time employees of the vaccine manufacturer, Arcturus Therapeutics, and hold the company's stock options. M Hohenboken and J van Boxmeer are full-time employees of the vaccine marketing authorisation holder, CSL and own CSL's stock. JL Walson is an independent consultant working for Arcturus Therapeutics and a member of the Scientific Advisory Board of Arcturus Therapeutics. Dr. ML Giles, C Tabora, L Barrientos, JC Vargas, ME Montellano, P Nguyen, S Deshmukh, and M Neville are investigators of the study ARCT-2303-01 and received fees for study participation.

## Appendix A Supplementary data

Protocol

Statistical Analysis Plan

Manuscript_clean version_24 July 2025.docx (pg no: 1–12)
