# Supplementary material for: Immunogenicity and safety of self-amplifying mRNA COVID-19 vaccine (ARCT-2303), with or without co-administration of seasonal inactivated influenza vaccine in adults: a phase 3, randomised, controlled, observer-blind, multicentre study
Source: eClinicalMedicine. 2025 Aug 20;87:103428. doi: 10.1016/j.eclinm.2025.103428 (PMC12396474; doi:10.1016/j.eclinm.2025.103428)
Supplement: Protocol [file mmc1.pdf]

CLINICAL STUDY PROTOCOL

Protocol Title: A Phase 3, Multicenter, Observer-blind, Randomized, Controlled Study to Evaluate the Immunogenicity, Reactogenicity, and Safety of a Self-Amplifying RNA COVID-19 Vaccine (ARCT-2303), Administered Concomitantly with Quadrivalent Influenza Vaccines, in Adults

Short Title: Immunogenicity and Safety Study of Self-Amplifying RNA COVID-19 Vaccine, Administered Concomitantly with Quadrivalent Influenza Vaccines, in Adults

Protocol Number:	ARCT-2303-01
Vaccine(s):	Investigational ARCT-2303 COVID-19 vaccine<br>Flucelvax Quadrivalent® (Influenza vaccine, surface antigen, inactivated, prepared in cell cultures)<br>Fluad Quadrivalent® (Influenza vaccine, surface antigen, inactivated, adjuvanted)
Study Phase:	Phase 3
Sponsor Name:	Arcturus Therapeutics, Inc.
Legal Registered Address:	10628 Science Center Dr #250, San Diego, CA 92121
Version:	2.0 (Amendment 2)
Date of Protocol:	21 February 2024

CONFIDENTIAL

May not be used, divulged, published or otherwise disclosed without written consent of Arcturus Therapeutics, Inc.

SPONSOR SIGNATORY

Protocol accepted and approved by:

Redacted	Date
Redacted	Date
Redacted	Date
Redacted	Date
Redacted	Date

TABLE OF CONTENTS

SPONSOR SIGNATORY 2
TABLE OF CONTENTS 3
TABLE OF TABLES 7
TABLE OF FIGURES 7
LIST OF ABBREVIATIONS 8
1 PROTOCOL SYNOPSIS 10
1.1 Study Design 25
1.2 Schedule of Assessments 26
2 INTRODUCTION 29
2.1 Background 29
2.1.1 Nonclinical Studies 31
2.1.2 Clinical Studies 31
2.2 Study Rationale 33
2.2.1 Scientific Rationale for the Study Design 34
2.2.2 Justification for Dose and Dose Regimen 35
2.3 Benefit/Risk Assessment 35
2.3.1 Risk Assessment 35
2.3.2 Benefit Assessment 38
2.3.3 Overall Benefit/Risk Conclusion 38
3 OBJECTIVES, ENDPOINTS AND ESTIMANDS 39
4 STUDY DESIGN 39
4.1 Overall Design 39
4.2 End of Study Definition 41
5 STUDY POPULATION 46
5.1 Inclusion Criteria 46
5.2 Exclusion Criteria 46
5.3 Contraindications for the Second Vaccination 48
5.4 Prior and Concomitant Medications 48
5.5 Prohibited Medications 50
5.6 Screen Failures 50

May not be used, divulged, published or otherwise disclosed without written consent of Arcturus Therapeutics, Inc.

6 STUDY VACCINES 51
6.1 Study Vaccine 51
6.2 Vaccine Preparation, Handling, Storage and Accountability 51
6.2.1 Vaccine Preparation 51
6.2.2 Vaccine Handling 51
6.2.3 Vaccine Storage 52
6.2.4 Vaccine Accountability 52
6.2.5 Vaccine Administration 52
6.2.6 Errors in Study Vaccine Administration 53
6.3 Licensed Study Vaccines 53
6.4 Measures to Minimize Bias: Randomization and Blinding 53
6.5 Vaccine Compliance 54
6.6 Treatment after the End of the Study 54
7 PARTICIPANT WITHDRAWAL FROM THE STUDY 55
7.1 Discontinuation of Study Treatment 55
7.2 Participant Withdrawal from the Study 55
7.3 Lost to Follow-up 56
8 STUDY ASSESSMENTS AND PROCEDURES 57
8.1 Informed Consent 57
8.2 Demographics 57
8.3 Physical Examination and Vital Signs 57
8.4 Pregnancy Testing 58
8.5 Rapid COVID-19 Antigen Test 58
8.6 ECG/Cardiac Enzymes Test 58
8.7 Inclusion and Exclusion Criteria 58
8.8 Randomization 59
8.9 Vaccination 59
8.10 Immunogenicity Assessments 59
8.10.1 Blood Volumes and Serological Testing 60
8.10.2 Biological Sample Retention and Destruction 61
8.11 Safety Assessments 61

8.12 Schedule of Assessments 62
8.12.1 Screening (Day 1) 62
8.12.2 Pre-vaccination Procedures (Day 1 and Day 29) 62
8.12.3 Vaccination and Post-vaccination Procedures (Day 1 and Day 29) 62
8.12.4 Non-vaccination Site Visit Procedure (Day 57) 64
8.12.5 Phone Contact (Day 91) 64
8.12.6 End-of-study (Day 181) or Early Termination (ET)/Last Visit Procedures 64
8.12.7 Unscheduled Visits 65
8.12.8 Post-study Procedures 65
9 ADVERSE EVENTS 66
9.1 Definitions 66
9.1.1 Definition of an Adverse Event 66
9.1.1.1 Events Meeting the AE Definition 66
9.1.1.2 Events NOT Meeting the AE Definition 66
9.1.2 Definition of an SAE 67
9.1.3 Recording of AEs and SAEs 68
9.1.4 Assessment of Intensity 68
9.1.5 Assessment of Causality 68
9.1.6 Assessment of Outcome of AE 69
9.1.7 Time Period and Frequency for Collecting Adverse Event and Serious Adverse Event Information 69
9.1.8 Method of Detecting AEs and SAEs 70
9.1.9 Solicited and Unsolicited Adverse Events 70
9.1.10 Follow-up of AEs and SAEs 71
9.1.11 Regulatory Reporting Requirements for SAEs 72
9.1.12 Reporting of AESIs and SAEs 72
9.1.12.1 AESI and SAE Reporting via the eCRF Page in EDC 72
9.1.12.2 AESI and SAE Reporting via Paper AESI/SAE Report Form 72
9.1.13 Pregnancy 73
9.1.14 Adverse Events of Special Interest 73
9.1.14.1 Suspected Myocarditis and Pericarditis Cases 74
9.1.15 Medically Attended Adverse Events 74

May not be used, divulged, published or otherwise disclosed without written consent of Arcturus Therapeutics, Inc.

9.2 Treatment of Overdose 74
9.3 Genetics 74
10 STATISTICAL CONSIDERATIONS 75
10.1 Statistical Hypotheses and Hypothesis Testing 75
10.2 Sample Size Determination 77
10.3 Statistical Analyses 79
10.3.1 Analysis of Demographic and Baseline Characteristics 80
10.3.2 Immunogenicity Analyses 81
10.3.3 Safety Analyses 83
10.4 Interim Analyses and Analysis Timing 83
10.4.1 Final Analyses – First Analysis 83
10.4.2 Final Analyses – Second Analysis 84
10.5 Data Safety Monitoring Board and Central Cardiac Adjudication Committee 84
11 REFERENCES 85
12 APPENDICES 87

TABLE OF TABLES

Table 1 Schedule of Assessments 26
Table 2 Important Risks Associated with ARCT-2303 Vaccine and Mitigation Strategy 36
Table 3 Risks Related to Study Procedures 38
Table 4 Objectives, Endpoints and Estimands 42
Table 5 Study Vaccine Details –ARCT-2303 51
Table 6 Testing Plan for ARCT-2303-01 61
Table 7 Severity Grading for Solicited Local Reactions and Systemic Adverse Events 71
Table 8 Analysis Sets 79
Table 9 Immunogenicity Analyses 81
Table 10 Safety Analyses 83
Table 11 Highly Effective Contraceptive Methods 89
Table 12 Study Administrative Structure 93
Table 13 List of Adverse Events of Special Interest Relevant to COVID-19 (Guidance Document from Safety Platform for Emergency vACcines [SPEAC]) 96

TABLE OF FIGURES

Figure 1 Study Design for ARCT-2303-01 25

LIST OF APPENDICES

Appendix 1 Contraceptive Guidance and Collection of Pregnancy Information[17] 88
Appendix 2 Regulatory, Ethical, and Study Oversight Considerations 91
Appendix 3 Adverse Events of Special Interest 96
Appendix 4 Protocol Amendments 98
Appendix 5 Investigator Signature 101

CONFIDENTIAL

May not be used, divulged, published or otherwise disclosed without written consent of Arcturus Therapeutics, Inc.

LIST OF ABBREVIATIONS

AE	adverse event
AESI	adverse event of special interest
ANCOVA	analysis of covariance
CDC	Centers for Disease Control and Prevention
BMI	body mass index
CI	confidence interval
CIOMS	Council for International Organizations of Medical Sciences
CONSORT	Consolidated Standards of Reporting Trials
CoV	Coronavirus
COVID-19	coronavirus disease 2019
CSR	clinical study report
d	Days
D	Day
DIC	disseminated intravascular coagulation
DSMB	data safety monitoring board
eCRF	electronic case report form
EDC	electronic data capture
ET	early termination
FDA	US Food and Drug Administration
FSH	follicle-stimulating hormone
GCP	Good Clinical Practice
GMFR	geometric mean fold rise
GMP	Good Manufacturing Practice
GMT	geometric mean titer
HIV	human immunodeficiency virus
HRT	hormone replacement therapy
IB	investigator's brochure
IA	interim analysis
ICF	informed consent form
ICH	International Council for Harmonisation
ICU	intensive care unit
IEC	independent ethics committee
IM	Intramuscular

CONFIDENTIAL

May not be used, divulged, published or otherwise disclosed without written consent of Arcturus Therapeutics, Inc.

IMP	investigational medicinal product
IRB	institutional review board
IRT	interactive response technology
IUD	intrauterine device
IUS	intrauterine hormone-releasing system
LL	lower limit
LLOQ	lower limit of quantitation
MAAE	medically attended adverse event
MedDRA	Medical Dictionary for Regulatory Activities
NI	Noninferiority
NHP	nonhuman primate
PC	phone contact
PP	Per Protocol Set
PT	preferred term
RT-PCR	reverse transcription polymerase chain reaction
SAE	serious adverse event
SAF	Safety Set
SAP	statistical analysis plan
SARS-CoV-2	severe acute respiratory syndrome coronavirus 2
SCR	seroconversion rate
SD	standard deviation
SoA	schedule of assessments
SOC	system organ class
SPEAC	Safety Platform for Emergency vACcines
SUSAR	suspected unexpected serious adverse reaction
US	United States of America
Vac	Vaccination
VE	vaccine efficacy
VED	vaccine-enhanced disease
VNA	virus neutralizing assay
VRBPAC	Vaccines and Related Biological Products Advisory Committee

CONFIDENTIAL

May not be used, divulged, published or otherwise disclosed without written consent of Arcturus Therapeutics, Inc.

1 PROTOCOL SYNOPSIS

<table>
<tr><td>Sponsor:
Arcturus Therapeutics, Inc.
10628 Science Center Dr #250
San Diego, CA 92121</td><td>Protocol number:
ARCT-2303-01</td><td>ClinicalTrials.gov:

EudraCT:</td><td>Date:
21 February 2024</td></tr>
<tr><td colspan="4">Title of Study: A Phase 3, Multicenter, Observer-blind, Randomized, Controlled Study to Evaluate the Immunogenicity, Reactogenicity, and Safety of a Self-Amplifying RNA COVID-19 Vaccine (ARCT-2303), Administered Concomitantly with Quadrivalent Influenza Vaccines, in Adults</td></tr>
<tr><td colspan="4">Publication (reference): Not applicable.</td></tr>
<tr><td colspan="2">Duration of Participation: Approximately 6 months for each participant.</td><td colspan="2">Clinical Phase: Phase 3</td></tr>
<tr><td colspan="4">Background and Rationale:
Severe acute respiratory syndrome coronavirus 2 (SARS-CoV-2) infection leads to a cluster of respiratory illnesses, collectively called coronavirus disease 2019 (COVID-19), similar to those caused by SARS-CoV. COVID-19 has been associated with a high transmission rate, and severe cases require admission to hospital intensive care units (ICUs) with the need for mechanical ventilation and associated high mortality. As of March 2023, over 760 million cases of COVID-19 have been confirmed worldwide and over 6.8 million people have died (https://covid19.who.int/).
Several COVID-19 vaccines, based on the ancestral strain of the SARS-CoV-2 virus, were developed, authorized, and broadly used to prevent the spread of the COVID-19 pandemic and reduce morbidity and mortality associated with COVID-19.
Arcturus Therapeutics, Inc. (Arcturus) developed ARCT-154, a candidate vaccine for prophylaxis of COVID-19. The vaccine is composed of a lipid nanoparticle (LNP) formulation of an mRNA replicon based upon Venezuelan equine encephalitis virus (VEEV) in which RNA encoding for the VEEV’s structural proteins has been replaced with RNA encoding for the SARS-CoV-2 full-length spike (S) glycoprotein.
ARCT-154 vaccine, containing 5 µg of mRNA encoding ancestral SARS-CoV-2 strain with a D614G mutation, was assessed in a randomized, controlled Phase 1/2/3 clinical study (ARCT-154-01). The study evaluated the safety, efficacy, and immunogenicity of the candidate vaccine, administered as a primary vaccination series in adults ≥18 years of age. As part of that study execution, in Phase 3b, over 12,700 younger adults (18 years of age to <60 years) and over 2,600 older adults (≥60 years of age) have received at least one dose of the ARCT-154 vaccine during the primary vaccination. ARCT-154 demonstrated 56.6% efficacy (95% CI: 48.7% - 63.3%) against COVID-19 disease of any severity and 95.3% efficacy (95% CI: 80.5% - 98.9%) against severe COVID-19. In the immunogenicity analysis at Day 57, the study found that 95.9% (95%CI: 93.4%-97.6%) of the ARCT-154 recipients achieved neutralizing antibody seroconversion after 2 doses as measured by a microneutralization assay. In total, 16,393 study participants received at least one dose of the ARCT-154 vaccine, 15,139 participants received a 2-dose vaccination series, and 483 participants received three doses of the vaccine per study design. ARCT-154 had an acceptable safety profile with no major safety concerns raised from available data.
In study ARCT-154-J01, a single dose of the ARCT-154 vaccine induced a robust booster response against ancestral strain and multiple variants of concerns in individuals who previously received primary vaccination with authorized mRNA vaccines.
To extend the protection against new emergent variants of SARS-CoV-2 and to address the recommendations of public health agencies for booster vaccine composition, Arcturus developed the ARCT-2303 vaccine, which contains 5 µg of mRNA encoding for Omicron XBB.1.5 variant spike glycoprotein.
The purpose of this study is to demonstrate that immune response induced by a booster dose of the ARCT-2303 vaccine is superior to those in individuals who received a booster dose of ARCT-154, which showed immunological noninferiority to the authorized mRNA COVID-19 vaccine (BNT162b2, Pfizer) in study ARCT-154-J01. Immunological superiority will be demonstrated against ARCT-154 in study ARCT-154-J01 for the Omicron XBB.1.5 variant strain. In addition, the immune response against epidemiologically relevant variants of SARS-CoV-2 will be evaluated.</td></tr>
</table>

CONFIDENTIAL

May not be used, divulged, published or otherwise disclosed without written consent of Arcturus Therapeutics, Inc.

As both COVID-19 and influenza vaccines are recommended to the general population in many countries, the present study is also designed to ensure that neither vaccine interferes with the safety or immunogenicity of the other vaccine when administered concomitantly in adults.

Flucelvax® Quadrivalent (Seqirus Pty Ltd.) is one of the broadly used seasonal influenza vaccines and the only influenza vaccine in use in the United States for which the vaccine viruses are grown in cell culture. This vaccine is licensed for use in individuals 6 months and older.

For individuals 65 years of age and above, there are three influenza vaccines preferentially recommended by the US CDC. They include Fluad® Quadrivalent (Seqirus Pty Ltd.), a standard-dose, inactivated influenza vaccine that contains MF59 (squalene-based oil-in-water adjuvant). An adjuvant is an ingredient added to a vaccine that helps create a stronger immune response to vaccination. Fluad Quadrivalent is licensed for use among people 65 years and older.

The Southern Hemisphere 2024 season vaccine composition for both influenza vaccines will be used in the study.

Recent studies (Domnich et al., 2022) indicate that there are no specific safety concerns raised over administration of mRNA COVID-19 vaccines (such as Pfizer's BNT162b2) in adults alongside standard dose inactivated influenza vaccines, including that with MF59 adjuvant. Concomitant vaccination with both COVID-19 and influenza vaccines should reduce the burden on the healthcare services for vaccine delivery, allowing for timely vaccine administration and protection from COVID-19 and influenza for those in need (Lazarus et al., 2021).

The study intends to demonstrate that the immune responses against the ARCT-2303 vaccine and the quadrivalent influenza vaccine, when administered concomitantly, are noninferior to immune responses induced when administered separately. These objectives will be demonstrated in individuals 18 to 64 years of age who receive Flucelvax® Quadrivalent. Additional descriptive analysis of co-administration will be performed in participants ≥65 years of age who receive adjuvanted influenza vaccine (Fluad® Quadrivalent).

CONFIDENTIAL

May not be used, divulged, published or otherwise disclosed without written consent of Arcturus Therapeutics, Inc.

Primary Immunogenicity Objective	Endpoints	Estimands
1. To demonstrate that the ARCT-2303 vaccine, when given as a booster dose, elicits an immune response that is superior (simple superiority[a]) to those after a booster dose of ARCT-154 (in study ARCT-154-J01) as measured by GMTs of neutralizing antibodies against Omicron XBB.1.5 subvariant	SARS-CoV-2 neutralizing antibody titers against Omicron XBB.1.5 subvariant in:<br>• ARCT-2303 vaccine recipients (Groups 2a and 2b) on Day 29<br>• ARCT-154-J01 study participants who received a booster dose of ARCT-154 vaccine on Day 29	• GMT ratio of $GMT_{ARCT\text{-}2303\ (Groups\ 2a\ and\ 2b)}$ (Day 29) over $GMT_{ARCT\text{-}154\ (a\ booster\ dose)}$ (Day 29)<br>by PP Set for immunogenicity
2. To demonstrate that the ARCT-2303 vaccine, when given as a booster dose, elicits an immune response that is noninferior to those after a booster dose of ARCT-154 (in study ARCT-154-J01) as measured by SARS-CoV-2 neutralizing antibody seroconversion rate with Omicron XBB.1.5 subvariant	SARS-CoV-2 neutralizing antibody seroconversion rates against Omicron XBB.1.5 subvariant in:<br>• ARCT-2303 vaccine recipients (Groups 2a and 2b) on Day 29<br>• ARCT-154-J01 study participants who received a booster dose of ARCT-154 vaccine on Day 29	• SCR difference of $SCR_{ARCT\text{-}2303\ (Groups\ 2a\ and\ 2b)}$ (Day 29) minus $SCR_{ARCT\text{-}154\ (a\ booster\ dose)}$ (Day 29)<br>by PP Set for immunogenicity
3. To demonstrate that vaccination with Flucelvax Quadrivalent, when given concomitantly with ARCT-2303, elicits an immune response that is noninferior to that of Flucelvax Quadrivalent, when given standalone, as measured by serum Hemagglutination Inhibition (HI) assay[b] at 28 days after vaccination	HI assay titers against influenza vaccine strains on Day 29 in:<br>• Co-admin group recipients (Group 1a)<br>• Standalone Flucelvax Quadrivalent group recipients (Group 3a)	• HI geometric mean titer (GMT) ratio of $GMT_{Co\text{-}admin\ (Group\ 1a)}$ (Day 29) over $GMT_{Standalone\ Flucelvax\ (Group\ 3a)}$ (Day 29)<br>For each influenza vaccine strain (4 strains), by PP Set for immunogenicity
4. To demonstrate that vaccination with ARCT-2303, when given concomitantly with Flucelvax Quadrivalent, elicits an immune response that is noninferior to that of ARCT-2303, when given standalone, as measured by GMTs of neutralizing antibodies against Omicron XBB.1.5 subvariant at 28 days after vaccination	SARS-CoV-2 neutralizing antibody responses against Omicron XBB.1.5 subvariant on Day 29, in:<br>• Co-admin group recipients (Group 1a)<br>• Standalone ARCT-2303 group recipients (Group 2a)	• GMT ratio of $GMT_{Co\text{-}admin\ (Group\ 1a)}$ (Day 29) over $GMT_{Standalone\ ARCT\text{-}2303\ (Group\ 2a)}$ (Day 29)<br>by PP Set for immunogenicity

CONFIDENTIAL

May not be used, divulged, published or otherwise disclosed without written consent of Arcturus Therapeutics, Inc.

Secondary Immunogenicity Objectives		
1. To demonstrate that the ARCT-2303 vaccine, when given as a booster dose, elicits an immune response that is superior (super superiority[a]) to those after a booster dose of ARCT-154 (in study ARCT-154-J01) as measured by GMTs of neutralizing antibodies against Omicron XBB.1.5 subvariant	SARS-CoV-2 neutralizing antibody titers against Omicron XBB.1.5 subvariant in:<br>• ARCT-2303 vaccine recipients (Groups 2a and 2b) on Day 29<br>• ARCT-154-J01 study participants who received a booster dose of ARCT-154 vaccine on Day 29	• GMT ratio of $GMT_{ARCT\text{-}2303\ (Groups\ 2a\ and\ 2b)}$ (Day 29) over $GMT_{ARCT\text{-}154\ (a\ booster\ dose)}$ (Day 29)<br>by PP Set for immunogenicity
2. To demonstrate that the ARCT-2303 vaccine, when given as a booster dose, elicits an immune response that is superior to those after a booster dose of ARCT-154 (in study ARCT-154-J01) as measured by SARS-CoV-2 neutralizing antibody seroconversion rate with Omicron XBB.1.5 subvariant	SARS-CoV-2 neutralizing antibody seroconversion rates against Omicron XBB.1.5 subvariant in:<br>• ARCT-2303 vaccine recipients (Groups 2a and 2b) on Day 29<br>• ARCT-154-J01 study participants who received a booster dose of ARCT-154 vaccine, on Day 29	• SCR difference of $SCR_{ARCT\text{-}2303\ (Groups\ 2a\ and\ 2b)}$ (Day 29) minus $SCR_{ARCT\text{-}154\ (a\ booster\ dose)}$ (Day 29)<br>by PP Set for immunogenicity
3. To assess the immunogenicity of ARCT-2303 vaccine when administered with or without Flucelvax Quadrivalent, as measured by virus neutralization assay	SARS-CoV-2 neutralizing antibody responses against Omicron XBB.1.5 subvariant on Days 1, 29, and 181 (a subset of samples):<br>• Co-admin group recipients (Group 1a)<br>• Standalone ARCT-2303 group recipients (Group 2a)	• GMTs<br>• GMFRs (post/pre-vaccination)<br>• SCRs[b]<br>• Proportion of participants with antibody titer ≥ LLOQ<br>by PP Set for immunogenicity
4. To assess the immunogenicity of Flucelvax Quadrivalent when administered with or without ARCT-2303 as measured by HI assay[c]	HI assay titers against influenza vaccine strains on Day 1 and Day 29, in:<br>• Co-admin group recipients (Group 1a)<br>• Standalone Flucelvax Quadrivalent group recipients (Group 3a)	• GMTs<br>• SCRs<br>• GMFRs (post/pre-vaccination)<br>• Proportion of participants with HI titer ≥1:40<br>For each influenza vaccine strain, by PP Set for immunogenicity
5. To assess the immunogenicity of ARCT-2303 vaccine, when administered with or without Fluad Quadrivalent, as measured by virus neutralization assay, in participants ≥65 years of age	SARS-CoV-2 neutralizing antibody responses against Omicron XBB.1.5 subvariant on Days 1, 29, and 181:<br>• Co-admin group recipients (Group 1b)<br>• Standalone ARCT-2303 group recipients (Group 2b)	• GMTs<br>• GMFRs (post/pre-vaccination)<br>• SCRs[b]

CONFIDENTIAL

May not be used, divulged, published or otherwise disclosed without written consent of Arcturus Therapeutics, Inc.

		• Proportion of participants with antibody titer ≥ LLOQ<br>by PP Set for immunogenicity
6. To assess the immunogenicity of Fluad Quadrivalent when administered with or without ARCT-2303, as measured by HI assay,[c] in participants ≥65 years of age	HI assay titers against influenza vaccine strains on Day 1 and Day 29, in:<br>• Co-admin group recipients (Group 1b)<br>• Standalone Fluad Quadrivalent group recipients (Group 3b)	• GMTs<br>• SCRs<br>• GMFRs (post/pre-vaccination)<br>• Proportion of participants with HI titer ≥1:40<br>For each influenza vaccine strain, by PP Set for immunogenicity
**Secondary Safety Objectives**		
1. To assess the safety and reactogenicity of the study vaccines when given in co-administration or standalone	• Local and systemic AEs reported within 7 days after each study vaccination<br>• Unsolicited AEs reported within 28 days after each vaccination<br>• SAE, AEs leading to early termination from study, MAAEs, and AESIs during the entire study period (6-month follow-up)	• Proportion of participants with local and systemic solicited AEs<br>• Proportion of participants with unsolicited AEs<br>• Proportion of participants with SAEs<br>• Proportion of participants with MAAEs<br>• Proportion of participants with AESIs<br>• Proportion of participants with AEs leading to early termination from study<br>by Safety Set
**Exploratory Immunogenicity Objectives**		
1. To assess the immune response elicited by the ARCT-2303 vaccine against a panel of SARS-CoV-2 strains, as measured by VNA	SARS-CoV-2 neutralizing antibody responses with a panel of historical and new emergent SARS-CoV-2 strains on Days 1 and 29 in a subset of:<br>• Standalone ARCT-2303 group recipients (Group 2a and/or 2b)<br>• Standalone QIV group recipients (Group 3a and/or 3b)	• GMTs<br>• GMFRs (post/pre-vaccination)<br>• SCRs<br>• Proportion of participants with antibody titer ≥ LLOQ<br>by PP Set for immunogenicity
2. To assess the immunogenicity of influenza vaccine(s) when administered with or without ARCT-2303 as measured by microneutralization assay (in a subset of participants)	Microneutralization (MN) titers against influenza vaccine strains on Day 1 and Day 29, in:<br>• Co-admin group recipients (a subset of participants from Group 1a and/or 1b)	• GMTs<br>• GMFRs (post/pre-vaccination)<br>• SCRs

May not be used, divulged, published or otherwise disclosed without written consent of Arcturus Therapeutics, Inc.

	• Standalone QIV group recipients (a subset of participants from Group 3a and/or 3b)	• Proportion of participants with antibody titer ≥ LLOQ<br>For each influenza vaccine strain, by PP Set for immunogenicity

Abbreviations: AE, adverse event; AESI, adverse event of special interest; GMFR, geometric mean fold rise; GMT, geometric mean titer; HI, Hemagglutination Inhibition; LLOQ, lower limit of quantification; MAAE, medically attended AEs; MN, microneutralization; PP, per protocol; QIV, quadrivalent influenza vaccine; SARS-CoV-2, severe acute respiratory syndrome coronavirus 2; SCR, seroconversion rate; SAE, serious adverse event; VNA, virus neutralization assay.

a As per FDA guidance: "simple" superiority (margin of >1-fold for GMT ratio); "super" superiority (margin of >1.5-fold for GMT ratio). US FDA Guidance for Industry. Emergency Use Authorization for Vaccines to Prevent COVID-19. Document issued on March 31, 2022.

b SCR will not be performed on Day 181.

c In case of lack of agglutination for a specific strain using HI assay, immunogenicity for that strain will be assessed as measured by microneutralization (MN) assay as an acceptable alternative.

Seroconversion rate (SCR) is defined as the percentage of participants with a ≥4-fold increase in titer from that at Day 1 (or from LLOQ if Day 1 titer <LLOQ). For definitions of the PP set for immunogenicity and other populations for analysis, refer to Section 10.3 of protocol.

Note: Missing data will not be imputed for all the immunogenicity and safety analyses.

CONFIDENTIAL

May not be used, divulged, published or otherwise disclosed without written consent of Arcturus Therapeutics, Inc.

Study Design: This is a multicenter, observer-blind, randomized, controlled phase 3 study to evaluate the immunogenicity, reactogenicity, and safety of the investigational COVID-19 vaccine ARCT-2303 administered concomitantly with Quadrivalent Influenza Vaccines or standalone in adults who previously received at least 3 doses (a primary vaccination series and at least one booster dose) of the US-authorized mRNA COVID-19 vaccines.

The study design is provided in Figure 1 of the protocol. The schedule of assessments (SoA) is provided in Table 1 of the protocol.

Number of participants: Approximately 1680 participants will be enrolled in this study.

Study arms and vaccination schedule:

Participants who received at least 3 doses (a primary vaccination series and at least one booster dose) with a US-authorized mRNA COVID-19 vaccine ≥5 months before enrollment will be recruited in one of the two age cohorts.

Cohort A (participants 18 to 64 years of age; approximately 1200 participants)

Individuals will be randomly assigned to one of the three study groups:

- Group 1a (ARCT-2303/Flucelvax Quadrivalent, N=400): participants will receive one dose of ARCT-2303 and one dose of Flucelvax Quadrivalent (opposite arms) on Day 1, and one dose of placebo on Day 29.
- Group 2a (ARCT-2303, N=400): participants will receive one dose of ARCT-2303 and one dose of placebo (opposite arms) on Day 1, and one dose of Flucelvax Quadrivalent on Day 29.
- Group 3a (Flucelvax Quadrivalent, N=400): participants will receive one dose of Flucelvax Quadrivalent and one dose of placebo (opposite arms) on Day 1, and one dose of ARCT-2303 on Day 29.

Cohort B (participants ≥65 years of age; approximately 480 participants)

Individuals will be randomly assigned to one of the three study groups:

- Group 1b (ARCT-2303/Fluad Quadrivalent, N=160): participants will receive one dose of ARCT-2303 and one dose of Fluad Quadrivalent (opposite arms) on Day 1, and one dose of placebo on Day 29.
- Group 2b (ARCT-2303, N=160): participants will receive one dose of ARCT-2303 and one dose of placebo (opposite arms) on Day 1, and one dose of Fluad Quadrivalent on Day 29.
- Group 3b (Fluad Quadrivalent, N=160): participants will receive one dose of Fluad Quadrivalent and one dose of placebo (opposite arms) on Day 1, and one dose of ARCT-2303 on Day 29.

In addition, all available serum samples from study participants of study ARCT-154-J01 who received a booster dose of candidate ARCT-154 vaccine according to the protocol, provided pre- and post-vaccination blood samples, and did not have evidence of SARS-CoV-2 infection and major protocol deviations, will be selected to use as a comparator. This set of samples will compose up to 385 individuals 18 years of age and above, who were included in the Per Protocol Set 1 (PPS-1). These samples will be tested in this study for assessment of coprimary study objectives 1 and 2.

Study design rationale: For the authorization of a modified COVID-19 vaccine against a particular SARS-CoV-2 variant of concern, US FDA Guidance "Emergency Use Authorization for Vaccines to Prevent COVID-19" (March 2022) requires a comparison of immune responses (assessed by neutralizing antibody) induced by the modified vaccine and the prototype vaccine. More specifically, for individuals who received the primary vaccination series and one booster dose with US-authorized COVID-19 vaccines, the study should be designed and adequately powered to demonstrate statistical superiority of the GMT elicited by the modified vaccine as compared to the prototype vaccine against the particular variant of concern. In addition, a second co-primary analysis should be a comparison of seroconversion rates against the particular variant of concern elicited by the modified and prototype vaccine. The study should be designed to demonstrate noninferiority of the seroconversion rate elicited by the modified vaccine as compared to the prototype vaccine, using a noninferiority margin of – 5% for seroconversion rate difference.

The use of the prototype vaccine (such as ARCT-154) as a comparator is not feasible based on the current recommendations for COVID-19 vaccine immunization, especially in participants of high risk of severe COVID-19. As such, individuals, who received the primary vaccination series and one booster dose of authorized mRNA vaccines previously and the second booster dose of ARCT-154 vaccine in study ARCT-154-J01 will be

used as a comparator. All participants who were included in the per protocol set 1 will be included in the comparator group.

The criteria for demonstration of superiority (GMT ratio) and noninferiority (SCR difference) are consistent with the US FDA guidance.

After the evaluation of the GMT and SCR responses of ARCT-2303, noninferiority of immune response after co-administration and standalone administration will be evaluated for ARCT-2303 and QIV vaccines.

To provide the opportunity to receive vaccination against COVID-19 and influenza diseases for all study participants and keep the blinded status of the study, a switchover vaccine dose or placebo will be administered on Day 29 (after the blood draw for serology testing).

Success criteria: The success criterion of the study is that the coprimary immunogenicity objectives 1 and 2 (Objectives Table) are demonstrated. Additionally, if coprimary objectives 1 and 2 are demonstrated, then the coprimary noninferiority objectives 3 and 4 (Objectives Table) will be tested; noninferiority of the immune response after co-administration and standalone administration of ARCT-2303 and Flucelvax Quadrivalent will be demonstrated if all 5 comparisons (four for influenza vaccine antigens and one for ARCT-2303) are successful. If all coprimary objectives 1, 2, 3, and 4 are demonstrated, secondary objectives will be tested by applying a sequential testing strategy as follows: if superiority of SCR (secondary immunogenicity objective 2; Objectives Table) is demonstrated, then super superiority of GMTs (secondary immunogenicity objective 1; Objectives Table) will be tested.

Randomization: An interactive response technology (IRT) will be used. Within each age cohort (A and B), participants will be randomly assigned at a 1:1:1 ratio into three study groups. Randomization will be stratified by COVID-19 vaccination history (such as, a total number of COVID-19 vaccine doses received and the composition of the last booster dose (mRNA original strain or mRNA bivalent)).

Blinding: Observer-blind.

Study visits: Four planned study site visits (Days 1, 29, 57, and 181).

Safety call: One planned safety call (Day 91).

Number of blood samples: Three blood draws (Days 1, 29, and 181) will be obtained from all participants.

Duration of the study/participant participation: Approximately 6 months for each participant.

Sample selection criteria for ARCT-154-J01 study participants:

All participants of study ARCT-154-J01 with available pre- and post-vaccination (Days 1 and 29) serum samples, who were allocated in the ARCT-154 group and were included in the PPS-1 for primary immunogenicity analysis (up to 385 participants) will be used as a control group.

Study Population: Healthy participants or individuals with pre-existing stable medical conditions ≥18 years of age who received primary vaccination series and at least one booster dose of the US-authorized mRNA COVID-19 vaccines, ≥5 months prior to enrollment.

Main Inclusion and Exclusion Criteria:

A full list of inclusion and exclusion criteria is provided in Section 5.1 and Section 5.2 of the protocol, respectively.

Main Inclusion Criteria:

- Individuals are male, female, or transgender adults ≥18 years of age.
- Healthy participants or participants with pre-existing stable medical conditions*.
- Participants or legally authorized representatives must freely provide documented informed consent prior to study procedures being performed.
- Individuals must agree to comply with all study visits and procedures (including blood tests, nasopharyngeal swabs, diary completion, and willingness to be available for planned telephone contacts and unscheduled clinic visits, if required).
- Individuals who received at least 3 doses (a 2-dose primary series and at least one booster dose) of the US-authorized mRNA COVID-19 vaccines. The last booster dose must be a US-authorized mRNA COVID-19 vaccine (original or bivalent), administered ≥5 months prior to enrollment.
- Individuals of childbearing potential must be willing to adhere to protocol contraceptive requirements and local regulations.

May not be used, divulged, published or otherwise disclosed without written consent of Arcturus Therapeutics, Inc.

* Pre-existing stable medical condition means a participant who: has a full capacity of daily activity and no major medication modification; has not undergone surgical or minimally invasive intervention or had any hospitalization/emergency room visit for the specific medical condition within 3 months prior to Day 1.
Main Exclusion Criteria:<br>• Individuals with acute medical illness or febrile illness, including temperature ≥100.4°F (≥38.0°C; measured by any method) within 3 days prior to enrollment.<br>• Individuals with laboratory-confirmed SARS-CoV-2 infection at Visit 1 (determined by positive Rapid Antigen Test or RT-PCR).<br>• Individuals who have received any investigational COVID-19 vaccines.<br>• Individuals who received any influenza vaccine within 6 months prior to enrollment.<br>• Individuals with a history of COVID-19 or virologically confirmed SARS-CoV-2 infection within the past 5 months or history of COVID-19 with ongoing sequelae.<br>• Individuals with a known history of severe hypersensitivity reactions, including anaphylaxis, or other significant adverse reactions to any vaccine, any components of mRNA vaccine, or influenza vaccine, including egg protein.<br>• Individuals with a history of myocarditis, pericarditis, myopericarditis, or cardiomyopathy.<br>• Individuals who have a positive pregnancy test at the Screening visit or who intend to become pregnant or breastfeed during the study.
**Study Vaccine and Control Vaccines Description:**<br>*Investigational COVID-19 vaccine:*<br>• ARCT-2303 vaccine contains 5 µg of mRNA encoding for spike glycoprotein of the Omicron XBB.1.5 subvariant of SARS-CoV-2 (embedded in lipid nanoparticles), 0.5-mL dose.<br>*Licensed influenza vaccines*:<br>• Flucelvax® Quadrivalent (Seqirus Pty Ltd.): a total of 60 µg hemagglutinin (HA) comprising 15 µg each of the World Health Organization (WHO)-recommended influenza strains against A/H1N1, A/H3N2, and two B strains, 0.5-mL dose.<br>• Fluad® Quadrivalent (Seqirus Pty Ltd.): a total of 60 µg hemagglutinin (HA) comprising 15 µg each of the World Health Organization (WHO)-recommended influenza strains against A/H1N1, A/H3N2, and two B strains, and MF59C.1 adjuvant, a squalene-based oil-in-water emulsion; 0.5-mL dose.<br>*Placebo*:<br>• 0.9% saline solution will be used as placebo, 0.5-mL dose.<br>**Route of administration:** Intramuscular (IM).

May not be used, divulged, published or otherwise disclosed without written consent of Arcturus Therapeutics, Inc.

Immunogenicity assessments:

Humoral immune responses against SARS-CoV-2 will be assessed in Groups 1a and 1b, Groups 2a and 2b, and in samples (Days 1 and 29) from ARCT-154 recipients, included in the PPS-1 in study ARCT-154-J01, using the following assays:

- Virus neutralizing assay (VNA, neutralizing antibodies against SARS-CoV-2) with Omicron XBB.1.5 subvariant;
- VNA (neutralizing antibodies against SARS-CoV-2 using various SARS-CoV-2 strains, including but not limited to ancestral SARS-CoV-2 strain and any prevalent variants)

Results from VNAs will be analyzed as:

- Geometric mean titer (GMT) on Days 1, 29 and 181;
- Geometric mean-fold rise (GMFR) as increases of the post-vaccination titer over the pre-vaccination titer;
- Seroconversion rate (SCR) on Day 29 as the percentage of participants in each arm with either:
    - A pre-vaccination titer below the lower limit of quantitation (LLOQ) and a post-vaccination titer ≥4xLLOQ; or
    - A pre-vaccination titer ≥LLOQ and a ≥4-fold increase in post-vaccination titer.
- Proportion of participants with antibody titer ≥LLOQ.

Humoral immune responses against QIV will be assessed in Groups 1a and 1b, and Groups 3a and 3b using the following assays:

- Hemagglutination Inhibition (HI) assay against influenza vaccine strains. In case of lack of agglutination for a specific strain using HI assay, immunogenicity for that strain will be assessed as measured by microneutralization (MN) assay;
- MN titers against influenza vaccine strains.

The results of HI and MN will be analyzed as:

- Geometric mean titer (GMT) on Days 1 and 29;
- Geometric mean-fold ratio (GMFR) as increases of the post-vaccination titer over the pre-vaccination titer;
- SCR on Day 29 as the percentage of participants in a group with either:
    - A pre-vaccination titer below the LLOQ for the respective assay and a post-vaccination titer ≥4xLLOQ; or
    - A pre-vaccination titer ≥ LLOQ and a ≥4-fold increase in post-vaccination titer.
- Proportion of participants with HI titer ≥1:10, 1:20, 1:40, 1:80, 1:160 and 1:320 on Days 1 and 29;
- Proportion of participants with MN titer ≥≥1:10, 1:20, 1:40, 1:80, 1:160 and 1:320 on Days 1 and 29.

CONFIDENTIAL

May not be used, divulged, published or otherwise disclosed without written consent of Arcturus Therapeutics, Inc.

**Safety assessments:**<br>• All participants will be monitored for 30 minutes after each study injection for any immediate post-vaccination adverse events (AEs) with appropriate medical treatment readily available in case it is needed.<br>• Solicited local reactions and systemic AEs will be recorded daily within 7 days after each study vaccination:<br>o Local reactions: pain, erythema and swelling at the injection site.<br>o Systemic AEs: fatigue, headache, myalgia, arthralgia, nausea, dizziness, chills, and fever.<br>On Day 1, when ARCT-2303 will be co-administered with the influenza vaccine, participants will be instructed to record local reactogenicity for both arms separately.<br>Specifically, participants in:<br>Group 1a and 1b will receive: ARCT-2303 on the left arm and influenza vaccine on the right arm<br>Group 2a and 2b will receive: ARCT-2303 on the left arm and placebo on the right arm<br>Group 3a and 3b will receive: placebo on the left arm and influenza vaccine on the right arm<br>• Any unsolicited AEs will be collected within 28 days after each study vaccination (from Day 1 to Day 29 and from Day 29 to Day 57).<br>• Any medically attended AEs (MAAEs), serious AEs (SAEs), AEs of special interest (AESIs), and AEs leading to early study termination and any pregnancies will be reported during the entire study period. This data will be captured by interviewing the participant during the site visits and phone calls and by reviewing available medical records.<br>• Any medically diagnosed episodes of SARS-CoV-2 infection will be reported as MAAEs; no active surveillance for SARS-CoV-2 infection is planned in this study.
**Statistical considerations:**
**Main analysis population:**<br>Per Protocol Analysis Set (PP)<br>Includes all participants who received the correctly assigned study vaccines and who have no protocol deviations impacting the analysis of immunogenicity data for the time period summarized as evaluated by the blinded Sponsor Medical Monitor.<br>**Sample Size:**<br>A total sample size of approximately 1680 enrolled participants (1200 participants 18 to 64 years of age, and 480 participants ≥65 years of age), and a sample size up to 385 participants of study ARCT-154-J01 is proposed for the following study objectives:<br>1) the coprimary objective 1 of superiority of the ARCT-2303 vaccine (Groups 2a and 2b) compared to a booster dose of ARCT-154 for Omicron XBB.1.5 subvariant<br>Superiority of $GMT_{ARCT\text{-}2303\ (booster)}$ to $GMT_{ARCT\text{-}154\ (booster)}$<br>The superiority margin for Omicron XBB.1.5 strain is defined as 1.0 for the GMTs ratio; thus the lower limit of the 2-sided 95% CI for GMT ratios (ARCT-2303/ARCT-154) is higher than 1.0.<br>Assumptions for the above calculations for the coprimary objective 1) are:<br>The GMT ratio between the ARCT-2303 (Day 29) and ARCT-154 (booster dose, Day 29) groups is expected to be >1.5, and the common standard deviation is expected to be 0.60 in the $\log_{10}$ scale.<br>2) coprimary objective 2 of the noninferiority of the ARCT-2303 vaccine (Groups 2a and 2b) compared to a booster dose of ARCT-154 for the Omicron XBB.1.5 subvariant, in terms of SCR, is also considered:<br>Noninferiority (NI) of $SCR_{ARCT\text{-}2303\ (booster)}$ versus $SCR_{ARCT\text{-}154\ (booster)}$

The noninferiority margin for the Omicron XBB.1.5 subvariant is defined as -5% for the SCRs difference, thus the lower limit of the 2-sided 95% CI for SCR difference (ARCT-2303 minus ARCT-154) is higher than - 5% (i.e., minus 5%).

Assumptions for the above calculations for the coprimary objective 2) are:

The SCR difference between the ARCT-2303 (Day 29) and ARCT-154 (booster, Day 29) groups is expected to be at least 10%, and the SCR is equal to 50% in the ARCT-154 vaccine group.

A sample size of up to 385 participants of study ARCT-154-J01, who received a booster dose of ARCT-154 vaccine, provided evaluable pre- and post-vaccination blood samples, and did not have SARS-CoV-2 infection and protocol deviations that impact on immunogenicity assessment, and up to 560 participants in the ARCT-2303 group (Groups 2a and 2b) provides approximately 99% power (evaluable number of participants n=350 and n=500 in the two groups if there is dropout of approximately 10%) to demonstrate each of the 2 coprimary objectives; therefore, there is at least 97% overall power to demonstrate the two coprimary objectives, 1 and 2.

If coprimary objectives 1 and 2 are met, then the second step will be assessing the noninferiority of immune response after co-administration and standalone administration of ARCT-2303 and Flucelvax Quadrivalent vaccines.

The noninferiority objectives for the co-administration are:

3) vaccination with Flucelvax Quadrivalent, when given concomitantly with ARCT-2303, compared to that of Flucelvax Quadrivalent, when given standalone

NI of $GMT_{QIV+\ ARCT\text{-}2303}$ to $GMT_{QIV\ +\ Placebo}$ (Group 1a vs Group 3a)

4) vaccination with ARCT-2303, when given concomitantly with Flucelvax Quadrivalent, compared to that of ARCT-2303, when given standalone

NI of $GMT_{ARCT\text{-}2303\ +\ QIV}$ to $GMT_{ARCT\text{-}2303+placebo}$ (Group 1a vs Group 2a)

The noninferiority margin is defined as 0.67 for the GMTs ratios.

Assumptions for the above calculations are:

the GMT ratios between the

a-Flucelvax Quadrivalent, when given concomitantly with ARCT-2303, compared to Flucelvax Quadrivalent, when given standalone,

b-ARCT-2303, when given concomitantly with Flucelvax Quadrivalent, compared to ARCT-2303, when given standalone,

is expected to be 1.0 and the common standard deviation is expected to be 0.60 in the $\log_{10}$ scale.

A sample size of 400 participants per group (Groups 1a, 2a, and 3a) provides 97.5% power (n=360 per group if there is dropout is approximately 10%) to demonstrate noninferiority for each one of the 5 strains (4 strains for influenza vaccine and 1 strain for ARCT-2303); therefore, there is at least 88% overall power to demonstrate all of the noninferiority objectives. The overall power to demonstrate all 4 coprimary objectives is at least 85%.

CONFIDENTIAL

May not be used, divulged, published or otherwise disclosed without written consent of Arcturus Therapeutics, Inc.

Sequence of the Analysis:

The statistical analyses for the coprimary immunogenicity objectives (1, 2, 3, and 4) will be performed and tested in 2 steps.

The first step will be testing coprimary objectives 1 and 2:

1 – superiority for the Omicron XBB.1.5 subvariant on Day 29 for GMT ratio.

2 – noninferiority for the Omicron XBB.1.5 subvariant on Day 29 for the difference of SCR.

The success criterion of the study is that the coprimary objectives 1 and 2 are demonstrated. If the coprimary objectives 1 and 2 (based on GMT ratio and difference of SCRs) are both met, then the testing will continue to the second step sequentially.

The second step will be testing coprimary objectives 3 and 4 by assessing the noninferiority of immune response after co-administration and standalone administration of ARCT-2303 and Flucelvax Quadrivalent.

The success criterion for the co-administration part of the study is that the coprimary objectives 3 (noninferiority for all 4 influenza strains) and 4 (noninferiority for XBB.1.5 variant) are demonstrated.

If all coprimary objectives are met, an additional step of sequential testing of the secondary objective 2 (superiority based on SCR) followed by the testing of the secondary objective 1 ('super superiority' for GMT ratio) will be performed.

No adjustment for multiplicity is planned for this study due to the hierarchical testing strategy.

Statistical Hypotheses and Hypothesis Testing:

Step 1:

Coprimary immunogenicity objectives 1 and 2, evaluating the ARCT-2303 as compared to a booster dose of ARCT-154 for Omicron XBB.1.5 subvariant.

1) For the coprimary immunogenicity objective 1 evaluating the superiority of ARCT-2303 as compared to ARCT-154 in terms of the neutralizing immune response against Omicron XBB.1.5 subvariant, the following null hypothesis will be applied and evaluated in the PP population:

H_{01}: Day 29 GMT ARCT-2303 $_{\text{booster dose}}$ /Day 29 GMT ARCT-154$_{\text{booster}}$ ≤ 1.0

2) For the coprimary immunogenicity objective 2 evaluating the noninferiority of ARCT-2303 as compared to ARCT-154 in terms of seroconversion rate against Omicron XBB.1.5 subvariant, the following null hypothesis will be applied and evaluated in the PP population:

H_{02}: Day 29 SCR ARCT-2303 $_{\text{booster dose}}$ minus Day 29 SCR ARCT-154$_{\text{booster}}$ $\leq$ -5%

Two-sided α= 0.05 will be used for all tests.

Step 2:

If objectives in step 1 are met, then the noninferiority of immune response after co-administration and standalone administration of ARCT-2303 and Flucelvax Quadrivalent will be compared (coprimary objectives 3 and 4), and the following null hypotheses will be applied and evaluated in the PP population:

H_{03}: Day 29 GMT QIV $_{\text{QIV+ARCT-2303}}$ /Day 29 GMT QIV $_{\text{QIV+Placebo}}$ ≤ 0.67 (Group 1a vs 3a)

H_{04}: Day 29 GMT ARCT-2303 $_{\text{QIV+ARCT-2303}}$ /Day 29 GMT ARCT-2303$_{\text{ARCT-2303+Placebo}}$ ≤ 0.67 (Group 1a vs 2a)

No adjustment for multiplicity is planned as H_{03} and H_{04}. All 5 null hypotheses; 4 strains for influenza vaccine and 1 strain for ARCT-2303 must be rejected to declare noninferiority of immune response.

Step 3:

If coprimary objectives 1, 2, 3 and 4 are met, then secondary objectives 2 (superiority of SCR) and 1 ('super superiority' of GMTs) of the ARCT-2303 vaccine, when given as a booster dose compared to a booster dose of ARCT-154 (in study ARCT-154-J01), will be tested in a sequential manner as follows:

H_{05}: Day 29 SCR ARCT-2303 $_{\text{booster dose}}$ minus Day 29 SCR ARCT-154$_{\text{booster}}$ ≤ 0%

H_{06}: Day 29 GMT ARCT-2303 $_{\text{booster dose}}$ /Day 29 GMT ARCT-154$_{\text{booster}}$ ≤ 1.5

The ratio of GMTs and associated two-sided 95% confidence interval (CI) between Day 29 ARCT-2303 and Day 29 ARCT-154$_{\text{booster}}$ will be calculated as the exponentiation of the difference of the two means of the logarithmically transformed assay results of the two groups and associated two-sided 95% CI. Analysis of covariance (ANCOVA) will be used to calculate the logarithmical mean and confidence interval with covariate and factor adjustment (further details will be described in the statistical analysis plan). Analyses will be repeated by age cohort.

Superiority of ARCT-2303 to ARCT-154 in terms of neutralizing antibodies titers against Omicron XBB.1.5 subvariant will be demonstrated if the lower limit of the 2-sided 95% CI for GMT ratios (ARCT-2303/ARCT-154) is higher than the specified limit (1.0).

Noninferiority of ARCT-2303 to ARCT-154 in terms of neutralizing antibodies seroconversion rate (SCR) against Omicron XBB.1.5 subvariant will be demonstrated if the lower limit of the two-sided 95% CI for SCR difference (ARCT-2303 minus ARCT-154) is higher than the specified limit (-5%).

Similarly, Day 29 ratio of GMTs and associated two-sided 95% confidence interval for ARCT-2303 and Flucelvax Quadrivalent (co-administration compared to standalone) will be calculated as the exponentiation of the difference of the two means of the logarithmically transformed assay results of the two groups and associated two-sided 95% CI. Noninferiority of ARCT-2303 and Flucelvax Quadrivalent (co-administration versus standalone) will be demonstrated if the lower limit of the 2-sided 95% CI for GMT ratios (Group 1a vs Group 3a and Group 1a vs Group 2a) is higher than the specified limit (0.67).

Super superiority of ARCT-2303 to ARCT-154 in terms of neutralizing antibodies GMTs against Omicron XBB.1.5 subvariant will be demonstrated (secondary objective 1) if the lower limit of the 2-sided 95% CI for GMT ratios (ARCT-2303/ARCT-154) is higher than the specified limit (1.5).

Superiority of ARCT-2303 to ARCT-154 in terms of neutralizing antibodies SCR against Omicron XBB.1.5 subvariant will be demonstrated (secondary objective 2) if the lower limit of the 2-sided 95% CI for difference of SCRs (ARCT-2303 minus ARCT-154) is higher than the specified limit (0%). Super superiority will be demonstrated if the lower limit of the 2-sided 95% CI for difference of SCRs (ARCT-2303 minus ARCT-154) is higher than the specified limit (10%).

There is no formal hypothesis testing for evaluating the co-administration of ARCT-2303 and Fluad Quadrivalent. The results of the analysis will be presented descriptively.

There is no formal hypothesis testing for the evaluation of the safety objective. Safety will be summarized descriptively within the Safety Analysis Set population.

CONFIDENTIAL

May not be used, divulged, published or otherwise disclosed without written consent of Arcturus Therapeutics, Inc.

Interim Analyses and Analysis Timing:

No interim analysis is planned for the study.

The results of this study will be released sequentially in two separate final analyses.

The first final analysis will include all immunogenicity data (up to Day 29), reactogenicity data (7 days post-vaccination), unsolicited AEs (28 days post-vaccination), and safety data collected from Day 1 to Day 57 and associated primary and secondary objectives. This analysis will be conducted on cleaned and frozen data. The results of this analysis will be presented in the initial clinical study report (CSR). Immunogenicity and safety data will be reported on a group level only. No individual listings will be generated. Access to participant-level information about study groups will be restricted to specified personnel involved in the statistical analysis. Site staff, CROs, and sponsor representatives remain blinded at the participant level.

The second final analysis of immunogenicity and safety data collected from Day 57 to the study end will be performed as soon as all data are available. The results of this analysis will be presented in the final CSR. All individual data listings with information on the participant study group will be presented in the final CSR.

Data Safety Monitoring Board and Central Cardiac Adjudication Committee:

The study will include the use of an independent unblinded Data Safety Monitoring Board (DSMB) and a Central Cardiac Adjudication Committee.

The DSMB will meet periodically to review study data and provide recommendations to the Sponsor during the entire study period. The members of the DSMB serve in an individual capacity and provide their expertise, including recommendations regarding the continuation, modification, or termination of the study. The DSMB may review cumulative study data to evaluate the safety, study conduct, scientific validity, and data integrity of the study. Further details are provided in the DSMB charter.

A central cardiac adjudication committee may meet to evaluate ECGs and assess any potential myocarditis/pericarditis cases. Further details are provided in the cardiac adjudication committee charter.

ARCT-2303-01 Version 2.0 (21 February 2024)

1.1 Study Design

The study design for ARCT-2303-01 is shown in Figure 1.

Figure 1 Study Design for ARCT-2303-01

Cohort A (individuals 18 to 64 years of age)

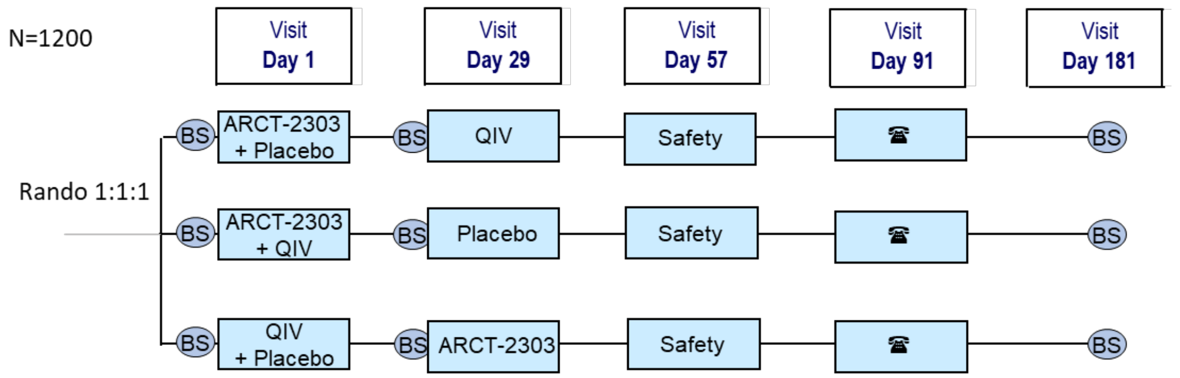

Abbreviations: QIV, Flucelvax Quadrivalent vaccine; BS, blood sample; rando, randomization.

Cohort B (individuals ≥65 years of age)

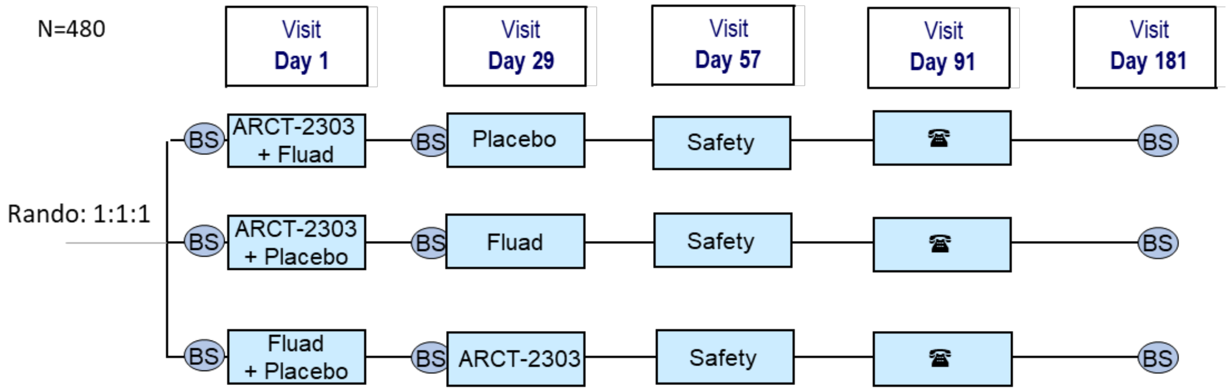

Abbreviations: Fluad, Fluad Quadrivalent vaccine; BS, blood sample; rando, randomization.

A set of participants from study ARCT-154-J01 (second booster dose)

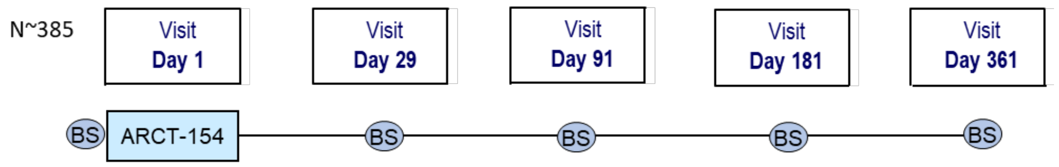

Abbreviation: BS, blood sample.

1.2 Schedule of Assessments

The schedule of assessments (SoA) for ARCT-2303-01 is shown in Table 1.

Table 1 Schedule of Assessments

Visit Type:	Screening and Vacc1	Follow-up Visit 1 and Vacc2	Follow-up Visit 2	Safety call	Final Visit (EOS)	Unscheduled Visit	Early Termination (ET) Visit
	Clinic[a]	Clinic[a]	Clinic[a]	TC	Clinic[a]	Clinic[a, c]	Clinic[a]
**Study Day:**	**1 [b]**	**29**	**57**	**91**	**181**	**N/A**	**N/A**
**Visit Window (days):**	**-7**	**±3**	**±3**	**±7**	**±7**	**N/A**	**N/A**
Informed consent	X[b]						
Weight/height	X[b]						
Medical history and demographics	X[b]						
Physical examination [d]	X[b]	X	X		X	X	X
Vital signs, and body temperature [e]	X[b]	X	X		X	X	X
Pregnancy test [f]	X[b]	X					
Testing for COVID-19 [g]	X[b]	X				X[g]	
Review of inclusion/exclusion criteria	X[b]						
Contraindication for second vaccination [h]		X					
Randomization	X						
Blood sampling for immunogenicity and safety testing [i]	X	X			X	(X)	(X)
12-lead electrocardiogram [j]	X					X	
Study vaccine administration [k]	X	X					
Post-vaccination observation [e, l]	X	X					
Dispense eDiary and training	X						
Diary compliance check [m]	X	X	X				
Adverse events [n]	X[b]	X	X	X	X	X	X
Concomitant medication/vaccines collection	X[b]	X	X	X	X	X	X

Abbreviations: AE, adverse event; AESI, adverse event of special interest; COVID-19, coronavirus disease 2019; ECG, electrocardiogram; eDiary, electronic diary; ET, early termination; MAAE, medically attended adverse event; N/A, not applicable; RT-PCR, real-time polymerase chain reaction; SAE, serious adverse event; SARS-CoV-2, severe acute respiratory syndrome coronavirus 2; TC, telephone call; Vacc, vaccination.

a Visits will be performed in person unless the participant is otherwise directed to be seen at a hospital or to remain at home (if telemedicine/telephone visits are the only feasible option).

Table 1 Schedule of Assessments

b Required Screening procedure. Screening procedures should be performed on the Day 1 vaccination visit, if feasible, and a maximum of 7 days prior to randomization. All Screening procedures must be performed prior to the determination of eligibility and enrollment of the participant in the trial. If all Screening procedures are performed before Day 1, the following Screening assessments should be repeated before dosing on Day 1: physical examination, vital signs, body temperature, pregnancy testing, and testing for COVID-19.

c Unscheduled Visits include visits for possible COVID-19, possible cardiac events, or evaluation of any adverse event of concern. These visits may be performed in the clinic, by home visit, by hospital visit (if allowed by local policy), or via telemedicine/telephone visit. Required procedures at these visits include AE and concomitant medication collection. Should the visit occur in person, the visit should also include an evaluation of vital signs, body temperature, and symptom-directed physical examination. For participants evaluated for SARS-CoV-2, samples may be collected for SARS-CoV-2 RT-PCR testing. For participants evaluated for cardiac symptoms, a 12-lead electrocardiogram and targeted safety laboratory assessments should be performed. Additional safety phone calls may be performed for the evaluation of adverse events.

d A complete physical examination will be performed at Screening and Final Visit (or ET); symptom-directed examination (if any symptoms) will be performed at other time points as indicated to assess changes from Screening.

e Blood pressure, heart rate, respiratory rate, and body temperature (by any method) will be measured. On Day 1 and Day 29, these will be assessed prior to and following study vaccination. Vital signs should be measured in a seated position after the participant has rested comfortably for at least 5 minutes. Post vaccination, vital signs should be measured within the 30-minute observation period prior to the participant leaving the site.

f Pregnancy testing will be performed on women who are not surgically sterile or postmenopausal. Urine or serum pregnancy test can be used at the discretion of the Investigator or as per local requirements. Pregnancy testing must be performed and evaluated within the same day prior to study vaccine administration.

g A nasal swab will be collected for validated SARS-CoV-2 rapid antigen testing at Screening to confirm eligibility of participants for recruitment. Prior to each study vaccination, rapid antigen test results must be available for the determination of eligibility. Additional SARS-CoV-2 RT-PCR testing may be performed at planned or unscheduled visits in any participant who is being evaluated for risk of possible COVID-19 infection.

h For contraindications for the second vaccination and criteria for vaccination delay, refer to Section 5.3.

i Blood samples will be taken for neutralizing antibody testing before vaccine administration on Days 1 and 29 and on Day 181 for testing of immune responses to SARS-CoV-2 and future exploratory testing, including new emerging SARS-CoV-2 variants. In addition, blood samples may be used for safety testing in case of suspected myocarditis or other potential safety concerns. Blood samples for cardiac enzymes will be collected on Day 1 prior to vaccination (baseline) for all participants. The testing of baseline and post-vaccination samples for cardiac enzymes might be performed based on the investigator's or Sponsor's discretion. In case of early termination from the study due to safety reasons, a blood sample should be collected at the early termination visit. Collection of blood samples at unscheduled visits is at the investigator's discretion for evaluation of safety.

j A baseline 12-lead ECG at recruitment will be performed on all study participants to identify potential asymptomatic myocarditis and pericarditis cases. In case of suspected myocarditis or pericarditis cases during the study period, detailed evaluation, including ECG, cardiac enzyme testing, and other relevant data should be evaluated. Additional ECG evaluation at unscheduled visits is at the investigator's discretion. Baseline ECG might be performed within 7 days prior to subject recruitment in the study.

CONFIDENTIAL

May not be used, divulged, published or otherwise disclosed without written consent of Arcturus Therapeutics, Inc.

Table 1 Schedule of Assessments

k Study vaccines will be administered by intramuscular injection into the lateral area of the deltoid muscle of the opposite arms on Day 1. On Day 29, the study vaccine will be administered into the lateral area of the deltoid muscle of a non-dominant arm. Study vaccine will be administered in an observer-blinded fashion.

l Vaccinated participants will be observed at the site for at least 30 minutes following vaccine administration and until clinically stable.

m Compliance checks for eDiary completion will be performed on Day 2 (±1) and Day 5 (±1) post-vaccination. Compliance checks will be documented in the source. The site will perform a check to verify that the participant has completed the eDiary. Participants will be contacted by telephone if the eDiary is incomplete. The review of eDiary will be performed on Day 29 and Day 57 visits.

n Review of AEs will include surveillance for solicited and unsolicited AEs, SAEs, MAAEs, AESIs, and AEs leading to discontinuation/withdrawal; data will be gathered by eDiary, interview at clinic visits, and telephone contacts. Solicited AEs will be collected by eDiary for 7 days after each study vaccination for all participants. All unsolicited AEs will be collected for 28 days after each vaccination. SAEs, MAAEs, AESIs, and AEs leading to discontinuation/withdrawal will be collected at all time points after first vaccination.

CONFIDENTIAL

May not be used, divulged, published or otherwise disclosed without written consent of Arcturus Therapeutics, Inc.

2 INTRODUCTION

The purpose of this study is to demonstrate that immune response induced by a booster dose of the ARCT-2303 vaccine (monovalent COVID-19 vaccine with an XBB.1.5-sublineage of the Omicron variant) is superior to those in individuals who received a booster dose of ARCT-154 as measured by GMTs of neutralizing antibodies against Omicron XBB.1.5 subvariant, and noninferior as measured by neutralizing antibodies SCR against Omicron XBB.1.5 subvariant.

In addition, the study intends to demonstrate that the immune responses against the ARCT-2303 vaccine and the quadrivalent influenza vaccine, when administered concomitantly and separately, are noninferior. These objectives will be demonstrated in individuals 18 to 64 years of age who receive Flucelvax Quadrivalent (Seqirus). Additional descriptive analysis of co-administration will be performed in participants ≥65 years of age who receive adjuvanted influenza vaccine (Fluad Quadrivalent, Seqirus).

2.1 Background

Severe acute respiratory syndrome coronavirus 2 (SARS-CoV-2) infection leads to a cluster of respiratory illnesses, collectively called coronavirus disease 2019 (COVID-19), similar to those caused by SARS-CoV. COVID-19 has been associated with a high transmission rate, and severe cases require admission to hospital intensive care units (ICUs) with the need for mechanical ventilation and associated high mortality. As of March 2023, over 760 million cases of COVID-19 have been confirmed worldwide and over 6.8 million people have died (https://covid19.who.int/).

Several COVID-19 vaccines, based on the ancestral strain of the SARS-CoV-2 virus, were developed, authorized, and broadly used to prevent the spread of the COVID-19 pandemic and reduce morbidity and mortality associated with COVID-19.

Arcturus Therapeutics, Inc. (Arcturus) developed a candidate ARCT-154 vaccine for prophylaxis of COVID-19. The vaccine is composed of the lipid nanoparticle (LNP) formulation of an mRNA replicon(s) based upon Venezuelan equine encephalitis virus (VEEV) in which RNA encoding for the VEEV's structural proteins has been replaced with RNA encoding for the SARS-CoV-2 full-length spike (S) glycoprotein.

ARCT-154 vaccine, containing 5 µg of mRNA encoding ancestral SARS-CoV-2 strain with a D614G mutation, was assessed in a randomized, controlled Phase 1/2/3 clinical study (ARCT-154-01). The study evaluated the safety, efficacy, and immunogenicity of the candidate vaccine, administered as a primary vaccination series in adults ≥18 years of age. As part of that study execution, in Phase 3b, over 12,700 younger adults (18 years of age to <60 years) and over 2,600 older adults (≥60 years of age) have received at least one dose of the ARCT-154 vaccine during the primary vaccination. ARCT-154 demonstrated 56.6% efficacy (95% CI: 48.7% - 63.3%) against COVID-19 disease of any severity and 95.3% efficacy (95% CI: 80.5% - 98.9%) against severe COVID-19. In the immunogenicity analysis at Day 57, the study found that 95.9% (95%CI: 93.4%-97.6%) of the ARCT-154 recipients achieved neutralizing antibody seroconversion after 2 doses as measured by a microneutralization assay. In total, 16,393 study participants received at least one dose of the ARCT-154 vaccine, 15,139 participants received a 2-dose vaccination series, and 483 participants received three

doses of the vaccine. ARCT-154 had an acceptable safety profile with no major safety concerns raised from available data.

A single dose of the ARCT-154 vaccine induced a robust booster response against the ancestral strain and multiple variants of concerns in individuals who previously received primary vaccination with authorized mRNA vaccines.

To extend the protection against new emergent variants of SARS-CoV-2 and to address the recent recommendations of public health agencies for booster vaccine composition (Updated COVID-19), Arcturus developed ARCT-2303 vaccine, which contains 5 µg of mRNA encoding for Omicron XBB.1.5 subvariant spike glycoprotein.

The purpose of this study is to demonstrate that immune response induced by a booster dose of the ARCT-2303 vaccine is superior to those in individuals who received a booster dose of ARCT-154 in study ARCT-154-J01, as measured by GMTs of neutralizing antibodies against Omicron XBB.1.5 subvariant, and noninferior as measured by neutralizing antibodies SCR against Omicron XBB.1.5. In study ARCT-154-J01, ARCT-154 candidate vaccine with ancestral SARS-CoV-2 strain induces immune response that was at least noninferior to those of authorized COVID-19 mRNA vaccine (Pfizer's BNT162b2).

As both COVID-19 and influenza vaccines are recommended to the general population in many countries, the present study is also designed to ensure that neither vaccine interferes with the safety or immunogenicity of the other vaccine when administered concomitantly in adults.

Flucelvax Quadrivalent is one of the broadly used seasonal influenza vaccines and the only influenza vaccine in use in the US for which the vaccine viruses are grown in cell culture. This vaccine is licensed for use in individuals 6 months and older.

For individuals 65 years of age and above, there are three influenza vaccines preferentially recommended in the US. They include Fluad Quadrivalent, a standard-dose, inactivated influenza vaccine that contains MF59 (squalene-based oil-in-water adjuvant). An adjuvant is an ingredient added to a vaccine that helps create a stronger immune response to vaccination. Fluad Quadrivalent is approved for use among people 65 years and older.

The Southern Hemisphere 2024 season vaccine composition for both influenza vaccines will be used in the study.

Recent studies (Domnich et al., 2022) indicate that there are no specific safety concerns raised over administration of mRNA COVID-19 vaccines (such as Pfizer's BNT162b2) in adults alongside standard dose inactivated influenza vaccines, including those with MF59 adjuvant. Concomitant vaccination with both COVID-19 and influenza vaccines should reduce the burden on the healthcare services for vaccine delivery, allowing for timely vaccine administration and protection from COVID-19 and influenza for those in need (Lazarus et al., 2021).

The study intends to demonstrate that the immune responses against the ARCT-2303 vaccine and the quadrivalent influenza vaccine, when administered concomitantly and separately, are noninferior. These objectives will be demonstrated in individuals 18 to 64 years of age who receive Flucelvax Quadrivalent. Additional descriptive analysis of co-administration will be performed in participants ≥65 years of age who receive adjuvanted influenza vaccine (Fluad Quadrivalent).

May not be used, divulged, published or otherwise disclosed without written consent of Arcturus Therapeutics, Inc.

2.1.1 Nonclinical Studies

Preclinical studies conducted with previous COVID-19 mRNA candidate vaccines (ARCT-021 and ARCT-154) support the clinical development of ARCT-2303, given the similarities of the vaccines. ARCT-154 has been evaluated in nonclinical pharmacology studies using murine and non-human primate (NHP) models demonstrating robust antibody responses following vaccination, including increased antibody responses to heterologous vaccination for differing SARS-CoV-2 strains. Vaccination with ARCT-021 induced significant immune protection in multiple SARS-CoV-2 challenge studies in animals (mice and NHPs), with no signs of vaccine-enhanced disease (VED).

Two nonclinical GLP repeat-dose toxicology studies in rabbits were conducted for ARCT-021. Animals receiving ARCT-021 showed transient findings that resolved by 2 to 4 days post-injection and did not worsen following subsequent dosing. A fertility, embryofetal (EFD), and postnatal (PND) study conducted with ARCT-021 in female rabbits did not produce any developmental toxicity and resulted in maternal body weight effects that recovered after cessation of dosing.

A GLP repeat-dose toxicology study in rabbits was conducted for ARCT-154. ARCT-154 induced transient findings and did not identify any unexpected safety findings. All findings were consistent with the expected pharmacological immune response to the vaccine.

The investigator's brochure (IB) includes further details on nonclinical studies.

2.1.2 Clinical Studies

The current clinical development program incorporates the evaluation of three investigational mRNA SARS-CoV-2 vaccines, developed by Arcturus: ARCT-021, ARCT-154, ARCT-165.

ARCT-021 has been administered to over 700 participants in clinical trials and has been generally well tolerated and immunogenic in studies enrolling healthy younger and older adults.

ARCT-154-01 is a multicenter, Phase 1/2/3 randomized, controlled, observer-blind study designed to evaluate the safety, immunogenicity, and efficacy of ARCT-154 in participants 18 years of age and older in Vietnam. In Phase 3b of the ARCT-154-01 study, a total of 16,120 participants were randomly assigned to receive 2 doses of ARCT-154 or placebo with 8,056 exposed participants in the ARCT-154 group and 8,044 exposed participants in the placebo group.

For participants without evidence of SARS-CoV-2 infection prior to 7 days after Dose 2, overall vaccine efficacy was 56.6% (95% confidence interval [CI]): 48.7% to 63.3%), which met the prespecified success criterion. Vaccine efficacy against severe protocol-confirmed COVID-19 occurring at least 7 days after Dose 2 was 95.3% (95% CI: 80.5% – 98.9%).

In the immunogenicity analysis on Day 57, the study found that 95.9% (95%CI: 93.4 - 97.6) of the ARCT-154 recipients achieved seroconversion after 2 doses as measured by neutralizing antibodies using a microneutralization assay. ARCT-154 vaccine induced broad neutralization of SARS-CoV-2 variants of concern; the seroconversion rates against the most variants at Day 57 (28 days after Dose 2) were above 90% in the ARCT-154 group. GMFR from baseline (Day 1) for most variants at Day 57 was above 10-fold in the ARCT-154 group.

CONFIDENTIAL

May not be used, divulged, published or otherwise disclosed without written consent of Arcturus Therapeutics, Inc.

ARCT-154-01 has an acceptable safety profile with no major safety concerns in the adult study population:

- Solicited local adverse reactions were reported by 52.4% and 15.3% of Phase 3b participants in ARCT-154 and placebo groups, respectively, within 7 days after the first and second vaccinations. Systemic solicited AEs were reported by 60.6% and 40.9% of participants, respectively.
- Any unsolicited AEs within 28 days after the first vaccination were reported by 14.0% of participants in ARCT-154 group and 13.7% of participants in the placebo group. After the second vaccination, unsolicited AEs were reported by 13.9% and 15.9% of participants in the ARCT-154 and placebo groups, respectively. The majority of these events were not related to study vaccine.
- Serious AEs (SAEs) were reported by 118/8059 (1.5%) ARCT-154 recipients and 201/8041 (2.5%) placebo recipients in the Phase 3b stage within 92 days after the first vaccination (prior to switchover). Severe SAEs were reported by 20/8059 (0.2%) and 73/8041 (0.9%) of participants respectively. Related SAEs were reported by 10/8059 (0.1%) and 5/8041 (0.1%) participants in the ARCT-154 and placebo groups, respectively.
- From Day 92 (switchover) to Day 210, 88/7458 (1.2%) participants who received placebo after switchover, and 91/7353 (1.2%) participants who received ARCT-154 after switchover, reported at least one SAE. Severe SAEs were reported by 34/7458 (0.5%) and 23/7353 (0.3%) of participants, respectively. One related SAE (anaphylactic reaction that does not meet Brighton Collaboration criteria) was reported after switchover vaccination with ARCT-154 vaccine.
- No cases of myocarditis or pericarditis following administration of ARCT-154 or placebo were reported up to Day 210 post-vaccination.

In a Phase 1/2 clinical study ARCT-165-01, a booster dose of ARCT-154 induced a robust immune response in individuals who previously received the primary vaccination with a COVID-19 mRNA vaccine. Geometric fold increases of neutralizing antibodies (Day 29/pre-booster) against ancestral SARS-CoV-2 strain, and variants Beta, Delta, Omicron BA.1, BA.2, BA.4/5, BQ.1.1 and XBB.1.5 were 71, 87, 62, 49, 47, 33, 13, and 3, respectively. Neutralizing antibodies against various SARS-CoV-2 variants persist for at least 12 months post-booster dose.

In a Phase 3 clinical study in Japan (ARCT-154-J01), a booster dose of ARCT-154 elicited a noninferior immune response against Wuhan-Hu1 strain and superior immune response against Omicron BA.4/5 subvariant compared with the authorized mRNA vaccine (Comirnaty, Pfizer). The reactogenicity and safety profiles of both vaccines were similar, with no safety concerns raised.

Overall, clinical data for parent mRNA COVID-19 vaccines (ARCT-021, ARCT-154 and ARCT-165) support the favorable benefit-risk profile in adult participants.

Additional clinical data are presented in the latest version of the IB.

CONFIDENTIAL

May not be used, divulged, published or otherwise disclosed without written consent of Arcturus Therapeutics, Inc.

2.2 Study Rationale

Several COVID-19 vaccines were authorized and globally used for primary vaccination against COVID-19 and, recently, to boost the immune response after the initial vaccination series.

Arcturus developed a candidate mRNA vaccine, ARCT-154, for prophylaxis of COVID-19. This vaccine is composed of a lipid nanoparticle (LNP) formulation of an mRNA replicon(s) based upon Venezuelan equine encephalitis virus (VEEV) in which RNA encoding for the VEEV's structural proteins has been replaced with RNA encoding for the SARS-CoV-2 full-length spike (S) glycoprotein. ARCT-154 vaccine includes 5 µg of mRNA encoding the ancestral SARS-CoV-2 strain with a D614G mutation. The vaccine was assessed in the phase 1/2/3 clinical study ARCT-154-01 and demonstrated a positive benefit/risk ratio evaluated in more than 17,000 healthy adult participants and individuals with stable pre-existing chronic medical conditions in Vietnam.

Following recent recommendations of the US CDC and US FDA, Arcturus developed a modified monovalent COVID-19 vaccine with an XBB.1.5-sublineage of the Omicron variant (ARCT-2303) that includes 5 µg of mRNA encoding for the XBB.1.5 variant spike glycoprotein (embedded in lipid nanoparticles).

For the authorization of a modified COVID-19 vaccine against a particular SARS-CoV-2 variant of concern, US FDA Guidance 'Emergency Use Authorization for Vaccines to Prevent COVID-19' (March 2022) requires a comparison of immune responses (assessed by neutralizing antibody) induced by the modified vaccine and the prototype vaccine. More specifically, for individuals who received the primary vaccination series and one booster dose with US-authorized COVID-19 vaccines, the study should be designed and adequately powered to demonstrate statistical superiority of the GMT elicited by the modified vaccine as compared to the prototype vaccine against the particular variant of concern. In addition, a second co-primary analysis should be to demonstrate noninferiority of the seroconversion rate elicited by the modified vaccine as compared to the prototype vaccine, using a noninferiority margin of <5% for seroconversion rate difference.

The use of the prototype vaccine (such as ARCT-154) as a comparator is not feasible based on the current recommendations for COVID-19 vaccine immunization, especially in participants of high risk of severe COVID-19. As such, individuals who received the primary vaccination series and one booster dose of the US-authorized mRNA vaccines and the second booster dose of ARCT-154 vaccine in study ARCT-154-J01 will be used as a comparator. All participants who received vaccination according to the protocol, were seronegative for anti-nucleoprotein antibodies, provided pre- and post-vaccination blood samples, and were included in the per protocol set 1 will be included in the comparator group.

The criteria for demonstration of superiority (GMT ratio) and noninferiority (SCR difference) are consistent with the US FDA guidance.

The recommended interval between the primary series and the booster is variable between countries and also depends on the vaccine used for primary vaccination as well as on the pandemic evolution with a potential reduction in protection due to the emergence of a new variant. Despite the variability among countries in their implementation of booster vaccination

campaigns, an interval of 5-6 months is usually observed after primary vaccination with most authorized vaccines.

The study will also assess the feasibility of co-administration of ARCT-2303 vaccine with widely used inactivated quadrivalent influenza vaccines.

mRNA vaccines have the advantage in terms of the ability to adapt recommendations regarding the strain composition and deliver significant quantities of vaccines within a short time. A self-amplifying mRNA vaccine might have additional advantages, including extended duration and magnitude of antigen expression, strong and durable induction of neutralizing antibody and T cell immunity, dose sparing, and the potential to develop a refrigerator-stable product.

2.2.1 Scientific Rationale for the Study Design

The design, conduct, and analysis of this study comply with international and regional standards for clinical research in humans and for investigating the efficacy, immunogenicity, and safety of COVID-19 investigational vaccines.

FDA's Vaccines and Related Biological Products Advisory Committee (VRBPAC) met on June 15, 2023, to discuss and make recommendations for SARS-CoV-2 strain(s) for updated COVID-19 vaccines for use in the United States beginning in the fall of 2023. For the 2023-2024 formulation of the COVID-19 vaccines for use in the US beginning in the fall of 2023, the committee unanimously voted that the vaccine composition be updated to a monovalent COVID-19 vaccine with an XBB-lineage of the Omicron variant. Similar recommendations were also made by EMA and European Centre for Disease Prevention and Control (ECDC-EMA, 2023) and the WHO (Statement on the antigen, 2023).

The study will include an investigational Omicron XBB.1.5 self-amplifying RNA COVID-19 vaccine. In the phase 1/2/3 clinical study, the parent vaccine (ARCT-154), including the ancestral SARS-CoV-2 strain, induced a robust immune response and clinical protection against COVID-19 caused by the prevalent SARS-CoV-2 lineage and showed a good tolerability and safety profile.

The ability of ARCT-154 to induce booster response in individuals who received primary vaccination series with authorized COVID-19 vaccines was assessed in study ARCT-165-01. In this trial, a single dose of ARCT-154 significantly increased the neutralizing antibody titers against ancestral SARS-CoV-2 strain and various variants of concern. The safety and tolerability profile of the booster dose were similar to those of the primary vaccination series.

As of April 2023, most of the population in developed and low-and middle-income countries received primary vaccination and one booster dose of authorized COVID-19 vaccines. Overall, more than 13.3 billion doses of COVID-19 vaccines were distributed globally, and more than 5.1 billion people are considered fully vaccinated, according to the WHO (Our World in Data, 2023). In EU/EEA countries, 73.1% of individuals received the primary vaccination series, and 71% received at least one booster dose of COVID-19 vaccines (European Centre for Disease Prevention and Control, 2023). In the US, 84.6% of individuals completed the primary vaccination series, and 33.1% received a bivalent booster dose of the COVID-19 vaccine (US CDC, 2023). It is anticipated that, at the time of this study initiation, a significant proportion of the population will receive at least three doses of COVID-19 vaccines. To assess the immunogenicity and safety of ARCT-2303 in a homogeneous population,

May not be used, divulged, published or otherwise disclosed without written consent of Arcturus Therapeutics, Inc.

individuals who received the primary vaccination series and at least one booster dose will be recruited.

The study is active-controlled. At Day 1, all participants will receive 2 vaccine doses in opposite arms (ARCT-2303 and influenza vaccine, ARCT-2303 and placebo, or influenza vaccine and placebo). The assessment of immunogenicity will be performed 28 days after vaccination.
To provide equal benefit from the participation in the study and complete seasonal vaccination against COVID-19 and influenza, a switchover vaccine dose (influenza, COVID-19, or placebo) will be administered on Day 29. It allows to keep the blinded design during the entire study period.

Due to the visual differences between the study vaccines, the participants will be blinded before receiving the injection in order to maintain the blinded design of the study. Therefore, the study includes measures to ensure study personnel remains blinded by assigning specific trained and unblinded medical personnel to administer the vaccine. See Section 6.4 for more details.

The duration of immune response after booster vaccination with ARCT-2303 is unknown at the time of study initiation. As such, the kinetics of the immune response after the booster dose and antibody persistence will be assessed at 6 months after vaccination.

The occurrence of new SARS-CoV-2 variants of concern (such as Delta, Omicron, and others) raises questions about the sufficiency of protection following primary and booster vaccinations. To address this question, the study may include an assessment of neutralizing antibodies against epidemiologically relevant SARS-CoV-2 variants of concern.

2.2.2 Justification for Dose and Dose Regimen

In this study, the administration of one dose of ARCT-2303 vaccine given as a booster after primary vaccination and a booster dose of the US-authorized COVID-19 vaccines will be evaluated with the same formulation and total mRNA dose as for the pivotal phase 1/2/3 efficacy and safety study (ARCT-154-01).

2.3 Benefit/Risk Assessment

2.3.1 Risk Assessment

The following section outlines the risk assessment and mitigation strategy for this study protocol (Table 2 and Table 3):

CONFIDENTIAL

May not be used, divulged, published or otherwise disclosed without written consent of Arcturus Therapeutics, Inc.

Table 2 Important Risks Associated with ARCT-2303 Vaccine and Mitigation Strategy

Important Risks	Data/Rationale for Risk	Mitigation Strategy
**Potential Risks**		
Hypersensitivity including allergic reactions such as anaphylaxis	Acute allergic reactions, such as an anaphylactic event, may occur with any vaccine administration. These are serious, but rare, occurrences estimated in the range of 1 to 10 cases per million of vaccinations, depending on the vaccine studied (Rüggeberg et al., 2007).<br>Based on the experience with other mRNA vaccines, there is a possibility of hypersensitivity reactions including anaphylaxis. In the phase 1/2/3 study, twelve cases of hypersensitivity were reported in >17,000 study participants. The events reported were mostly mild or moderate in intensity and few severe events were reported as well. The events were treated with standard clinical care, including antihistamines and steroids. For further details, please refer to the IB.	The onset of vaccine related allergic symptoms is typically within a few minutes. To be able to treat a participant with a serious allergic reaction to the vaccination, the participant will need to remain under observation (visibly followed; no specific procedure) at the study center for 30 minutes post-vaccination with appropriate medical treatment readily available, in case it is needed.<br>Individuals who have a history of severe adverse reaction associated with any vaccine or severe allergic reaction (e.g., anaphylaxis) to any component of the study vaccines will be excluded.
Myocarditis and pericarditis	Myocarditis and pericarditis have been reported in greatest numbers in males under the age of 40 years following a second dose of mRNA vaccines, but cases have been reported in older males and in females as well, and also following other doses. The observed risk is highest in males 12 to 17 years of age. While some cases required intensive care support, available data from short-term follow-up suggest that symptoms resolve in most individuals with conservative management. Information is not yet available about potential long-term sequelae.<br>No cases of myocarditis or pericarditis have been observed to date with COVID-19 Arcturus vaccines (ARCT-021, ARCT-154, ARCT-165).	During the informed consent process, the participants enrolling in the study will be informed of this potential risk and the need to attend the study center if they are unwell. Myocarditis and pericarditis are AESIs and will be collected during the entire study period. All reported cases of myocarditis and pericarditis will be reviewed by medical monitors (blinded review), DSMB (unblinded review) and DSMB will make recommendations as appropriate. In addition, a central cardiac adjudication committee may meet to evaluate ECGs, cardiac enzyme results and other relevant data.

CONFIDENTIAL

May not be used, divulged, published or otherwise disclosed without written consent of Arcturus Therapeutics, Inc.

Table 2 Important Risks Associated with ARCT-2303 Vaccine and Mitigation Strategy

Important Risks	Data/Rationale for Risk	Mitigation Strategy
**Other Considerations**		
Local and systemic reactions	IM vaccination commonly precipitates a transient and self-limiting local inflammatory reaction. This may typically include pain, erythema, and swelling at the injection site.<br>In addition, systemic reactions (fatigue, headache, myalgia, arthralgia, diarrhea, nausea, chills, dizziness, and fever) can also occur soon after vaccine administration.<br>In Study ARCT-154-01, overall, a trend to a higher frequency of solicited local AEs (primarily of mild-to-moderate severity) was observed after administration of the ARCT-154 vaccine compared to placebo. Injection site pain and tenderness were the most frequently reported local symptoms.<br>The most frequently reported systemic solicited AEs were fatigue, headache, and myalgia.<br>The majority of solicited systemic AEs were mild or moderate in intensity. Solicited AEs were transient and mostly resolved within 3 days. Frequencies of solicited systemic AEs were generally lower after the second dose than after the first dose	Any untoward symptoms experienced by the participant after receiving the vaccine should be reported to the investigator.<br>To ensure the safety of the study participants, ongoing unblinded safety monitoring will be performed by the DSMB on a regular basis.<br>All participants will remain under observation at the study center for 30 minutes post vaccination.<br>The following solicited AEs will be monitored for 7 days after vaccination:<br>Injection site AEs: pain, erythema, and swelling.<br>Systemic AEs: fatigue, headache, myalgia, arthralgia, nausea, dizziness, chills, and fever.

Abbreviations: AE, adverse event; AESI: adverse event of special interest; DSMB, data safety monitoring board; ECG, electrocardiogram; IB, Investigator's Brochure; IM, intramuscular.

Table 3 Risks Related to Study Procedures

Venipuncture (blood sampling)	Pain or bruising at the site where blood is drawn.	Blood samples will be collected by trained medical personnel; participants will remain under medical observation after venipuncture to complete study-specific procedures.
Syncope	Syncope (fainting) can occur following or even before any blood draws as a psychogenic or vasovagal response to the needle injection.	All participants will remain under observation, post venipuncture, through completion of the applicable study visit.

2.3.2 Benefit Assessment

The study participants may benefit directly from participation in this study if the vaccinations induce a protective immune response against SARS-CoV-2 and influenza when and if these participants are subsequently exposed to these viruses. Participants will gain some information about their general health, including their status of SARS-CoV-2 infection, a result of the screening history, examination, blood tests, and urine tests (if applicable). Participants found to have a previously undiagnosed condition considered to require further medical attention will be referred appropriately for further investigation and treatment, with their permission. It is hoped that their contribution to this study will further support the successful development of a safe and effective vaccine against COVID-19 and provide knowledge about SARS-CoV-2 infection and the mechanism of protection.

2.3.3 Overall Benefit/Risk Conclusion

Considering the measures that will be implemented to minimize the risks to participants participating in this study, the potential and/or known risks identified in association with the ARCT-2303 vaccine is justified by the anticipated benefits for humans to prevent SARS-CoV-2 infection and COVID-19.

The Sponsor will immediately notify the principal investigator and the data safety monitoring board (DSMB) if any additional significant new safety or toxicology information becomes available during the study.

This study will be performed in compliance with the protocol, International Council for Harmonisation (ICH) Good Clinical Practice (GCP) and applicable regulatory requirements. Aspects of the study concerned with the investigational medicinal product (ARCT-2303) will meet the requirements of Good Manufacturing Practice (GMP).

More detailed information about the known and expected benefits and risks, and the reasonably expected AEs of the ARCT-2303 vaccine can be found in the IB.

CONFIDENTIAL

May not be used, divulged, published or otherwise disclosed without written consent of Arcturus Therapeutics, Inc.

3 STUDY DESIGN

This is a multicenter, observer-blind, randomized, controlled phase 3 study to evaluate the immunogenicity, reactogenicity, and safety of the investigational COVID-19 vaccine ARCT-2303 administered concomitantly with Quadrivalent Influenza Vaccines or standalone in adults who previously received primary vaccination series and at least one booster dose of the US-authorized mRNA COVID-19 vaccines.

3.1 Overall Design

Approximately 1680 participants will be enrolled in this study.

Study arms and vaccination schedule:

Participants who received at least 3 doses (a 2-dose primary series and at least one booster dose) of the US-authorized mRNA COVID-19 vaccines, with the last mRNA booster dose (original strain or bivalent) ≥5 months before enrollment will be recruited in one of the two cohorts.

Cohort A (participants 18 to 64 years of age; N=1200)

Individuals will be randomly assigned to one of the three study groups:

- Group 1a (ARCT-2303/ Flucelvax Quadrivalent, N=400): participants will receive one dose of ARCT-2303 and one dose of Flucelvax Quadrivalent (opposite arms) on Day 1, and one dose of placebo on Day 29.
- Group 2a (ARCT-2303, N=400): participants will receive one dose of ARCT-2303 and one dose of placebo (opposite arms) on Day 1, and one dose of Flucelvax Quadrivalent on Day 29.
- Group 3a (Flucelvax Quadrivalent, N=400): participants will receive one dose of Flucelvax Quadrivalent and one dose of placebo (opposite arms) on Day 1, and one dose of ARCT-2303 on Day 29.

Cohort B (participants ≥65 years of age; N=480)

Individuals will be randomly assigned to one of the three study groups:

- Group 1b (ARCT-2303/ Fluad Quadrivalent, N=160): participants will receive one dose of ARCT-2303 and one dose of Fluad Quadrivalent (opposite arms) on Day 1, and one dose of placebo on Day 29.
- Group 2b (ARCT-2303, N=160): participants will receive one dose of ARCT-2303 and one dose of placebo (opposite arms) on Day 1, and one dose of Fluad Quadrivalent on Day 29.

- Group 3b (Fluad Quadrivalent, N=160): participants will receive one dose of Fluad Quadrivalent and one dose of placebo (opposite arms) on Day 1, and one dose of ARCT-2303 on Day 29.

In addition, all available serum samples from study participants of study ARCT-154-J01, who received a booster dose of candidate ARCT-154 vaccine according to the protocol, provided pre- and post-vaccination blood samples and did not have evidence of SARS-CoV-2 infection and major protocol deviations, will be selected to use as a comparator. This set of samples will compose up to 385 individuals 18 years of age and above, who were included in the per protocol set 1 (PPS-1). These samples will be tested in this study for assessment of coprimary study objectives 1 and 2.

Study design rationale: The coprimary immunogenicity objectives will be assessed on Days 1 and 29. Immunological superiority, as measured by VNA against Omicron XBB.1.5 subvariant, between a booster dose of ARCT-2303 and the ARCT-154 booster vaccine (study ARCT-154-J01) will be demonstrated for the ratio of the GMTs. In addition, a co-primary noninferiority immunogenicity objective for the difference in seroconversion rates (SCRs) will be tested for the same subvariant Omicron XBB.1.5. As the second step, noninferiority of immune response after co-administration and standalone administration will be tested for ARCT-2303 and QIV vaccines.

To provide the opportunity to receive vaccination against COVID-19 and influenza diseases for all study participants and keep the blinded status of the study, a switchover vaccine dose or placebo will be administered on Day 29 (after the blood draw for serology testing).

Study success criteria: The success criterion of the study is that the coprimary immunogenicity objectives 1 and 2 (Table 4) are demonstrated. If coprimary objectives 1 and 2 are demonstrated, then the coprimary noninferiority objectives 3 and 4 (Table 4) will be tested; noninferiority of immune response after co-administration and standalone administration of ARCT-2303 and Flucelvax Quadrivalent will be demonstrated if all 5 comparisons (four for influenza vaccine antigens and one for ARCT-2303) are successful. If all coprimary objectives 1, 2, 3, and 4 are demonstrated, secondary objectives will be tested by applying a sequential testing strategy as follows: if superiority of SCR (secondary immunogenicity objective 2; Table 4) is demonstrated then super superiority of GMTs (secondary immunogenicity objective 1; Table 4) will be tested.

Randomization: An interactive response technology will be used. Within each age cohort (A and B), participants will be randomly assigned at a 1:1:1 ratio into three study groups. Randomization will be stratified by COVID-19 vaccination history (such as, a total number of COVID-19 vaccine doses received and the composition of the last booster dose (mRNA original strain or mRNA bivalent)).

Blinding: Observer-blind.

Study visits: Four planned study visits (Days 1, 29, 57, and 181).

CONFIDENTIAL

May not be used, divulged, published or otherwise disclosed without written consent of Arcturus Therapeutics, Inc.

Safety call: One planned safety call (Day 91).

Number of blood samples: Three blood draws (Days 1, 29, and 181) will be obtained from all participants.

Duration of the study/participant participation: Approximately 6 months for each participant.

Sample selection criteria for ARCT-154-J01 study participants:

All participants of study ARCT-154-J01 with available pre- and post-vaccination (Days 1 and 29) serum samples, who were allocated in the ARCT-154 group and were included in the PPS-1 for primary immunogenicity analysis (up to 385 participants) will be used as a control group.

The study design is provided in Figure 1. The schedule of assessments (SoA) is provided in Table 1.

3.2 End of Study Definition

The end of the study is defined as the date of the last visit of the last participant in the study or last scheduled procedure shown in the SoA (Table 1) for the last participant in the study. The duration of participation will be approximately 6 months for each participant.

CONFIDENTIAL

May not be used, divulged, published or otherwise disclosed without written consent of Arcturus Therapeutics, Inc.

4 OBJECTIVES, ENDPOINTS AND ESTIMANDS

The study objectives, endpoints, and estimands are listed in Table 4.

Table 4 Objectives, Endpoints and Estimands

Primary Immunogenicity Objective	Endpoints	Estimands
1. To demonstrate that the ARCT-2303 vaccine, when given as a booster dose, elicits an immune response that is superior (simple superiority[a]) to those after a booster dose of ARCT-154 (in study ARCT-154-J01), as measured by GMTs of neutralizing antibodies against Omicron XBB.1.5 subvariant	SARS-CoV-2 neutralizing antibody titers against Omicron XBB.1.5 subvariant in:<br>• ARCT-2303 vaccine recipients (Groups 2a and 2b) on Day 29<br>• ARCT-154-J01 study participants who received a booster dose of ARCT-154 vaccine, on Day 29	• GMT ratio of $GMT_{ARCT\text{-}2303\ (Groups\ 2a\ and\ 2b)}$ (Day 29) over $GMT_{ARCT\text{-}154\ (a\ booster\ dose)}$ (Day 29)<br>by PP set for immunogenicity.
2. To demonstrate that the ARCT-2303 vaccine, when given as a booster dose, elicits an immune response that is noninferior to those after a booster dose of ARCT-154 (in study ARCT-154-J01), as measured by SARS-CoV-2 neutralizing antibody seroconversion rate with Omicron XBB.1.5 subvariant	SARS-CoV-2 neutralizing antibody seroconversion rates against Omicron XBB.1.5 subvariant in:<br>• ARCT-2303 vaccine recipients (Groups 2a and 2b) on Day 29<br>• ARCT-154-J01 study participants who received a booster dose of ARCT-154 vaccine, on Day 29	• SCR difference of $SCR_{ARCT\text{-}2303\ (Groups\ 2a\ and\ 2b)}$ (Day 29) minus $SCR_{ARCT\text{-}154\ (a\ booster\ dose)}$ (Day 29)<br>by PP set for immunogenicity.
3. To demonstrate that vaccination with Flucelvax Quadrivalent, when given concomitantly with ARCT-2303, elicits an immune response that is noninferior to that of Flucelvax Quadrivalent, when given standalone, as measured by serum Hemagglutination Inhibition (HI) assay,[b] at 28 days after vaccination	HI assay titers against influenza vaccine strains on Day 29 in:<br>• Co-admin group recipients (Group 1a)<br>• Standalone Flucelvax Quadrivalent group recipients (Group 3a)	• HI geometric mean titer (GMT) ratio of $GMT_{Co\text{-}admin\ (Group\ 1a)}$ (Day 29) over $GMT_{Standalone\ Flucelvax\ (Group\ 3a)}$ (Day 29)<br>For each influenza vaccine strain (4 strains), by PP set for immunogenicity.

May not be used, divulged, published or otherwise disclosed without written consent of Arcturus Therapeutics, Inc.

Table 4 Objectives, Endpoints and Estimands

4. To demonstrate that vaccination with ARCT-2303, when given concomitantly with Flucelvax Quadrivalent, elicits an immune response that is noninferior to that of ARCT-2303, when given standalone, as measured by GMTs of neutralizing antibodies against Omicron XBB.1.5 subvariant, at 28 days after vaccination	SARS-CoV-2 neutralizing antibody responses against Omicron XBB.1.5 subvariant on Day 29, in:<br>• Co-admin group recipients (Group 1a)<br>• Standalone ARCT-2303 group recipients (Group 2a)	• GMT ratio of GMT $_{\text{Co-admin (Group 1a)}}$ (Day 29) over GMT $_{\text{Standalone ARCT-2303 (Group 2a)}}$ (Day 29)<br>by PP set for immunogenicity.
**Secondary Immunogenicity Objectives**		
1. To demonstrate that the ARCT-2303 vaccine, when given as a booster dose, elicits an immune response that is superior (super superiority[a]) to those after a booster dose of ARCT-154 (in study ARCT-154-J01), as measured by GMTs of neutralizing antibodies against Omicron XBB.1.5 subvariant	SARS-CoV-2 neutralizing antibody titers against Omicron XBB.1.5 subvariant in:<br>• ARCT-2303 vaccine recipients (Groups 2a and 2b) on Day 29<br>• ARCT-154-J01 study participants who received a booster dose of ARCT-154 vaccine, on Day 29	• GMT ratio of GMT$_{\text{ARCT-2303 (Groups 2a and 2b)}}$ (Day 29) over GMT$_{\text{ARCT-154 (a booster dose)}}$ (Day 29)<br>by PP set for immunogenicity.
2. To demonstrate that the ARCT-2303 vaccine, when given as a booster dose, elicits an immune response that is superior to those after a booster dose of ARCT-154 (in study ARCT-154-J01), as measured by SARS-CoV-2 neutralizing antibody seroconversion rate with Omicron XBB.1.5 subvariant	SARS-CoV-2 neutralizing antibody seroconversion rates against Omicron XBB.1.5 subvariant in:<br>• ARCT-2303 vaccine recipients (Groups 2a and 2b) on Day 29<br>• ARCT-154-J01 study participants who received a booster dose of ARCT-154 vaccine, on Day 29	• SCR difference of SCR$_{\text{ARCT-2303 (Groups 2a and 2b)}}$ (Day 29) minus SCR$_{\text{ARCT-154 (a booster dose)}}$ (Day 29)<br>by PP set for immunogenicity.
3. To assess the immunogenicity of ARCT-2303 vaccine when administered with or without Flucelvax Quadrivalent, as measured by virus neutralization assay	SARS-CoV-2 neutralizing antibody responses against Omicron XBB.1.5 subvariant on Days 1, 29, and 181 (a subset of samples):<br>• Co-admin group recipients (Group 1a)<br>• Standalone ARCT-2303 group recipients (Group 2a)	• GMTs<br>• GMFRs (post/pre-vaccination)<br>• SCRs[b]<br>• Proportion of participants with antibody titer ≥ LLOQ<br>by PP set for immunogenicity.

Table 4 Objectives, Endpoints and Estimands

4. To assess the immunogenicity of Flucelvax Quadrivalent when administered with or without ARCT-2303 as measured by HI assay[c]	HI assay titers against influenza vaccine strains on Day 1 and Day 29, in:<br>• Co-admin group recipients (Group 1a)<br>• Standalone Flucelvax Quadrivalent group recipients (Group 3a)	• GMTs<br>• SCRs<br>• GMFRs (post/pre-vaccination)<br>• Proportion of participants with HI titer ≥1:40<br>For each influenza vaccine strain, by PP set for immunogenicity.
5. To assess the immunogenicity of ARCT-2303 vaccine, when administered with or without Fluad Quadrivalent, as measured by virus neutralization assay, in participants ≥65 years of age.	SARS-CoV-2 neutralizing antibody responses against Omicron XBB.1.5 subvariant on Days 1, 29, and 181:<br>• Co-admin group recipients (Group 1b)<br>• Standalone ARCT-2303 group recipients (Group 2b)	• GMTs<br>• GMFRs (post/pre-vaccination)<br>• SCRs[b]<br>• Proportion of participants with antibody titer ≥ LLOQ<br>by PP set for immunogenicity.
6. To assess the immunogenicity of Fluad Quadrivalent when administered with or without ARCT-2303 as measured by HI assay,[c] in participants ≥65 years of age	HI assay titers against influenza vaccine strains on Day 1 and Day 29, in:<br>• Co-admin group recipients (Group 1b)<br>• Standalone Fluad Quadrivalent group recipients (Group 3b)	• GMTs<br>• SCRs<br>• GMFRs (post/pre-vaccination)<br>• Proportion of participants with HI titer ≥1:40<br>For each influenza vaccine strain, by PP set for immunogenicity.
**Secondary Safety Objectives**		
1. To assess the safety and reactogenicity of the study vaccines when given in co-administration or standalone	• Local and systemic AEs reported within 7 days after each study vaccination<br>• Unsolicited AEs reported within 28 days after each vaccination<br>• SAE, AEs leading to early termination from study, MAAEs, and AESIs during the entire study period (6-month follow-up)	• Proportion of participants with local and systemic solicited AEs;<br>• Proportion of participants with unsolicited AEs;<br>• Proportion of participants with SAEs;<br>• Proportion of participants with MAAEs;<br>• Proportion of participants with AESIs;<br>• Proportion of participants with AEs leading to early termination from study;<br>by Safety set.

May not be used, divulged, published or otherwise disclosed without written consent of Arcturus Therapeutics, Inc.

Table 4 Objectives, Endpoints and Estimands

Exploratory Immunogenicity Objectives		
1. To assess the immune response elicited by the ARCT-2303 vaccine against a panel of SARS-CoV-2 strains, as measured by VNA	SARS-CoV-2 neutralizing antibody responses with a panel of historical and new emergent SARS-CoV-2 strains on Days 1 and 29 in a subset of:<br>• Standalone ARCT-2303 group recipients (Group 2a and/or 2b)<br>• Standalone QIV group recipients (Group 3a and/or 3b)	• GMTs<br>• GMFRs (post/pre-vaccination)<br>• SCRs<br>• Proportion of participants with antibody titer ≥ LLOQ<br>by PP set for immunogenicity.
2. To assess the immunogenicity of influenza vaccine(s) when administered with or without ARCT-2303 as measured by microneutralization assay (in a subset of participants)	Microneutralization (MN) titers against influenza vaccine strains on Day 1 and Day 29, in:<br>• Co-admin group recipients (a subset of participants from Group 1a and/or 1b)<br>• Standalone QIV group recipients (a subset of participants from Group 3a and/or 3b).	• GMTs<br>• GMFRs (post/pre-vaccination)<br>• SCRs<br>• Proportion of participants with antibody titer ≥ LLOQ<br>For each influenza vaccine strain, by PP set for immunogenicity.

Abbreviations: AE, adverse event; AESI, adverse event of special interest; GMFR, geometric mean fold rise; GMT, geometric mean titer; HI, Hemagglutination Inhibition; LLOQ, lower limit of quantification; MAAE, medically attended AEs; MN, microneutralization; PP, per protocol; QIV, quadrivalent influenza vaccine; SAE, serious adverse event; SARS-CoV-2, severe acute respiratory syndrome coronavirus 2; SCR, seroconversion rate; VNA, virus neutralization assay.

[a] As per FDA guidance: "simple" superiority (margin of >1-fold for GMT ratio); "super" superiority (margin of >1.5-fold for GMT ratio). US FDA Guidance for Industry. Emergency Use Authorization for Vaccines to Prevent COVID-19. Document issued on March 31, 2022.

[b] SCR will not be performed on Day 181.

[c] In case of lack of agglutination for a specific strain using HI assay, immunogenicity for that strain will be assessed as measured by microneutralization (MN) assay as an acceptable alternative.

Seroconversion rate (SCR) is defined as the percentage of participants with a ≥4-fold increase in titer from that at Day 1 (or from LLOQ if Day 1 titer <LLOQ).
For definitions of the PP set for immunogenicity and other populations for analysis, refer to Section 10.3 of protocol.

May not be used, divulged, published or otherwise disclosed without written consent of Arcturus Therapeutics, Inc.

5 STUDY POPULATION

and none of the criteria listed in Section 5.2 apply.

5.1 Inclusion Criteria

Participants are eligible to be included in the study only if all of the following criteria apply:

1. Individuals are male, female, or transgender adults ≥18 years of age.
2. Healthy participants or participants with pre-existing stable medical conditions. Pre-existing stable medical condition means a participant who: has full capacity of daily activity and no major medication modification; has not undergone surgical or minimally invasive intervention or had any hospitalization/emergency room visit for the specific medical condition within 3 months prior to Day 1.
3. Participant or legally authorized representatives must freely provide documented informed consent prior to study procedures being performed.
4. Individuals must have been previously vaccinated with COVID-19 vaccines as follows:

- Received at least 3 doses (a 2-dose primary series and at least one booster dose) of the US-authorized mRNA COVID-19 vaccines.
- The last booster dose must be the US-authorized mRNA COVID-19 vaccine (original strain or bivalent), administered ≥5 months prior to enrollment.
- Receipt of COVID-19 vaccines is supported by documentation.

5. Individuals must agree to comply with all study visits and procedures (including blood tests, nasopharyngeal swabs, diary completion, receipt of telephone calls from the site, willingness to be available for Unscheduled Clinic Visits).
6. Individuals of childbearing potential must be willing to adhere to protocol contraceptive requirements (see Appendix 1) and local regulations.

5.2 Exclusion Criteria

Participants are excluded from the study if any of the following criteria apply:

1. Individuals with acute medical illness or febrile illness, including temperature ≥100.4°F (≥38.0°C; measured by any method) within 3 days prior to Randomization. These individuals may be offered the opportunity to enter the study after fever and illness has stabilized. Participants with suspected or confirmed COVID-19 should be excluded and referred for medical care. Rescreening will be permitted for individuals who are presented with suspected COVID-19 only if COVID-19 has been ruled out based on laboratory-confirmed or healthcare provider-conducted virological testing (such as RT-PCR or rapid antigen test).

May not be used, divulged, published or otherwise disclosed without written consent of Arcturus Therapeutics, Inc.

2. Individuals with a positive SARS-CoV-2 rapid antigen test at Screening (RT-PCR test may be performed according to institutional policy in addition to rapid antigen testing but will not be considered a screening requirement and should not delay vaccine administration).
3. Individuals with a history of COVID-19 or virologically confirmed SARS-CoV-2 infection within the past 5 months or history of COVID-19 with ongoing sequelae.
4. Individuals with any medical, neurological, or psychiatric condition that, in the opinion of the investigator, could place the participant at an unacceptable risk of injury or render the participant unable to comply with all study procedures and achieve successful completion of the trial.
5. Individuals with a known history of severe hypersensitivity reactions, including anaphylaxis, or other significant adverse reactions to any vaccine, any components of mRNA vaccine or influenza vaccine, including egg protein.
6. Individuals who have a positive pregnancy test at the Screening visit or who intend to become pregnant or breastfeed during the study.
7. Individuals with a history of myocarditis, pericarditis, myopericarditis or cardiomyopathy.
8. Individuals with a history of Guillain-Barré syndrome, encephalomyelitis, or transverse myelitis.
9. Individuals with a known bleeding disorder that would, in the opinion of the investigator, contraindicate intramuscular (IM) injection.
10. Individuals with a history of congenital or acquired immunodeficiency.
11. Individuals who have received immunomodulatory, immunostimulatory, or immunosuppressant drugs including interferon and cytotoxic drugs within 3 months of Screening/Day 1 or who plan to receive them during the study.
12. Individuals requiring systemic corticosteroids exceeding 10 mg/day of prednisone equivalent for ≥10 days within 30 days of Screening. The use of topical, ophthalmic, inhaled, and intranasal steroid preparations will be permitted.
13. Individuals who have received immunoglobulins and/or any blood or blood products within the 3 months before the first vaccine administration or plan to receive such products at any time during the study.
14. Individuals with an immunosuppressive or immunodeficient state, asplenia, or recurrent severe infections.
15. Individuals with a documented history of HIV infection, or who are currently known to have active tuberculosis.
16. Individuals with chronic illness that, in the opinion of the Investigator, are at a stage where it might interfere with trial participation or interpretation of study results.
17. Individuals receiving treatment with another investigational drug, biological agent, or device within 28 days of screening, or 5 half-lives of the investigational drug, whichever

CONFIDENTIAL

May not be used, divulged, published or otherwise disclosed without written consent of Arcturus Therapeutics, Inc.

is longer; or are currently enrolled in or plan to participate in another clinical trial with an investigational agent during the period.

18. Individuals who have received any investigational COVID-19 vaccines.
19. Individuals who received any influenza vaccine within 6 months prior to enrollment or plan to receive an influenza vaccine during the study period.
20. Individuals who have received any other licensed vaccines within 14 days prior to enrollment in this study or who are planning to receive any vaccine up to 14 days after the second study vaccination.
21. Individuals who are investigator site staff members, employees of the Sponsor or the Clinical Research Organization directly involved in the conduct of the study, or site staff members otherwise supervised by the investigator or immediate family members of any of the previously mentioned individuals.

5.3 Contraindications for the Second Vaccination

The following events constitute absolute contraindications to further administration of study vaccines.

If any of these events occur during the study, the participant must not receive additional doses of vaccine, but should be encouraged to continue study participation for safety at the discretion of the investigator:

- Anaphylaxis or systemic hypersensitivity reaction following the administration of vaccine.
- Any SAE judged to be related to study vaccination.
- Pregnancy.
- Any adverse event that, in the opinion of the investigator, poses an additional risk to the participant if he/she continues to participate in the study.

The following events constitute contraindications to administration of study vaccine at certain points in time, and if any of these events occur at the time scheduled for vaccination, the participant may be vaccinated at a later date, preferably within the time window specified in the protocol (Table 1), or the participant may be withdrawn at the discretion of the investigator:

- Any COVID-19 that occurred after Visit 1 or a positive result of COVID-19 test pre-vaccination.
- Acute moderate or severe infection with or without fever at the time of vaccination.
- Fever, defined as temperature ≥38.0°C (100.4°F; measured by any method) at the time of vaccination.

Participants with a minor illness without fever, as assessed by the investigator, can be administered study vaccines.

5.4 Prior and Concomitant Medications

The use of prior and concomitant medications must be recorded in the electronic case report form (eCRF).

CONFIDENTIAL

May not be used, divulged, published or otherwise disclosed without written consent of Arcturus Therapeutics, Inc.

- Prior medications, vaccines, and blood products that are described in the exclusion criteria (Section 5.2) are to be recorded on the eCRF, if they were administered to any enrolled individual. It includes:
  - Receipt of any licensed or investigational COVID-19 vaccine prior to Day 1;
  - Receipt of any investigational product within 28 days prior to Day 1 (or 5 half-lives of the investigational product, if longer);
  - Immunomodulatory, immunostimulatory, or immunosuppressive therapy, including interferon and cytotoxic agents, within 90 days prior to Day 1;
  - Systemic corticosteroids exceeding 10 mg/day of prednisone equivalent for ≥10 days within 30 days prior to Day 1;
  - Receipt of any influenza vaccine within 6 months prior to Day 1;
  - Receipt of any licensed vaccines within 14 days prior to Day 1;
  - Intravenous immunoglobulins and/or any blood or blood products within 3 months prior to Day 1.
- Concomitant medications include medications and vaccines taken by/administered to the participant at and after enrollment and must be documented on the eCRF. The following are considered concomitant medications:
  - All medications and vaccines administered from Visit 1 (day of vaccination) up to 28 days after each study vaccination;
  - All medications associated with SAEs, adverse events of special interest (AESIs), medically attended AEs (MAAEs), and AEs leading to early study termination, during the entire study period (from Visit 1 to study completion);
  - All medications administered for treatment of COVID-19 during the entire study period (from Visit 1 to study completion);
  - Any licensed or investigational COVID-19 vaccine during the entire study period (from Visit 1 to study completion);
  - Any investigational and non-licensed medications (other than the study vaccines) during the entire study period (from Visit 1 to study completion).
- Any medication that meets the reporting criteria (including over-the-counter or prescription medicines, and/or herbal supplements) must be recorded on the eCRF along with:
  - Reason for use;
  - Dates of administration including start and end dates;
  - Dosage information including dose, route of administration and frequency.

The use of antipyretics (e.g., paracetamol) will be allowed as treatment for fever and pain or other post-vaccination reactions; participants should not be encouraged to use antipyretics prophylactically. The medical monitor should be contacted if there are any questions regarding concomitant or prior therapy.

CONFIDENTIAL

May not be used, divulged, published or otherwise disclosed without written consent of Arcturus Therapeutics, Inc.

5.5 Prohibited Medications

The use of an excluded medication/therapy is a protocol violation and must be recorded in the eCRF. The use of an excluded medication does not require the withdrawal of a participant from the study.

The following medications are prohibited:

- Receipt of any licensed or investigational COVID-19 vaccine during the study period;
- Receipt of any licensed or investigational influenza vaccine within 6 months prior to Day 1 or during the study period;
- Receipt of any licensed vaccines within 14 days prior to Day 1 or up to 14 days after the second study vaccination;
- Receipt of any investigational product within 28 days prior to Day 1 (or 5 half-lives of the investigational product, if longer) or during the study period;
- Immunomodulatory, immunostimulatory, or immunosuppressive therapy, including interferon and cytotoxic agents, within 90 days prior to Day 1 or during the study period;
- Systemic corticosteroids exceeding 10 mg/day of prednisone equivalent for ≥10 days within 30 days prior to Day 1 or during the study period;
- Intravenous immunoglobulins and/or any blood products within 3 months prior to Day 1 or during the study period.

5.6 Screen Failures

Screen failures are defined as participants who consent to participate in the clinical study but do not meet all eligibility criteria and are not subsequently randomly assigned to a study arm. A minimal set of screen failure information is required to ensure transparent reporting of screen failure participants to meet the Consolidated Standards of Reporting Trials (CONSORT) publishing requirements and to respond to queries from regulatory authorities. The reason for screen failure must be documented in the Screening and Enrollment log.

A participant who does not meet the eligibility criteria can be rescreened once. Participants who are rescreened will receive a new participant number. The informed consent process should be repeated if rescreening occurs ≥ 14 days from previous ICF signature date.

CONFIDENTIAL

May not be used, divulged, published or otherwise disclosed without written consent of Arcturus Therapeutics, Inc.

6 STUDY VACCINES

All vaccines associated with this study are to be stored separately from other vaccines and medications in a secure location under appropriate storage conditions with temperature monitoring. A clinical label will be affixed to study vaccine containers in accordance with local regulatory requirements. All vaccines associated with this study must be checked for expiration date prior to use. Expired study vaccines must not be administered to participants.

6.1 Study Vaccine

The details about investigational ARCT-2303 vaccine are presented in Table 5. The instructions regarding ARCT-2303 vaccine preparation and administration are provided in the Pharmacy Manual. Refer to the IB for further information about the ARCT-2303 vaccine.

Table 5 Study Vaccine Details –ARCT-2303

**Intervention Name**	**ARCT-2303 vaccine**
Formulation	ARCT-2303 Drug Product (DP) contains one Drug Substance (DS):<br>**mRNA-2319** (Omicron XBB.1.5 mRNA)<br>mRNA construct encapsulated in the lipid nanoparticle
Presentation	Lyophilized formulation in a 12-mL-size glass vial
Manufacturer	Designated manufacturer: Arcturus Therapeutics, Inc.
Route of Administration	IM in the deltoid region of the upper arm
Volume (after preparation)	0.5 mL
Composition/0.5 mL dose	**mRNA-2319:** 5.0 µg
IMP	Yes
Packaging	The study vaccines will be packaged and labeled according to GMP and local regulations. The study vaccine will not be packed in individual participant kits; one kit may be used for multiple participants.

Abbreviations: GMP, Good Manufacturing Practice; IM, intramuscular; IMP, investigational medicinal product.

Warnings and Precautions

Only participants enrolled in the study may receive study vaccine and only authorized study staff may supply or administer study vaccine. As there is limited experience with the administration of the study vaccine to humans, all effects cannot be reliably predicted. Availability of facilities and staff familiar with the treatment of anaphylaxis and resuscitation and the treatment of other medical emergencies will be ensured.

6.2 Vaccine Preparation, Handling, Storage and Accountability

6.2.1 Vaccine Preparation

ARCT-2303 study vaccine will be prepared in accordance with instructions provided in the Pharmacy Manual.

6.2.2 Vaccine Handling

The Investigator or designee must confirm appropriate temperature conditions have been maintained during transit for the study vaccine received and any discrepancies are reported and

resolved before use of the study intervention. Only participants enrolled in the study may receive study intervention and only authorized unblinded site staff may supply or administer study intervention.

6.2.3 Vaccine Storage

ARCT-2303 vaccine should be stored at -20ºC ± 5ºC.

Study vaccine must be stored in a secure, environmentally controlled, and monitored (manual or automated recording) area in accordance with the labeled storage conditions with access limited to the Investigator and authorized site staff. At a minimum, daily minimum and maximum temperatures for all site storage locations must be documented and available upon request. Data for nonworking days must indicate the minimum and maximum since previously documented for all site storage locations upon return to business.

Study vaccine temperature storage requirements and monitoring procedures are included within the Pharmacy Manual.

Any excursions from the study vaccine storage conditions should be reported to Arcturus upon discovery along with any actions taken. Once an excursion is identified, the study vaccine must be quarantined and not used until Arcturus provides permission to use the study intervention. Specific details regarding the definition of an excursion and information the site should report for each excursion will be provided to the site in the Pharmacy Manual.

Upon identification of a product complaint, Arcturus should be notified within 1 business day of discovery as described in the Pharmacy Manual.

6.2.4 Vaccine Accountability

The study staff is required to document the receipt, dispensing, and return/destruction of study vaccine supplies provided by the Sponsor. The study site must destroy or return for destruction all unused frozen vials of study vaccine to the Sponsor or designee. Vials of study vaccine should be destroyed by the study site or delegate only after drug accountability has been done by the unblinded study monitor. Quarantining, destruction, or return of study vaccine must be documented and in accordance with guidance provided by the Pharmacy Manual as well as local institutional policies.

6.2.5 Vaccine Administration

Study vaccines will be administered by intramuscular (IM) injection to the deltoid muscle by a qualified, GCP-trained healthcare provider (HCP) who will not be involved in assessments of any study endpoints. Study vaccine preparation and administration should be performed in an area outside of the view of blinded team members. Unblinded team members will not otherwise participate in other study-related procedures or assessments of the participant.

Standard vaccination practices must be observed, and vaccine must not be injected into blood vessels. Appropriate medication and other supportive measures for management of an acute hypersensitivity reaction should be available in accordance with local guidelines for standard immunization practices.

CONFIDENTIAL

May not be used, divulged, published or otherwise disclosed without written consent of Arcturus Therapeutics, Inc.

Study vaccine administration details will be recorded in source documents and on the case report form (CRF). Reasons for departure from the expected dispensing regimen must also be recorded.

6.2.6 Errors in Study Vaccine Administration

Study vaccine errors (including overdose, underdose, and administration error) must be documented as protocol deviations. A brief description should be provided of the deviation, including whether the participant was symptomatic (list symptoms) or asymptomatic, and whether the event was accidental or intentional.

Dosing details should be captured on the appropriate source documents and on the eCRF.

An overdose is the accidental or intentional administration of a study vaccine in an amount higher than the dose being studied. An overdose or incorrect administration of study vaccine is not itself an AE, but it may result in an AE. If the participant receives a dose of study vaccine that exceeds protocol specifications and the participant is symptomatic, the symptom(s) should be documented as an AE (see Section 9).

Should an overdose occur, the Investigator or designee should contact the Sponsor or designee within 24 hours.

6.2.6.1 Management of Overdose

There are no known treatments for potential overdose of study vaccine. Unless there is a user error leading to failure to administer any volume of study vaccine, “catch-up” study vaccine administrations will not be performed. In case of medication errors or an overdose, it is recommended that the participant be monitored for any signs or symptoms of adverse reactions and appropriate symptomatic treatment be provided immediately.

6.3 Licensed Study Vaccines

Flucelvax® Quadrivalent (Seqirus Pty Ltd.) is a surface antigen, inactivated influenza vaccine prepared in cell cultures and indicated for active immunization of persons aged 6 months or older for the prevention of influenza disease. Each 0.5-mL dose, in sterile phosphate-buffered saline, is a suspension of a total of 60 µg hemagglutinin (HA). HA antigens are derived from the four influenza strains (A/H1N1, A/H3N2, B/Yamagata and B/Victoria lineage, 15 µg each) recommended by the World Health Organization (WHO) for quadrivalent vaccines for the respective season.

Fluad® Quadrivalent (Seqirus Pty Ltd.) is a surface antigen, inactivated adjuvanted influenza vaccine indicated for active immunization of persons aged 65 years or older for the prevention of influenza disease. An approximately 0.5-mL dose of vaccine contains nominally 15 µg of HA of each of the 2 influenza type A strains and each of the 2 influenza type B strains for a total of 60 µg of HA in the vaccine. The strain composition is that recommended by the WHO for quadrivalent vaccines for the respective season.

6.4 Measures to Minimize Bias: Randomization and Blinding

This study will be observer-blind.

CONFIDENTIAL

May not be used, divulged, published or otherwise disclosed without written consent of Arcturus Therapeutics, Inc.

After signing the informed consent form (ICF), each participant will be given a screening number according to the screening order. On Day 1, following confirmation of eligibility, a participant number will be assigned, and participants will be allocated to treatment using an interactive response technology (IRT).

Due to the visual differences between the study vaccines, the participants will be blinded before receiving the study vaccinations. An unblinded dosing team, not involved with study participant's evaluation, will prepare and administer the study vaccine doses. The study vaccine syringe will be opacified to avoid unblinding of the participant. The administration of the study vaccine will also be performed behind a closed curtain to ensure that the other blinded staff members do not become unblinded. The investigative study center personnel, as well as the sponsor personnel involved in the monitoring or conduct of the study, will be blinded to the study vaccine code.

The laboratories in charge of immunogenicity and safety testing will also be blinded so associating the sample with an assigned treatment or study visit will not be possible.

The treatment code should be broken only if the investigator/physician in charge of the participant feels that the case cannot be treated without knowing the identity of the study vaccine.

Except in cases of medical necessity, a participant's treatment should not be unblinded without the approval of the Sponsor. If unblinding should occur (by either accidental unblinding or emergency unblinding) before completion of the study, the investigator must promptly contact the Sponsor or designee and document the date and reason that the blind was broken in the source documentation and eCRF. Instructions regarding emergency unblinding will be provided to the investigator.

6.5 Vaccine Compliance

The investigator records all injections of investigational vaccine/control vaccine given to the participant.

The prescribed dosage, timing, and mode of administration may not be changed. Any departures from the intended regimen must be recorded in the eCRFs.

Administration of study vaccines will be performed at the study center by appropriately trained staff.

The clinical staff is responsible for the ongoing safety and wellbeing of the participants while they are in the study center. There is a physician on-site during normal business hours and medical advice is available by phone 24 hours a day. In addition, if necessary, the clinical staff can contact further on-call physicians or public emergencies services in the event of a serious medical event.

6.6 Treatment after the End of the Study

The Sponsor will not provide any additional care to participants after they leave the study.

CONFIDENTIAL

May not be used, divulged, published or otherwise disclosed without written consent of Arcturus Therapeutics, Inc.

7 PARTICIPANT WITHDRAWAL FROM THE STUDY

7.1 Discontinuation of Study Treatment

A "withdrawal" from the study vaccine refers to any participant who does not receive the complete treatment, i.e., when no further planned dose is administered from the date of withdrawal. A participant withdrawn from the study vaccine may continue further study procedures (safety or immunogenicity) if planned in the study protocol, as deemed appropriate by the investigator.

Primary reason relative to premature discontinuation of the study vaccine/control will be documented in the eCRF:

- AE(s);
- Participant decision, not due to an AE;
- If a female participant becomes pregnant (such participants will be followed up as described in Section 9.1.13 and Appendix 1);
- Lost to follow-up;
- Sponsor study termination;
- The participant tests positive for SARS-CoV-2 infection prior to Day 29 visit (prior to the second vaccination);
- Other (specify).

Contraindication for second vaccination are criteria for delay of the second vaccination are described in Section 5.3.

If a participant who does not meet enrollment criteria is inadvertently enrolled, that participant must be discontinued from study and the Sponsor or Sponsor designee must be contacted. An exception may be granted in rare circumstances for which there is a compelling safety reason to allow the participant to continue and the Investigator needs to document.

7.2 Participant Withdrawal from the Study

A participant may withdraw from the study at any time at his/her own request, or may be withdrawn, at any time at the discretion of the investigator for safety, behavioral, compliance, or administrative reasons.

If the participant withdraws consent for disclosure of future information, the Sponsor may retain and continue to use any data collected before such a withdrawal of consent.

If a participant withdraws from the study, he/she may request destruction of any samples taken and not tested, and the investigator must document this in the study center study records and inform the Sponsor's representative.

See the SoA (Table 1) for data to be collected at the time of study discontinuation and follow-up and for any further evaluations that need to be completed.

CONFIDENTIAL

May not be used, divulged, published or otherwise disclosed without written consent of Arcturus Therapeutics, Inc.

Primary reason relative to premature discontinuation from the study will be documented in the eCRF:

- AE(s).
- Participant decision, not due to an AE.
- If a female participant becomes pregnant (such participants will be followed up as described in Section 9.1.13 and Appendix 1).
- Lost to follow-up.
- Sponsor study termination.
- Other (specify).

7.3 Lost to Follow-up

A participant will be considered lost to follow-up if he or she repeatedly fails to return for scheduled visits and is unable to be contacted by the study center.

If a participant fails to return to the clinic for a required study visit, the study site must attempt to contact the participant and reschedule the missed visit as soon as possible and counsel the participant on the importance of maintaining the assigned visit schedule and ascertain whether or not the participant wishes to and/or should continue in the study.

Before a participant is deemed lost to follow-up, the investigator or designee must make every effort to regain contact with the participant (where possible, 3 telephone calls and, if necessary, a certified letter to the participant's last known mailing address or local equivalent methods). These contact attempts should be documented in the participant's medical record.

Should the participant continue to be unreachable, he/she will be considered to have withdrawn from the study.

CONFIDENTIAL

May not be used, divulged, published or otherwise disclosed without written consent of Arcturus Therapeutics, Inc.

8 STUDY ASSESSMENTS AND PROCEDURES

The following sections describe the study procedures and data to be collected. All procedures must be performed by qualified and trained staff.

The SoA is provided in Table 1.

8.1 Informed Consent

Informed consent must be obtained before any protocol-directed procedures are performed.

The requirements of the informed consent are described in Appendix 2.

8.2 Demographics

Demographic information to be collected about the participant will include age/date of birth (if applicable), sex, race (and ethnicity) and medical history, including presence of any medical condition associated with high risk of severe COVID-19.

The medical conditions that constitute a high risk for COVID-19 include cancer, cerebrovascular disease, chronic kidney disease, chronic lung diseases, chronic liver disease, diabetes mellitus type 1 and type 2, heart conditions (such as heart failure, coronary artery disease, or cardiomyopathies), mental health disorders, obesity (body mass index, or BMI ≥ 30 kg/m^2), pregnancy and recent pregnancy, smoking (current and former), tuberculosis, disabilities including Down syndrome, HIV, neurologic conditions limited to dementia, overweight (BMI ≥ 25 kg/m^2 but <30 kg/m^2), sickle cell disease, solid organ or blood stem cell transplantation, substance use disorders, use of corticosteroids or other immunosuppressive medications, cystic fibrosis, asthma, physical inactivity, and immune deficiencies (US CDC, 2023).

Medication history (including medications, blood products, and vaccines) is to be collected as prior and concomitant medications.

The use of concomitant medications does not require withdrawal of the participant from the study.

Antipyretics and/or analgesic medications taken within 24 hours prior to vaccination and the reason for their use (prophylaxis versus treatment) must be documented.

Prior medications should be collected as specified in Section 5.3.

Refer to Section 5.4 for details on prohibited medications.

This data must be written in the source documents.

8.3 Physical Examination and Vital Signs

Physical examinations must be performed by a qualified healthcare professional in accordance with local medical practice and regulations and as listed within the site responsibility delegation log.

“Qualified healthcare practitioner” refers to any licensed healthcare professional who is permitted by institutional policy to perform physical examinations.

CONFIDENTIAL

May not be used, divulged, published or otherwise disclosed without written consent of Arcturus Therapeutics, Inc.

A complete physical examination will be performed on Day 1 and may include the examination of the following: general appearance, weight, and height, abdomen; head and neck; eyes, ears, nose, throat, chest/respiratory, heart/cardiovascular, gastrointestinal/liver, musculoskeletal/extremities, dermatological/skin, thyroid, lymph nodes, and neurological. Symptom-directed physical examination may be performed at other visits if necessary.

Systolic/diastolic blood pressure, heart rate, respiratory rate, and body temperature will be measured. At vaccination visits, these assessments should be performed prior to and following study vaccination.

Vital signs should be measured in a seated position after the participant has rested comfortably for at least 5 minutes. Post vaccination, vital signs should be measured within the 30-minute observation period prior to the participant leaving the site.

This data will be written in the source document and any abnormal values or events must be documented in the eCRF AE form (if occurring after vaccination) or eCRF Medical History form (if occurring prior to vaccination). Treatment of any abnormality must be performed according to local medical practice and outside this study or by referral to an appropriate healthcare provider.

8.4 Pregnancy Testing

A urine pregnancy test will be conducted for women of childbearing potential prior to study vaccination (see Section 9.1.13). Blood pregnancy testing may be performed at the investigator's discretion or in accordance with institutional policy or local requirements. A positive pregnancy test will prevent study vaccination.

8.5 Rapid COVID-19 Antigen Test

A SARS-CoV-2 Rapid Antigen Test or equivalent will be performed on site prior to each study vaccination to confirm that a participant is eligible for vaccination. A positive test will prevent study vaccination.

8.6 ECG/Cardiac Enzymes Test

A baseline 12-lead ECG will be performed at recruitment on all study participants to identify potential asymptomatic myocarditis and pericarditis cases.

In case of suspected myocarditis or pericarditis cases during the study period, detailed evaluation, including ECG, cardiac enzyme testing, and other relevant data should be evaluated.

Additional ECG evaluation at unscheduled visits is at the investigator's discretion.

Blood samples for cardiac enzymes will be collected on Day 1 prior to vaccination (baseline) in all participants. The testing of baseline and post-vaccination samples for cardiac enzymes may be performed at the investigator's or Sponsor's discretion.

8.7 Inclusion and Exclusion Criteria

Only participants who have a signed ICF and satisfy all of the inclusion (Section 5.1) and exclusion (Section 5.2) criteria are eligible for randomization and to enter the study.

CONFIDENTIAL

May not be used, divulged, published or otherwise disclosed without written consent of Arcturus Therapeutics, Inc.

If the participant is ineligible for randomization, the investigator should record the primary reason for failure on the participant screening and enrollment log (Section 5.6).

8.8 Randomization

An interactive response technology (IRT) system will be used. Participants will be randomized in a 1:1:1 ratio to each study arm. Randomization will be stratified by COVID-19 vaccination history (such as a total number of COVID-19 vaccine doses received and the composition of the last booster dose (mRNA original strain or mRNA bivalent)). The randomization specification and schedule will be approved by the Sponsor's study statistician, or designee.

8.9 Vaccination

All participants will be monitored for 30 minutes after study injection for any immediate adverse reactions.

The instructions regarding study vaccine preparation and administration are provided in Section 6_Study_Treatment, the pharmacy manual and package insert document for control vaccine.

8.10 Immunogenicity Assessments

Humoral immune responses against SARS-CoV-2 will be assessed in Groups 1a and 1b, Groups 2a and 2b, and a set of samples from study ARCT-154-J01 using the following assays:

- Virus neutralizing assay (VNA, neutralizing antibodies against SARS-CoV-2) with Omicron XBB.1.5 subvariant;
- VNA (neutralizing antibodies against SARS-CoV-2 using various epidemiologically relevant SARS-CoV-2 variants)

Results from VNAs will be analyzed as:

- Geometric mean titer (GMT) on Days 1, 29 and 181;
- Geometric mean-fold rise (GMFR) as increases of the post-vaccination titer over the pre-vaccination titer;
- Seroconversion rate (SCR) on Day 29 as the percentage of participants in each arm with either:
  - A pre-vaccination titer below the lower limit of quantitation (LLOQ) and a postvaccination titer ≥4xLLOQ; or
  - A pre-vaccination titer ≥LLOQ and a ≥4-fold increase in post-vaccination titer.
- Proportion of participants with antibody titer ≥LLOQ.

Humoral immune responses against QIV will be assessed in Groups 1a and 1b, and Groups 3a and 3b using the following assays:

- Hemagglutination Inhibition (HI) assay against influenza vaccine strains. In case of lack of agglutination for a specific strain using HI assay, immunogenicity for that strain will be assessed as measured by microneutralization (MN) assay;
- MN titers against influenza vaccine strains.

CONFIDENTIAL

May not be used, divulged, published or otherwise disclosed without written consent of Arcturus Therapeutics, Inc.

The results of HI and MN will be analyzed as:

- Geometric mean titer (GMT) on Days 1 and 29;
- Geometric mean-fold ratio (GMFR) as increases of the post-vaccination titer over the pre-vaccination titer;
- SCR on Day 29 as the percentage of participants in a group with either:
  - A pre-vaccination titer below the LLOQ for the respective assay and a post-vaccination titer ≥4xLLOQ; or
  - A pre-vaccination titer ≥ LLOQ and a ≥4-fold increase in post-vaccination titer.
- Proportion of participants with HI titer ≥1:10, 1:20, 1:40, 1:80, 1:160 and 1:320 on Days 1 and 29;
- Proportion of participants with MN titer ≥≥1:10, 1:20, 1:40, 1:80, 1:160 and 1:320 on Days 1 and 29.

Planned time points for all immunogenicity assessments are provided in the SoA (Table 1).

8.10.1 Blood Volumes and Serological Testing

All analyses will be performed at a laboratory designated by the Sponsor or the Sponsor's designee using standardized and validated procedures.

The maximum volume of blood taken at any single visit is 30 mL, and the maximum total volume of blood collected during the study period in each study participant is approximately 90 mL. All samples listed in Table 6 will be drawn in all participants.

On Days 1 and 29, all samples from prespecified study arms will be tested using VNA with Omicron XBB.1.5 subvariant, and hemagglutination inhibition assay. A random subset of samples from Groups 2a and 2b will be tested using VNA to assess antibody persistence at Day 181 and to evaluate cross-neutralization of epidemiologically relevant variants of SARS-CoV-2 on Days 1 and 29. The random selection process of samples will be described in the Statistical Analysis Plan.

In case of lack of agglutination for a specific strain using the HI assay, a microneutralization assay will be used for the respective strain.

The testing plan is provided in Table 6.

Table 6 Testing Plan for ARCT-2303-01

Assay	Study Arms	Day 1<br>N	Day 29<br>N	Day 181<br>N
VNA (Omicron XBB.1.5)	Group 1a<br>Group 1b<br>Group 2a<br>Group 2b<br>ARCT-154-J01 study	400 (all)<br>160 (all)<br>400 (all)<br>160 (all)<br>~385	400 (all)<br>160 (all)<br>400 (all)<br>160 (all)<br>~385	—<br>—<br>100 (subset)<br>100 (subset)<br>—
Hemagglutination Inhibition assay (H1N1, H3N2, 2B)	Group 1a<br>Group 1b<br>Group 3a<br>Group 3b	400 (all)<br>160 (all)<br>400 (all)<br>160 (all)	400 (all)<br>160 (all)<br>400 (all)<br>160 (all)	—<br>—<br>—<br>—
Microneutralization assay (H1N1, H3N2, 2B)	Group 1a<br>Group 1b<br>Group 3a<br>Group 3b	In case of lack of agglutination for a specific strain using HI assay		—
VNA (various lineages, including any prevalent variant)	Group 2a	50 (subset)	50 (subset)	—
**Total maximum volume/ each participant**		**30 mL**	**30 mL**	**30 mL**

Abbreviations: N: number of participants per timepoint; VNA, virus neutralizing assay; Safety testing may be performed in case of suspected myocarditis or pericarditis or other safety concerns

8.10.2 Biological Sample Retention and Destruction

Details of biological sample collection, handling, and analysis are presented in the laboratory manual. Repeat or unscheduled samples may be collected for safety reasons or for technical issues with the samples. Biological samples will be preserved and retained at a central laboratory that was contracted by the Sponsor for this purpose for up to but not longer than 20 years or as required by applicable law. The Sponsor has put into place a system to protect the participant's personal information to ensure optimal confidentiality and defined standard processes for sample and data collection, storage, analysis, and destruction.

8.11 Safety Assessments

All participants will be monitored for 30 minutes after study injection for any immediate AEs with appropriate medical treatment readily available in case it is needed.

- Local reactions and systemic solicited AEs (Section 9.1.9) will be recorded daily within 7 days after each study vaccination:
  - Local reactions: pain, erythema, and swelling at the injection site;
  - Systemic AEs: fatigue, headache, myalgia, arthralgia, nausea, dizziness, chills, and fever.
- Any unsolicited AEs will be collected within 28 days after each study vaccination (Section 9.1.9);
- Pregnancies will be recorded throughout the study (Section 9.1.13);

- Any SAEs (Section 9.1.2), AESIs (Section 9.1.14), MAAEs (Section 9.1.15) and AEs leading to early study termination, will be reported during the entire study period. These data will be captured by interviewing the participant during the site visits and phone calls and by review of available medical records.
- Any diagnosed episodes of SARS-CoV-2 infection will be reported as MAAEs; no active surveillance for SARS-CoV-2 infection is planned in this study.

Refer to Section 9.0 for safety definitions. This section contains details on collection (Section 9.1.7), follow-up (Section 9.1.10) and reporting of AEs (Section 9.1.11).

Planned time points for all safety assessments are provided in the SoA (Table 1).

8.12 Schedule of Assessments

8.12.1 Screening (Day 1)

All screening evaluations must be completed and reviewed to confirm that potential participants meet all eligibility criteria. The investigator will maintain a screening log to record details of all participants screened and to confirm eligibility or record reasons for screening failure, as applicable.

If all Screening procedures are performed prior to Day 1, the following Screening assessments should be repeated prior to dosing on Day 1: physical examination, vital signs, pregnancy testing, and testing for COVID-19.

8.12.2 Pre-vaccination Procedures (Day 1 and Day 29)

- Informed consent (Section 8.1)*
- Demographics (Section 8.2)*
- Medical history and prior medication (Section 8.2)*
- Concomitant medications (Section 5.4)
- Physical examination and vital signs (Section 8.3)
- 12-lead electrocardiogram* and blood samples for cardiac enzymes testing* (Section 8.6)
- Pregnancy test (Section 8.4)
- Rapid COVID-19 antigen test or equivalent (Section 8.5)
- Blood sampling for immunogenicity (Section 8.10)

* Pre-vaccination procedure for Day 1 vaccination only.

8.12.3 Vaccination and Post-vaccination Procedures (Day 1 and Day 29)

Perform randomization (Section 8.8) at Visit 1 (Day 1).

After confirming eligibility (Section 8.7) at Visit 1 (Day 1) and at subsequent vaccination visit (Day 29), ensure that the participant does not meet criteria for delay or discontinuation (Section 5.3).

Perform vaccination (Section 8.9).

CONFIDENTIAL

May not be used, divulged, published or otherwise disclosed without written consent of Arcturus Therapeutics, Inc.

The following post-vaccination procedures will be performed at Visit 1 (Day 1) and Visit 2 (Day 29):

- Participants will be observed for 30 minutes after vaccination in case of any adverse reaction. Any AEs reported within 30 minutes after vaccination will be recorded in the eCRF.
- After study vaccine administration, vital signs will be measured (BP, RR, body temperature, and heart rate) and vital sign measurement should be repeated if clinically indicated.

Careful training of the participant (or the individual who will be performing the measurements) on how to measure local reactions and body temperature, how to complete and how often to complete the eDiary will occur at Visit 1.

At Day 1, when two study vaccines will be co-administered, participant will be instructed to record local reactogenicity for both arms separately.

Specifically, participants in:

Groups 1a and 1b will receive: ARCT-2303 on the left arm and influenza vaccine on the right arm,

Groups 2a and 2b will receive: ARCT-2303 on the left arm and placebo on the right arm,

Groups 3a and 3b will receive: placebo on the left arm and influenza vaccine on the right arm.

At Day 29, vaccine administration will be done in the non-dominant arm.

eDiary instructions must include the following:

- The participant must understand that timely completion of the eDiary on a daily basis is a critical component to study participation.
- The participant should be instructed on how to complete the eDiary and how to make corrections to erroneous entries.
- Starting on the day of vaccination, the participant will check approximately 6 hours post-vaccination (minimum of 4 hours post-vaccination) for specific types of reactions at the injection site (solicited local adverse reactions), any specific generalized symptoms (solicited systemic AEs), body temperature (preferably axillary), any other symptoms or change in the participant's health status, and any medications taken.
- The participant must use the thermometer provided by the site for body temperature measurement. If the participant feels unusually hot or cold during the day, the participant should check body temperature several times and record the highest body temperature observed that day on the eDiary.
- The collection of body temperature, solicited local adverse reactions, and solicited systemic AEs will continue for a total of 7 days on the eDiary.

The eDiary will be the only source document allowed for solicited local reactions and systemic AEs (including body temperature measurements).

May not be used, divulged, published or otherwise disclosed without written consent of Arcturus Therapeutics, Inc.

Note: Any solicited AE that meets any of the following criteria must be entered into participants' source document (see Appendix 2) and also as an AE on the AE CRF:

- Solicited local or systemic AEs leading to a visit to a healthcare provider (MAAE, Section 9.1.15).
- Solicited local or systemic AEs leading to the participant withdrawing from the study or the participant being withdrawn from the study by the investigator (AE leading to withdrawal; see Section 7.1).
- Solicited local or systemic AE that otherwise meets the definition of an SAE (Section 9.1.2).
- Solicited local or systemic AE that continues beyond 7 days post-vaccination.

Compliance checks for eDiary completion will be performed on Day 2 (±1) and Day 5 (±1) after each vaccination. The site will perform a check to verify that the participant has completed the eDiary. Participants will be contacted by telephone if the eDiary is incomplete. Compliance checks will be documented in the source.

8.12.4 Non-vaccination Site Visit Procedure (Day 57)

Site visit that does NOT include a vaccination will be performed on Day 57. The following procedures will be completed as planned in the SoA (Table 1):

- Symptom-directed physical examination and vital signs
- Review of eDiary
- Adverse events collection
- Concomitant medications/vaccines collection

The site should schedule the next site visit or other study activity with the participant.

8.12.5 Phone Contact (Day 91)

A safety call will be made by phone contact (PC) on Day 91 for collection of any signs and symptoms the participant may have experienced, any new diagnosed medical conditions, and medications taken since the previous contact. This call will follow a script and will be made by trained healthcare personnel.

A trained healthcare professional is defined as any healthcare professional permitted by institutional policy and trained to perform delegated tasks, trained on the study procedure(s), and identified within the site signature and delegation log.

Any safety information collected during the phone contact will be entered into the source documents, and any medically attended AEs, SAEs, AESIs, and AEs leading to early study termination and related medications and any vaccinations will be reported in the eCRF, as appropriate.

8.12.6 End-of-study (Day 181) or Early Termination (ET)/Last Visit Procedures

The final (end-of-study) contact will be performed on Day 181. If a participant terminates earlier, the ET visit procedures should be performed at their last study visit, if possible. See the SoA (Table 1) for data to be collected at the end-of-study and an early termination visit. The

CONFIDENTIAL

May not be used, divulged, published or otherwise disclosed without written consent of Arcturus Therapeutics, Inc.

investigator must complete the end-of-study CRF page for all participants who received the study vaccine.

8.12.7 Unscheduled Visits

Unscheduled visits include visits for possible COVID-19 episodes, possible cardiac events, or evaluation of any adverse event of concern. These visits may be performed in the clinic, by home visit, by hospital visit (if allowed by local policy), or via telemedicine/telephone visit. Required procedures at these visits include AE and concomitant medication collection. Should the visit occur in person, the visit should also include evaluation of vital signs, and symptom-directed physical examination.

For participants with suspected SARS-CoV-2 infection, nasal or nasopharyngeal samples may be collected for RT-PCR testing.

For participants evaluated for cardiac symptoms, 12-lead electrocardiogram and targeted safety laboratory assessments should be performed. If myocarditis or pericarditis is suspected based on investigator evaluation of the participant, the participant should be referred to a cardiologist, ideally within 24 hours (see Section 9.1.14.1).

A blood sample might be drawn for additional laboratory safety evaluation, including cardiac enzymes, as per investigator discretion.

Additional safety phone calls may be performed as a follow-up for the evaluation of adverse event(s) or SARS-CoV-2 infection.

8.12.8 Post-study Procedures

Any SAE that occurs outside of the protocol-specified observation period or after the end of the study but is considered to be caused by the study vaccine will be processed by the Sponsor (or delegate) and must be reported to the Sponsor.

Any post-study pregnancy-related SAE considered reasonably related to the study vaccine by the investigator will be reported to the Sponsor. While the investigator is not obligated to actively seek this information in former participants, he or she may learn of an SAE through routine medical practice.

These SAEs and pregnancy information will be considered as part of the spontaneous reporting towards the investigational study vaccine in order to ensure the safety of all participants.

No post-study care will be provided.

CONFIDENTIAL

May not be used, divulged, published or otherwise disclosed without written consent of Arcturus Therapeutics, Inc.

9 ADVERSE EVENTS

The following sections provide definitions for AEs, the procedures for collecting, following up and reporting to the authorities.

AEs will be reported by the participant (or, when appropriate, by a caregiver) or identified at study visit.

The investigator and any designees are responsible for detecting, documenting, and recording events that meet the definition of an AE or SAE and remain responsible for following up AEs that are serious, considered related to the study vaccine or study procedures, or that caused the participant to discontinue the study (see Section 7).

9.1 Definitions

9.1.1 Definition of an Adverse Event

- An AE is any untoward medical occurrence in a participant or participant administered a medicinal product, whether or not considered related to the study vaccine.
- NOTE: An AE can therefore be any unfavorable and unintended sign (including an abnormal laboratory finding), symptom, or disease (new or exacerbated) temporally associated with the use of a medicinal product.

9.1.1.1 Events Meeting the AE Definition

- Any abnormal laboratory test results (hematology, clinical chemistry, or urinalysis) or other safety assessments (e.g., electrocardiogram, radiological scans, or vital signs measurements), including those that worsen from baseline, considered clinically significant in the medical and scientific judgment of the investigator.
- Exacerbation of a chronic or intermittent pre-existing condition including either an increase in frequency and/or intensity of the condition.
- New conditions detected or diagnosed after study vaccine administration even though it may have been present before the start of the study.
- Signs, symptoms, or the clinical sequelae of a suspected drug-drug interaction.

9.1.1.2 Events NOT Meeting the AE Definition

- Any clinically significant abnormal laboratory findings or other abnormal safety assessments which are associated with the underlying disease, unless judged by the Investigator to be more severe than expected for the participant's condition.
- Medical or surgical procedure (e.g., endoscopy, appendectomy): The condition that leads to the procedure is the AE.
- Situations in which an untoward medical occurrence did not occur (social and/or convenience admission to a hospital).
- Anticipated day-to-day fluctuations of pre-existing disease(s) or condition(s) present or detected at the start of the study that do not worsen.

May not be used, divulged, published or otherwise disclosed without written consent of Arcturus Therapeutics, Inc.

- Pre-existing conditions or signs and/or symptoms present in a participant prior to the first study vaccination. These events will be recorded in the medical history section of the eCRF.

9.1.2 Definition of an SAE

An SAE is defined as any AE that, at any dose:

a) Results in death.

b) Is life-threatening.

The term 'life-threatening' in the definition of "serious" refers to an event in which the participant was at risk of death at the time of the event. It does not refer to an event, which hypothetically might have caused death, if it were more severe.

c) Requires inpatient hospitalization or prolongation of existing hospitalization.

In general, hospitalization signifies that the participant has been detained (usually involving at least an overnight stay) at the hospital or emergency ward for observation and/or treatment that would not have been appropriate in the physician's office or outpatient setting. Complications that occur during hospitalization are AEs. If a complication prolongs hospitalization or fulfills any other serious criteria, the event is serious. When in doubt as to whether "hospitalization" occurred or was necessary, the AE should be considered serious.

Hospitalization for elective treatment of a pre-existing condition that did not worsen from baseline is not considered an AE.

d) Results in persistent disability/incapacity.

- The term disability means a substantial disruption of a person's ability to conduct normal life functions.
- This definition is not intended to include experiences of relatively minor medical significance such as uncomplicated headache, nausea, vomiting, diarrhea, influenza, and accidental trauma (e.g., sprained ankle) which may interfere with or prevent everyday life functions but do not constitute a substantial disruption.

e) Is a congenital anomaly/birth defect.

f) Is a medically important event:

- Medical or scientific judgment should be exercised in deciding whether SAE reporting is appropriate in other situations such as important medical events that may not be immediately life-threatening or result in death or hospitalization but may jeopardize the participant or may require medical or surgical intervention to prevent one of the other outcomes listed in the above definition. These events should usually be considered serious.
- Examples of such events include invasive or malignant cancers, intensive treatment in an emergency room or at home for allergic bronchospasm, blood dyscrasias, or convulsions that do not result in hospitalization, or development of drug dependency or drug abuse.

CONFIDENTIAL

May not be used, divulged, published or otherwise disclosed without written consent of Arcturus Therapeutics, Inc.

9.1.3 Recording of AEs and SAEs

- When an AE occurs, it is the responsibility of the investigator to review all documentation (e.g., hospital progress notes, laboratory reports, and diagnostics reports) related to the event.
- The investigator will then record all relevant AE information in the eCRF. Each event must be recorded separately.
- It is not acceptable for the investigator to send photocopies of the participant's medical records to the Sponsor/Sponsor's designee in lieu of completion of the relevant eCRF page.
- There may be instances when copies of medical records for certain cases are requested by the Sponsor/Sponsor's designee. In this case, all participant identifiers, with the exception of the participant number, will be redacted on the copies of the medical records before submission to the Sponsor/Sponsor's designee.
- The investigator will attempt to establish a diagnosis of the event based on signs, symptoms, and/or other clinical information. Whenever possible, the diagnosis (not the individual signs/symptoms) will be documented as the AE.

9.1.4 Assessment of Intensity

The investigator will make an assessment of intensity for each AE reported during the study and assign it to 1 of the following categories, based on the US Food and Drug Administration (FDA) Toxicity Grading Scale for Healthy Adult and Adolescent Volunteers Enrolled in Preventive Vaccine Clinical Trials:

- Mild (Grade 1): transient with no limitation in normal daily activity
- Moderate (Grade 2): some limitation in normal daily activity
- Severe (Grade 3): unable to perform normal daily activity
- Potentially Life-Threatening (Grade 4): an event in which the participant was at immediate risk of death at the time of the event
- Fatal (Grade 5): an event with a fatal outcome

Note: An event is defined as 'serious' when it meets at least 1 of the predefined outcomes as described in the definition of an SAE, NOT when it is rated as severe. Severe is a category utilized for rating the intensity of an event; and both AEs and SAEs can be assessed as severe.

9.1.5 Assessment of Causality

- The investigator is obligated to assess the relationship between study vaccine and each occurrence of each AE. The AE must be characterized as related or unrelated:

– **Related:** An AE that followed a temporal sequence from administration of a drug (including the course after withdrawal of the drug), or for which possible involvement of the drug could not be ruled out, although factors other than the drug, such as underlying diseases, concurrent medical conditions, concomitant drugs, and concurrent treatments, were also responsible.

May not be used, divulged, published or otherwise disclosed without written consent of Arcturus Therapeutics, Inc.

- **Unrelated:** An AE that did not follow a temporal sequence from study administration of a drug and/or that could reasonably be explained by other factors, such as underlying diseases, concurrent medical conditions, concomitant drugs, and concurrent treatments.

- The investigator will use clinical judgment to determine the relationship.
- Alternative causes, such as underlying disease(s), concomitant therapy, and other risk factors, as well as the temporal relationship of the event to study vaccine administration will be considered and investigated.
- The investigator will also consult the IB and product information, for marketed products, in his/her assessment.
- For each AE/SAE, the investigator must document in the medical notes that he/she has reviewed the AE/SAE and has provided an assessment of causality.
- There may be situations in which an SAE has occurred and the investigator has minimal information to include in the initial report to the Sponsor/Sponsor's designee. However, it is very important that the investigator always make an assessment of causality for every event before the initial transmission of the SAE data to the Sponsor/Sponsor's designee.
- The investigator may change his/her opinion of causality in light of follow-up information and send a SAE follow-up report with the updated causality assessment.
- The causality assessment is one of the criteria used when determining regulatory reporting requirements.

9.1.6 Assessment of Outcome of AE

The investigator will assess the outcome of all AEs recorded during the study as:

- Recovered/resolved.
- Recovering/resolving.
- Not yet recovered/not yet resolved.
- Recovered with sequelae/resolved with sequelae.
- Fatal (all fatal events are considered SAEs and should be reported accordingly).
- Unknown.

9.1.7 Time Period and Frequency for Collecting Adverse Event and Serious Adverse Event Information

Medical occurrences that begin before the study vaccination but after obtaining informed consent will be recorded in the medical history/current medical conditions section of the eCRF, not the AE section. Exacerbation of a chronic or intermittent pre-existing condition including either an increase in frequency and/or intensity of the condition after the first vaccination will be considered an AE.

For all participants:

- Any unsolicited AEs will be collected within 28 days after each vaccination.
- Any AEs that occur within 30 minutes after study vaccination are to be reported in the eCRF.
- Any AEs that are determined by the investigator (or designee) to be SAEs, AESIs (serious and nonserious), MAAEs, or AEs leading to early termination from the study or

May not be used, divulged, published or otherwise disclosed without written consent of Arcturus Therapeutics, Inc.

study vaccination (regardless of the causal relationship) are to be reported in the eCRF from the moment of first vaccination until completion of the participant's last study-related procedure, which may include contact for safety follow-up (until the end of the study).

All AESIs and SAEs will be recorded and reported to the Sponsor or the Sponsor's designee immediately and under no circumstance should this exceed 24 hours after the investigator becomes aware of it. The investigator will submit any updated data to the Sponsor within 24 hours of it being available.

9.1.8 Method of Detecting AEs and SAEs

The method of recording, evaluating, and assessing intensity, causality and outcome of AEs and SAEs and the procedures for completing and transmitting AESI/SAE reports are provided in Section 9.1.7, Section 9.1.11 and Section 9.1.14.

Care will be taken not to introduce bias when detecting AEs and/or SAEs. Open-ended and non-leading verbal questioning of the participant is the preferred method to inquire about AE occurrences.

9.1.9 Solicited and Unsolicited Adverse Events

The investigator or designee will review and verify the completed eDiary during discussions with the participant as per the time points detailed in the SoA.

Participants will record solicited AEs and a presence of unsolicited AEs in the eDiary. The investigator or designee will review the eDiary entries and collect the details of the unsolicited AEs with the participant. The investigator or designee will then transcribe the appropriate collected information into the relevant eCRF pages, according to the guidance presented in Section 9.1.7. If the AE meets AESI criteria or SAE criteria, it should also be reported to the Sponsor or Sponsor's designee (Section 9.1.11).

Solicited local (injection site) reactions:

- Injection site pain
- Erythema
- Swelling

Solicited systemic AEs:

- Fatigue
- Headache
- Myalgia
- Arthralgia
- Nausea
- Dizziness
- Chills
- Fever (≥38.0°C; axillary)

Severity grading for these AEs is presented in Table 7.

CONFIDENTIAL

May not be used, divulged, published or otherwise disclosed without written consent of Arcturus Therapeutics, Inc.

Table 7 Severity Grading for Solicited Local Reactions and Systemic Adverse Events

	Mild	Moderate	Severe
Pain	No interference with daily activities	Interferes with daily activities	Prevents daily activity
Erythema	25-50 mm	51-100 mm	> 100 mm
Swelling	25-50 mm	51-100 mm	> 100 mm
Fatigue	No interference with daily activities	Interferes with daily activities	Prevents daily activity
Headache	No interference with daily activities	Interferes with daily activities	Prevents daily activity
Myalgia	No interference with daily activities	Interferes with daily activities	Prevents daily activity
Arthralgia	No interference with daily activities	Interferes with daily activities	Prevents daily activity
Nausea	No interference with daily activities	Interferes with daily activities	Prevents daily activity
Dizziness	No interference with daily activities	Interferes with daily activities	Prevents daily activity
Chills	No interference with daily activities	Interferes with daily activities	Prevents daily activity
Fever (°C)	38.0-38.4	38.5-38.9	≥39.0

Note: Based on Food and Drug Administration (FDA) Guidance Document: Toxicity Grading Scale for Healthy Adult and Adolescent Volunteers Enrolled in Preventive Vaccine Clinical Trials.

Unsolicited AEs

An unsolicited AE is an AE that is not listed as 'solicited' above.

Potential unsolicited AEs may be medically attended (defined as symptoms or illnesses requiring hospitalization, or emergency room visit, or visit to/by a healthcare provider; Section 9.1.15) or were of concern to the participants. In case of such events, participants will be instructed to contact the study center as soon as possible to report the event(s). The detailed information about the reported unsolicited AEs will be collected by the qualified study center personnel during the interview and will be documented in the participant's records.

Unsolicited AEs that are not medically attended or perceived as a concern by participants will be collected during planned safety visits/contacts with the participants and by review of available medical records.

9.1.10 Follow-up of AEs and SAEs

After the initial AE/SAE report, the investigator is required to proactively follow each participant at subsequent visits/contacts. All AEs/SAEs will be followed until resolution, stabilization, the event is otherwise explained, or the participant is lost to follow-up (as defined in Section 7.2). Further information on follow-up procedures is given in Section 9.1.10.

- The investigator is obligated to perform or arrange for the conduct of supplemental measurements and/or evaluations as medically indicated or as requested by the Sponsor/Sponsor's designee to elucidate the nature and/or causality of the AE as fully as possible. This may include additional laboratory tests or investigations, histopathological examinations, or consultation with other healthcare professionals.
- New or updated information will be recorded in the originally completed eCRF.
- The investigator will submit any updated SAE data to the Sponsor/Sponsor's designee within 24 hours of receipt of the information.
- A post-study AE/SAE is defined as any event that occurs outside of the AE/SAE reporting period. Investigators are not obligated to actively seek AEs or SAEs in former

May not be used, divulged, published or otherwise disclosed without written consent of Arcturus Therapeutics, Inc.

study participants; however, if the investigator learns of any SAE, including a death, at any time after a participant has been discharged from the study, and he/she considers the event to be reasonably related to the study vaccine(s), the investigator will promptly notify the Sponsor.

- Serious adverse events and adverse events of special interest still ongoing at the end of study must be followed up until resolution or stabilization, or until the event outcome is provided. Reporting may continue after the follow-up visit and database lock.

9.1.11 Regulatory Reporting Requirements for SAEs

Prompt notification by the investigator to the Sponsor of an SAE is essential so that legal obligations and ethical responsibilities towards the safety of participants and the safety of a study vaccine under clinical investigation are met.

The Sponsor has a legal responsibility to notify both the local regulatory authority and other regulatory agencies about the safety of study vaccine under clinical investigation. The Sponsor will comply with country-specific regulatory requirements relating to safety reporting to the regulatory authority, institutional review board/independent ethics committee (IRB/IEC), and investigators.

Investigator safety reports must be prepared for suspected unexpected serious adverse reactions (SUSARs) according to local regulatory requirements and Sponsor policy and forwarded to investigators as necessary.

An investigator who receives an investigator safety report describing an SAE or other specific safety information (e.g., summary or listing of SAEs) from the Sponsor will review and then file it along with the IB and will notify the IRB/IEC, if appropriate according to local requirements.

9.1.12 Reporting of AESIs and SAEs

AESI and SAE reporting can be made via electronic data capture (EDC; Section 9.1.12.1) or paper AESI/SAE form (Section 9.1.12.2).

9.1.12.1 AESI and SAE Reporting via the eCRF Page in EDC

- The primary mechanism for reporting an AESI or SAE to the Sponsor/Sponsor's designee will be using the electronic data collection tool (AE eCRF page in EDC).
- If the electronic system is unavailable for more than 24 hours, then the study center will use the paper AESI/SAE Report Form (see Section 9.1.12.2) as a back-up mode of reporting. The study center will enter the AESI/SAE data back into the electronic system as soon as it becomes available. All initial and follow-up information should be reported within 24 hours of awareness.

9.1.12.2 AESI and SAE Reporting via Paper AESI/SAE Report Form

- If the EDC is not available, AESI/SAEs can be reported to the Sponsor/Sponsor's designee using a paper AESI/SAE Report Form.
- All initial and follow-up information should still be reported within 24 hours of awareness.

May not be used, divulged, published or otherwise disclosed without written consent of Arcturus Therapeutics, Inc.

- The contacts (email/fax) for AESI/SAE paper reporting can be found in the instructions of the paper AESI/SAE Report Form.

9.1.13 Pregnancy

Female participants of childbearing potential are to have a urine or blood pregnancy test (at investigator discretion or per local requirements) prior to the study vaccine administration. The study vaccine may only be administered if the pregnancy test is negative.

Note: Pregnancy tests must be performed even if the participant is menstruating at the time of the study visit. Any female participant who becomes pregnant while participating in the study will discontinue the study vaccine. The participant will be encouraged to remain in the study for safety follow-up until the end of the study.

Details of pregnancies in female participants, for which the date of conception is within the entire study period, should be followed for pregnancy outcomes. Information on delivery and newborn should be reported to the Sponsor within 1 month of the end of the pregnancy. Information about newborn health condition may be collected at 6 or 12 months after birth.

Details of pregnancies in female partners of male participants will be collected according to local regulations.

If a pregnancy is reported, the investigator should inform the Sponsor within 24 hours of learning of the pregnancy and should follow the procedures outlined in Appendix 1.

Abnormal pregnancy outcomes (e.g., spontaneous abortion, fetal death, stillbirth, congenital anomalies, ectopic pregnancy) are considered SAEs.

9.1.14 Adverse Events of Special Interest

AEs potentially associated with COVID-19 and COVID-19 vaccines should be reported as AEs of special interest (AESIs). These events were defined by the Safety Platform for Emergency vACcines (SPEAC Recommendations, 2020) and are shown in Appendix 3 (Table 13).

Once any AESI is diagnosed (serious or nonserious), the investigator (or designate) must record the AESI on the relevant eCRF page and report the AESI in an expedited manner, as per Section 9.1.11.

When there is enough evidence to make any of the above diagnoses, the AE must be reported as an AESI. Symptoms, signs, or conditions which might (or might not) represent the above diagnoses should be recorded and reported as AEs but not as AESIs until the final or definitive diagnosis has been determined, and alternative diagnoses have been eliminated or shown to be less likely.

The DSMB may adjudicate reported AEs that meet the criteria of AESIs.

CONFIDENTIAL

May not be used, divulged, published or otherwise disclosed without written consent of Arcturus Therapeutics, Inc.

9.1.14.1 Suspected Myocarditis and Pericarditis Cases

All suspected cases of myocarditis, pericarditis, and myopericarditis should be reported to the Sponsor in an expedited manner.

All study participants should be instructed to report to site personnel, as soon as possible, a new onset of chest pain, shortness of breath (at rest or during activity), rapid or irregular heartbeat (arrhythmias), swelling of the legs, ankles, and feet, light-headedness, collapse, and fatigue occurring within 6 weeks after each vaccination.

The investigator must perform the initial assessment of the study participant with these symptoms/signs and refer individuals with suspected myocarditis and pericarditis to a cardiologist for evaluation and management, ideally within 24 hours of assessment by site. A questionnaire will be provided to the investigators for assessment of suspected cases. A 12-lead ECG and cardiac enzyme testing should be performed in all participants with suspected myocarditis and pericarditis. Additional evaluations (such as echocardiography, cardiac MRI) might be performed based on the cardiologist's recommendations.

A central cardiac adjudication committee will assess all suspected cases of myocarditis and pericarditis (as determined by investigator and cardiologist) according to the Brighton Collaboration case definition (Sexson Tejtel et al, 2021) within 1 week of the initial reporting.

Cases of myocarditis and/or pericarditis will be followed until resolution of abnormal test findings and symptoms.

Cases of myocarditis and/or pericarditis occurring in temporal association with vaccination will be considered potentially related, unexpected, and serious, and are subject to expedited reporting requirements as outlined in Section 9.1.11.

9.1.15 Medically Attended Adverse Events

9.2 Treatment of Overdose

Not applicable.

9.3 Genetics

Genetics are not evaluated in this study.

10 STATISTICAL CONSIDERATIONS

The statistical analyses for the coprimary immunogenicity objectives (1, 2, 3, and 4) will be performed and tested in 2 steps.

The first step will be testing coprimary objectives 1 and 2:

1 – superiority for the Omicron XBB.1.5 subvariant on Day 29 for GMT ratio.

2 – noninferiority for the Omicron XBB.1.5 subvariant on Day 29 for the difference of SCR.

The success criterion of the study is that the coprimary objectives 1 and 2 (Table 4) are demonstrated. If the coprimary objectives 1 and 2 (based on GMT ratio and difference of SCRs) are both met, then the testing will continue to the second step sequentially.

The second step will be testing coprimary objectives 3 and 4 by assessing the noninferiority of immune response after co-administration and standalone administration of ARCT-2303 and Flucelvax Quadrivalent.

The success criterion for the co-administration part of the study is that the coprimary objectives 3 (noninferiority for all 4 influenza strains) and 4 (noninferiority for XBB.1.5 variant) are demonstrated.

If all coprimary objectives are met, an additional step of sequential testing of the secondary objective 2 (superiority based on SCR) followed by the testing of the secondary objective 1 (‘super superiority’ for GMT ratio) will be performed.

No adjustment for multiplicity is planned for this study due to the hierarchical testing strategy.

10.1 Statistical Hypotheses and Hypothesis Testing

Step 1:

Coprimary immunogenicity objectives 1 and 2 (Table 4), evaluating ARCT-2303 as compared to the ARCT-154 (study ARCT-154-J01) for the Omicron XBB.1.5 subvariant.

1) For the coprimary immunogenicity objective 1 evaluating the simple superiority of ARCT-2303 (booster) as compared to ARCT-154 (booster) in terms of the neutralizing immune response against the Omicron XBB.1.5 subvariant, the following null hypothesis will be applied and evaluated in the PP population:

H_{01}: Day 29 GMT ARCT-2303 $_{\text{booster dose}}$ /Day 29 GMT ARCT-154$_{\text{booster dose}}$ ≤ 1.0

2) For the coprimary immunogenicity objective 2 evaluating the noninferiority of ARCT-2303 (booster) as compared to ARCT-154 (booster) in terms of the neutralizing immune response against the Omicron XBB.1.5 subvariant, the following null hypothesis will be applied and evaluated in the PP population:

H_{02}: Day 29 SCR ARCT-2303 $_{\text{booster dose}}$ minus Day 29 SCR ARCT-154$_{\text{booster dose}}$ $\leq -5.0\%$

Two-sided $\alpha = 0.05$ will be used for all tests.

CONFIDENTIAL

May not be used, divulged, published or otherwise disclosed without written consent of Arcturus Therapeutics, Inc.

Step 2:

If objectives in step 1 are met, then the noninferiority of immune response after co-administration and standalone administration of ARCT-2303 and Flucelvax Quadrivalent will be compared (coprimary objectives 3 and 4; Table 4), and the following null hypotheses will be applied and evaluated in the PP population:

H_{03}: Day 29 GMT QIV $_{\text{QIV+ARCT-2303}}$ /Day 29 GMT QIV $_{\text{QIV+Placebo}}$ $\leq$ 0.67 (Group 1a vs 3a)

H_{04}: Day 29 GMT ARCT-2303 $_{\text{QIV+ARCT-2303}}$ /Day 29 GMT ARCT-2303$_{\text{ARCT-2303+Placebo}}$ $\leq$ 0.67 (Group 1a vs 2a)

No adjustment for multiplicity is planned, as both H_{03} and H_{04} (all 5 null hypotheses: 4 strains for QIV and 1 strain for ARCT-2303) must be rejected to declare noninferiority of immune response.

Step 3:

If coprimary objectives 1, 2, 3 and 4 are met, then secondary objectives 2 (superiority of SCR) and 1 (‘super superiority’ of GMTs) of the ARCT-2303 vaccine, when given as a booster dose compared to a booster dose of ARCT-154 (in study ARCT-154-J01), will be tested in a sequential manner as follows (if H_{05} is rejected then H_{06} is tested too):

H_{05}: Day 29 SCR ARCT-2303 $_{\text{booster dose}}$ minus Day 29 SCR ARCT-154$_{\text{booster}}$ $\leq$ 0%

H_{06}: Day 29 GMT ARCT-2303 $_{\text{booster dose}}$ /Day 29 GMT ARCT-154$_{\text{booster}}$ $\leq$ 1.5

The ratio of GMTs and associated two-sided 95% confidence interval (CI) between Day 29 ARCT-2303 and Day 29 ARCT-154 will be calculated as the exponentiation of the difference of the two means of the logarithmically transformed assay results of the two groups and associated two-sided 95% CI. Analysis of covariance (ANCOVA) will be used to calculate the logarithmical mean and confidence interval with covariate and factor adjustment (further details will be described on the statistical analysis plan).

Simple superiority of ARCT-2303 to ARCT-154 in terms of neutralizing antibodies against Omicron XBB.1.5 subvariant will be demonstrated if the lower limit of the 2-sided 95% CI for GMT ratios (ARCT-2303/ARCT-154) is higher than the specified limit (1.0).

Noninferiority of ARCT-2303 to ARCT-154 in terms of neutralizing antibodies (SCRs) against Omicron XBB.1.5 subvariant will be demonstrated if the lower limit of the 2-sided 95% CI for the difference of SCRs (ARCT-2303 minus ARCT-154) is higher than the specified limit (-5%).

Similarly, Day 29 ratio of GMTs and associated two-sided 95% confidence interval for ARCT-2303 and Flucelvax Quadrivalent (co-administration compared to standalone) will be calculated as the exponentiation of the difference of the two means of the logarithmically transformed assay results of the two groups and associated two-sided 95% CI. Noninferiority of ARCT-2303 and Flucelvax Quadrivalent (co-administration versus standalone) will be demonstrated if the lower limit of the 2-sided 95% CI for GMT ratios (Group 1a vs Group 3a and Group 1a vs Group 2a) is higher than the specified limit (0.67).

CONFIDENTIAL

May not be used, divulged, published or otherwise disclosed without written consent of Arcturus Therapeutics, Inc.

Super superiority of ARCT-2303 to ARCT-154 in terms of neutralizing antibodies GMTs against Omicron XBB.1.5 subvariant will be demonstrated (secondary objective) if the lower limit of the 2-sided 95% CI for GMT ratios (ARCT-2303/ARCT-154) is higher than the specified limit (1.5).

Superiority of ARCT-2303 to ARCT-154 in terms of neutralizing antibodies SCR against Omicron XBB.1.5 subvariant will be demonstrated (secondary objective) if the lower limit of the 2-sided 95% CI for difference of SCRs (ARCT-2303 minus ARCT-154) is higher than the specified limit (0%).

There is no formal hypothesis testing for evaluating the co-administration of ARCT-2303 and Fluad Quadrivalent. The results of the analysis will be presented descriptively.

There is no formal hypothesis testing for the evaluation of the safety objective. Safety will be summarized descriptively within the Safety Analysis Set population.

10.2 Sample Size Determination

A total sample size of approximately 1680 participants (1200 participants 18 to 64 years of age, and 480 participants ≥65 years of age), and a sample size of 385 participants of study ARCT-154-J01 (PPS-1) is proposed for the following study objectives:

1) the coprimary objective of simple superiority of the ARCT-2303 vaccine booster (Groups 2a and 2b) compared to the ARCT-154 booster for the Omicron XBB.1.5 subvariant

Superiority of $GMT_{ARCT\text{-}2303\ (booster)}$ to $GMT_{ARCT\text{-}154\ (booster)}$

The superiority margin for the Omicron XBB.1.5 subvariant is defined as 1.0 for the GMTs ratio, thus the lower limit of the 2-sided 95% CI for GMT ratios (ARCT-2303/ARCT-154) is higher than 1.0.

Assumptions for the above calculations for objective 1) are:

The GMT ratio between ARCT-2303 (Day 29) and ARCT-154 (booster; ARCT-154-J01 study) (Day 29) groups is expected to be 1.5, and the common standard deviation is expected to be 0.60 in the $\log_{10}$ scale.

2) the coprimary noninferiority of the ARCT-2303 vaccine booster (Groups 2a and 2b) compared to the ARCT-154 booster for the Omicron XBB.1.5 subvariant

Noninferiority (NI) of $SCR_{ARCT\text{-}2303\ (booster)}$ minus $SCR_{ARCT\text{-}154}$

Assumptions for the above calculations for objective 2 are:

The difference between SCR in ARCT-2303 (Day 29) and SCR in ARCT-154 (booster; ARCT-154-J01 study) (Day 29) groups is expected to be >10% and the SCR is equal to 50% in the ARCT-154 group.

The noninferiority margin for the Omicron XBB.1.5 subvariant is defined as 5% for the SCRs difference, thus the lower limit of the 2-sided 95% CI for SCR difference (ARCT-2303 minus ARCT-154) is higher than -5% (minus 5%).

A sample size of up to 385 participants of study ARCT-154-J01, who received ARCT-154 vaccine booster, provided evaluable pre- and post-vaccination blood samples, and did not have SARS-CoV-2 infection and protocol deviations that impact on immunogenicity assessment

CONFIDENTIAL

May not be used, divulged, published or otherwise disclosed without written consent of Arcturus Therapeutics, Inc.

(PPS-1), and up to 560 participants in the ARCT-2303 (Groups 2a and 2b) provides approximately 99% power (n=350 and n=500 in the two groups if there is dropout of approximately 10%) to demonstrate each of the 2 coprimary objectives; therefore, there is at least 97% overall power to demonstrate the two coprimary objectives, 1 and 2.

If coprimary objectives 1 and 2 are met, then the second step will be assessing the noninferiority of immune response after co-administration and standalone administration of ARCT-2303 and Flucelvax Quadrivalent vaccines.

The noninferiority objectives for the co-administration are:

3) vaccination with Flucelvax Quadrivalent, when given concomitantly with ARCT-2303, compared to that of Flucelvax Quadrivalent, when given standalone

NI of GMT $_{\text{QIV+ ARCT-2303}}$ to GMT $_{\text{QIV + Placebo}}$ (Group 1a vs Group 3a)

4) vaccination with ARCT-2303, when given concomitantly with Flucelvax Quadrivalent, compared to that of ARCT-2303, when given standalone

NI of GMT $_{\text{ARCT-2303 + QIV}}$ to GMT $_{\text{ARCT-2303+placebo}}$ (Group 1a vs Group 2a)

The noninferiority margin is defined as 0.67 for the GMTs ratios.

Assumptions for the above calculations are:

the GMT ratios between the

a-Flucelvax Quadrivalent, when given concomitantly with ARCT-2303, compared to Flucelvax Quadrivalent, when given standalone,

b- ARCT-2303, when given concomitantly with Flucelvax Quadrivalent, compared to ARCT-2303, when given standalone,

is expected to be 1.0 and the common standard deviation is expected to be 0.60 in the $\log_{10}$ scale.

A sample size of 400 participants per group (Groups 1a, 2a, and 3a) provides 97.5% power (n=360 per group if there is dropout of approximately 10%) to demonstrate noninferiority for each one of the 5 strains (4 strains for influenza vaccine and 1 strain for ARCT-2303); therefore, there is at least 88% overall power to demonstrate all of the noninferiority objectives. The overall power to demonstrate all 4 coprimary objectives is at least 85%.

Populations for Analyses

The analysis sets are defined in Table 8.

May not be used, divulged, published or otherwise disclosed without written consent of Arcturus Therapeutics, Inc.

Table 8 Analysis Sets

Analysis Set	Description
Enrolled Set	All screened participants who provided informed consent, received a participant ID, and were randomized.
Exposed Set / Intent to Treat (ITT) Analysis Set	All participants who received at least one dose of a study vaccine.<br>The ITT Analysis Set will be analyzed according to the vaccine assigned.
Modified ITT Analysis Set	All participants who are randomized, received at least one dose of a study vaccine and provided at least one post-baseline immunogenicity assessment.<br>The mITT will be analyzed according to vaccine assigned.
Per Protocol (PP) Set	Participants in the mITT who correctly received all protocol-required doses of study vaccines at Day 1 and who at or prior to the specified timepoint have no evidence of SARS-CoV-2 infection and no protocol deviations impacting the analysis of immunogenicity data.<br>The PP set will be analyzed according to the vaccine assigned.
Safety Analysis Set (SAF)	All participants in the Exposed Set who provide any evaluable post-vaccination reactogenicity and/or safety data.<br>Should there be an error in administration where the actual study vaccine that the participant received was different than the one to which he/she was assigned, the Safety Analysis Set will be analyzed according to the vaccine actually received.
Historical Control Set (study ARCT-154-J01)	All participants from study ARCT-154-J01 who received a booster with ARCT-154 vaccine, provided evaluable pre- and post-vaccination blood samples, and did not have SARS-CoV-2 infection and protocol deviations that impact on immunogenicity assessment. These participants were included in the per protocol set 1 for the primary immunogenicity analysis

Abbreviations: AE, adverse event.

10.3 Statistical Analyses

The per protocol (PP) set and modified ITT analysis set (mITT) will be used for the primary objectives to assess superiority and noninferiority.

The GMT ratio for each of the primary immunogenicity endpoints will be assessed by using analysis of covariance (ANCOVA) model that will include covariates and stratification factors. Two-sided 95% CIs for GMT ratio will be obtained by taking the antilog of the confidence limits for the adjusted mean difference of the logarithmically transformed assay results which is calculated using t-distribution.

Descriptive immunogenicity analysis including GMT, GMFR, and SCR will be produced for each group at all available time points, along with the 95% CIs.

Geometric mean will be calculated as the mean of the antibody results after the data are log-transformed and then taking the antilog of the log-mean to present the results on the original scale. Two-sided 95% CI will be obtained by taking the log transformation of the antibody results and calculated based on t-distribution and then taking the antilog of the confidence limit. For the calculation of GMT and GMFR, imputation will be applied to antibody titer values above the upper limit of quantitation, ULOQ will be used. Antibody titer values below the lower limit of quantitation will be imputed as ½ LLOQ (i.e., 0.5*LLOQ).

CONFIDENTIAL

May not be used, divulged, published or otherwise disclosed without written consent of Arcturus Therapeutics, Inc.

GMFR analysis will include participants with antibody results available at both baseline (prior to dose) and post-vaccination. It will be calculated as the mean of the difference after log-transformed results (post baseline minus baseline for each participant) and exponentiating the mean. Two-sided 95% CI will be obtained by taking the log transform of the antibody results and calculating the 95% CI based on t-distribution for the mean difference after the data are log-transformed, then taking the antilog of the confidence limit. Percentage of participants with seroconversion will be summarized along with the 95%CI by the Clopper-Pearson method (Clopper et al., 1934).

Seroconversion is defined as binary variable for participants with non-missing values at pre-vaccination and post-vaccination as:

= 1, if there is a post-vaccination titer ≥4xLLOQ for pre-vaccination titer <LLOQ

= 1, if there is at least 4-fold increase (post-vaccination) from pre-vaccination titer ≥LLOQ

= 0, otherwise

The LLOQ will be defined before SAP finalization/database lock for the analysis.

An alternative SCR is also defined if there is at least a 4-fold increase (post vaccination) from pre-vaccination titer.

Seroconversion rate (SCR) will be calculated and 2-sided 95% CI by the Clopper-Pearson method will be produced.

The Safety Analysis set (SAF) will be used for all safety analyses which comprise all participants who received the study Day 1 vaccination/s. For safety and reactogenicity analysis, the descriptive summaries (mean, standard deviation [SD], median, minimum, maximum, etc., for continuous variables, counts, percentages and associated Clopper-Pearson 95% CIs for categorical variables) will be provided.

Individual data listings with information on the participant's study group will be generated as well at the final data analysis.

The final database lock will occur when all participants have completed the study.

All personnel involved in the analyses of the study will remain blinded until the final analysis and protocol deviations are identified.

The statistical analysis plan (SAP) will contain further details regarding the definition of analysis variables and analysis methodology to address all study objectives.

10.3.1 Analysis of Demographic and Baseline Characteristics

Descriptive statistics (mean, SD, median, minimum, and maximum) for age, height, weight, and BMI, risk factor for severe COVID-19 at enrollment will be calculated overall and by study group.

Distributions of participants by sex, age, and ethnic origin (race, ethnicity) will be summarized overall and by study group.

May not be used, divulged, published or otherwise disclosed without written consent of Arcturus Therapeutics, Inc.

10.3.2 Immunogenicity Analyses

Descriptive immunogenicity analysis including GMT, GMFR, and SCR will be produced for each group and subgroup at all available time points, along with the 95% CIs.

The methods for immunogenicity analysis are described in Table 9.

Table 9 Immunogenicity Analyses

Endpoint	Statistical Analysis Methods
Primary	Step 1<br>For SARS-CoV-2-neutralizing antibodies, the following analyses will be performed:<br><br>1. GMT ratio of $GMT_{ARCT\text{-}2303\ (Groups\ 2a\ and\ 2b)}$ (Day 29) over $GMT_{ARCT\text{-}154\ (booster)}$ (Day 29) and 95% CI by PP set for immunogenicity.<br>2. Difference of $SCR_{ARCT\text{-}2303\ (Groups\ 2a\ and\ 2b)}$ minus $SCR_{ARCT\text{-}154\ (booster)}$<br><br>Step 2<br>For HI assay titers against influenza vaccine strains and SARS-CoV-2-neutralizing antibodies, the following analyses will be performed:<br><br>3. GMT ratio of GMT $QIV_{QIV+ARCT\text{-}2303}$ over GMT $QIV_{QIV+Placebo}$ (Day 29) and 95% CI by PP set for immunogenicity.<br>4. GMT ratio of $ARCT\text{-}2303_{QIV+ARCT\text{-}2303}$ over GMT $ARCT\text{-}2303_{ARCT\text{-}2303+Placebo}$ and 95% CI by PP set for immunogenicity.<br><br>For Steps 1 and 2: The GMT ratios will be assessed by using ANCOVA model. Two-sided 95% CIs for GMT ratio will be obtained by taking the antilog of the confidence limits for the adjusted mean difference of the logarithmically transformed assay results which is calculated using t-distribution.<br>For Step 1: Seroconversion rate and its two-sided 95% CI for each group will be calculated using the Clopper-Pearson method. The difference of seroconversion rates between the two groups and its two-sided CI will be calculated using the Miettinen and Nurminen method. Stratification factors may be applied to calculate the SCR difference, which will be described in the SAP.
Secondary	For HI assay titers against influenza vaccine strains and for SARS-CoV-2-neutralizing antibodies, the following analyses will be performed:<br><br>5. Difference in SCRs of $QIV_{QIV+ARCT\text{-}2303}$ minus $QIV_{QIV+Placebo}$ (Day 29) and 95% CI by PP set for immunogenicity.<br>6. Difference in SCRs of $ARCT\text{-}2303_{QIV+ARCT\text{-}2303}$ minus $ARCT\text{-}2303_{ARCT\text{-}2303+Placebo}$ and 95% CI by PP set for immunogenicity.<br>Seroconversion rate and its two-sided 95% CI for each group will be calculated using the Clopper-Pearson method. The difference of seroconversion rates between the two groups and its two-sided CI will be calculated using the Miettinen and Nurminen method. Stratification factors may be applied to calculate the SCR difference, which will be described in the SAP.
Secondary	For VNA on Days 1, 29 and 181, the following analysis will be produced:<br>• GMT and 95% CI;<br>• GMFR and 95% CI;<br>• Number and proportion of participants with seroconversion (SCR) and 95% CI;

	• Proportion of participants with antibody titer above LLOQ and 95% CI<br>For HI assay titers against influenza vaccine strains on Days 1 and 29, the following analysis will be produced:<br>• GMT and 95% CI;<br>• GMFR and 95% CI;<br>• Number and proportion of participants with seroconversion (SCR) and 95% CI;<br>• Proportion of participants with HI titer ≥1:40 and 95% CI<br>Geometric mean will be calculated as the mean of the antibody results after the data are log-transformed and then taking the antilog of the log-mean to present the results on the original scale. Two-sided 95% CI will be obtained by taking the log transform of the antibody results and calculating based on t-distribution; then taking the antilog of the confidence limit.<br>GMFR analysis will include participants with antibody results available at both baseline (prior to Dose 1) and post-vaccination. It will be calculated as the mean of the difference after log-transformed results (post baseline minus baseline) and taking the antilog of the mean. Two-sided 95% CI will be obtained by taking the log transform of the antibody results and calculating the 95% CI based on Student's t-distribution for the mean difference after the data are log-transformed, then taking the antilog of the confidence limit.<br>The SCR will be calculated along with the 2-sided 95% CI by Clopper-Pearson method.<br>The percentage of participants with antibody titer above a prespecified threshold will be calculated along with the 2-sided 95% CI by Clopper-Pearson method.<br>The analysis will be performed on PP and mITT if more than 5% of participants are excluded from PP set. Missing data will not be imputed.

May not be used, divulged, published or otherwise disclosed without written consent of Arcturus Therapeutics, Inc.

10.3.3 Safety Analyses

The methods for the safety statistical analyses are described in Table 10.

Table 10 Safety Analyses

Endpoint	Statistical Analysis Methods
Secondary	**Reactogenicity (solicited local and systemic AEs):**<br>• Percentages of participants experiencing each solicited AE will be summarized for each symptom by any (severity), maximum severity (mild, moderate or severe, except fever) during 7 days after vaccination by study arm. For the definition of severity grades, refer to Table 7.<br>• Duration and time of onset of events will be summarized by study arm descriptively by mean, SD etc.<br>• Injection site erythema and swelling will be summarized according to categories based on linear measurements.<br>• Use of antipyretics and analgesics will be summarized by frequency and percentage of participants reporting use.<br>• Body temperature will be summarized by 0.5°C increments from 38.0°C up to ≥40°C. In addition, fever will be summarized according to “mild”, “moderate” or “severe” categorization.<br>**Unsolicited AEs, SAEs, MAAEs, and AESIs:**<br>• Percentages of participants reporting at least one AE, related AE, SAE, MAAE, AESI within 28 days after each study vaccination will be summarized by study arm.<br>• Percentages of participants reporting at least one SAE, MAAE, AESI.<br>• Percentages of participant withdrawal/early termination from the study will be summarized by study arm.<br>• Percentages of participants reporting any AEs within 30 minutes after each study vaccination will be summarized by study group.<br>AEs will be coded using the most recent version of MedDRA. AEs will be presented by SOC and PT by study group.<br>Data listings of all AEs will be provided by participant at final analysis.<br>All safety analyses will be performed on the SAF. Missing data will not be imputed.

Abbreviations: AE, adverse event; AESI, adverse event of special interest; MAAE, medically attended adverse event; MedDRA, Medical Dictionary for Regulatory Activities; PT, preferred term; SAE, serious adverse event; SAF, safety analysis set; SD, standard deviation; SOC, system organ class.

10.4 Interim Analyses and Analysis Timing

No interim analysis is planned for the study.

The results of this study will be released sequentially in two separate final analyses.

10.4.1 Final Analyses – First Analysis

The first final analysis will include all immunogenicity data (up to Day 29), reactogenicity data (7 days post-vaccination), unsolicited AEs (28 days post-vaccination), and safety data collected from Day 1 to Day 57 and associated primary and secondary objectives. This analysis will be conducted on cleaned and frozen data. The results of this analysis will be presented in the initial clinical study report (CSR). Immunogenicity and safety data will be reported on a group level only. No individual listings will be generated. Access to participant-level information about study groups will be restricted to specified personnel involved in the statistical analysis. Site staff, CROs, sponsor representatives remain blinded at the participant level.

10.4.2 Final Analyses – Second Analysis

The second final analysis of immunogenicity and safety data collected from Day 57 to the study end will be performed as soon as all data are available. The results of this analysis will be presented in the final CSR. All individual data listings with information on participant study group will be presented in the addendum to the CSR.

10.5 Data Safety Monitoring Board and Central Cardiac Adjudication Committee

The study will include the use of an independent unblinded Data Safety Monitoring Board (DSMB) and a Central Cardiac Adjudication Committee.

The DSMB will meet periodically to review study data and provide recommendations to the Sponsor during the entire study period. The members of the DSMB serve in an individual capacity and provide their expertise, including recommendations regarding the continuation, modification, or termination of the study. The DSMB may review cumulative study data to evaluate safety, study conduct, scientific validity, and data integrity of the study. Further details are provided in the DSMB charter.

A central cardiac adjudication committee may meet to assess any potential myocarditis/pericarditis cases (see Section 9.1.14.1). Further details are provided in the cardiac adjudication committee charter.

CONFIDENTIAL

May not be used, divulged, published or otherwise disclosed without written consent of Arcturus Therapeutics, Inc.

11 REFERENCES

1. WHO Coronavirus (COVID-19) Dashboard. World Health Organization. https://covid19.who.int/ (Accessed 05 May 2023).
2. Domnich A, Orsi A, Trombetta CS, Guarona G, Panatto D, Icardi G. COVID-19 and Seasonal Influenza Vaccination: Cross-Protection, Co-Administration, Combination Vaccines, and Hesitancy. Pharmaceuticals (Basel). 2022 Mar 8;15(3):322. doi: 10.3390/ph15030322. PMID: 35337120; PMCID: PMC8952219.
3. Lazarus R, Baos S, Cappel-Porter H. et al. Safety and immunogenicity of concomitant administration of COVID-19 vaccines (ChAdOx1 or BNT162b2) with seasonal influenza vaccines in adults in the UK (ComFluCOV): a multicentre, randomised, controlled, phase 4 trial. Lancet. 2021;398(10318):2277-2287. doi: 10.1016/S0140-6736(21)02329-1.
4. Sexson Tejtel SK, Munoz FM, Al-Ammouri I, Savorgnan F, Guggilla RK, Khuri-Bulos N, Phillips L, Engler RJM. Myocarditis and pericarditis: Case definition and guidelines for data collection, analysis, and presentation of immunization safety data. Vaccine. 2022 Mar 1;40(10):1499-1511. doi: 10.1016/j.vaccine.2021.11.074. Epub 2022 Jan 31. PMID: 35105494.
5. COVID-19 Vaccine Tracker. European Centre for Disease Prevention and Control. https://vaccinetracker.ecdc.europa.eu/public/extensions/COVID-19/vaccine-tracker.html#uptake-tab (Accessed 05 May 2023).
6. COVID-19 Vaccination Coverage and Vaccine Confidence Among Adults. US Centers for Disease Control and Prevention. https://www.cdc.gov/vaccines/imz-managers/coverage/covidvaxview/index.html (Accessed 05 May 2023).
7. Coronavirus (COVID-19) Vaccinations. Our World in Data https://ourworldindata.org/covid-vaccinations (Accessed 05 May 2023).
8. Underlying Medical Conditions Associated with Higher Risk for Severe COVID-19: Information for Healthcare Professionals. US CDC. 09 February 2023. https://www.cdc.gov/coronavirus/2019-ncov/hcp/clinical-care/underlyingconditions.html (Accessed 05 May 2023).
9. Rüggeberg JU, Gold MS, Bayas JM, Blum MD, Bonhoeffer J, Friedlander S, et al. Anaphylaxis: Case definition and guidelines for data collection, analysis, and presentation of immunization safety data. Vaccine. 2007;25(31):5675-84.
10. Food and Drug Administration Guidance Document: Toxicity Grading Scale for Healthy Adult and Adolescent Volunteers Enrolled in Preventive Vaccine Clinical Trials. Available at https://www.fda.gov/media/73679/download (Accessed 05 May 2023).
11. Clopper C, Pearson ES. The use of confidence or fiducial limits illustrated in the case of the binomial. Biometrika. 1934;26 (4): 404–413.
12. Safety Platform for Emergency vACcines (SPEAC). Updated (October 2022) COVID-19 AESI including status of associated Brighton case definitions Available at: https://brightoncollaboration.us/wp-content/uploads/2022/11/Updated-COVID-19-AESI-list_Oct2022-1.pdf (Accessed 05 May 2023).
13. US FDA Guidance for Industry. Emergency Use Authorization for Vaccines to Prevent COVID-19. Document issued on 31 March 2022. https://www.fda.gov/media/142749/download (Accessed 05 July 2023).

14. Updated COVID-19 Vaccines for Use in the United States Beginning in Fall 2023. US CDC. Document issued on 16 June 2023. https://www.fda.gov/vaccines-blood-biologics/updated-covid-19-vaccines-use-united-states-beginning-fall-2023 (Accessed 05 July 2023).
15. ECDC-EMA statement on updating COVID-19 vaccines composition for new SARS-CoV-2 virus variants. Document issued on 07 June 2023. https://www.ecdc.europa.eu/sites/default/files/documents/covid-19-vaccines-composition-variants-statement-ECDC-EMA_0.pdf (Accessed 12 July 2023).
16. Statement on the antigen composition of COVID-19 vaccines. WHO. Document issued on 18 May 2023. https://www.who.int/news/item/18-05-2023-statement-on-the-antigen-composition-of-covid-19-vaccines (Accessed 12 July 2023).
17. CTFG Recommendations related to contraception and pregnancy testing in clinical trials. Document issued on 15 September 2014. Microsoft Word - Contraception20140912 (hma.eu) (Accessed 12 January 2024).

CONFIDENTIAL

May not be used, divulged, published or otherwise disclosed without written consent of Arcturus Therapeutics, Inc.

12 APPENDICES

Appendix 1 Contraceptive Guidance and Collection of Pregnancy Information[17]

Definitions:

Woman of Childbearing Potential (WOCBP)

A woman is considered fertile following menarche and until becoming postmenopausal unless permanently sterile (see below).

Women in the following categories are not considered as women of childbearing potential

1. Premenarchal
2. Premenopausal female with 1 of the following:
   a) Documented hysterectomy.
   b) Documented bilateral salpingectomy.
   c) Documented bilateral oophorectomy.

Note: Documentation can come from the study center personnel's review of the participant's medical records, medical examination, or medical history interview.

3. Postmenopausal female:
   a) A postmenopausal state is defined as no menses for 12 months without an alternative medical cause. A high follicle-stimulating hormone (FSH) level in the postmenopausal range may be used to confirm a postmenopausal state in women not using hormonal contraception or hormonal replacement therapy (HRT). However, in the absence of 12 months of amenorrhea, a single FSH measurement is insufficient.
   b) Females on HRT and whose menopausal status is in doubt will be required to use 1 of the non-estrogen hormonal highly effective contraception methods if they wish to continue their HRT during the study. Otherwise, they must discontinue HRT to allow confirmation of postmenopausal status before study enrollment.

Female Participant Reproductive Inclusion Criteria

A female participant is eligible to participate if she is not pregnant or breastfeeding and at least 1 of the following conditions applies:

- Is not a WOCBP (see definitions above).

OR

- Is a WOCBP and uses an acceptable contraceptive method as described below during the intervention period (for a minimum of 60 days after the last dose of study intervention; or as required by local regulations). The Investigator should evaluate the effectiveness of the contraceptive method in relationship to the first dose of study intervention.

The Investigator is responsible for review of medical history, menstrual history, and recent sexual activity to decrease the risk for inclusion of a woman with an early undetected pregnancy.

CONFIDENTIAL

May not be used, divulged, published or otherwise disclosed without written consent of Arcturus Therapeutics, Inc.

Contraception Guidance

Female participants of childbearing potential are eligible to participate if they agree to use a highly effective method of contraception consistently and correctly as described in Table 11.

Table 11 Highly Effective Contraceptive Methods

**Highly Effective Contraceptive Methods That Are User-Dependent**[a]<br>*Failure rate of <1% per year when used consistently and correctly.*
Combined (estrogen and progestogen containing) hormonal contraception associated with inhibition of ovulation[b].<br>• Oral<br>• Intravaginal<br>• Transdermal
Progestogen only hormonal contraception associated with inhibition of ovulation.<br>• Oral<br>• Injectable[c]
**Highly Effective Methods That Are User-Independent** [a]
Implantable progestogen only hormonal contraception associated with inhibition of ovulation[b]<br>• Intrauterine device (IUD)<br>• Intrauterine hormone-releasing system (IUS)<br>• Bilateral tubal occlusion
**Vasectomized partner**<br>*A vasectomized partner is a highly effective birth control method provided that the partner is the sole male sexual partner of the woman of child-bearing potential and the absence of sperm has been confirmed. If not, an additional highly effective method of contraception should be used.*
**Sexual abstinence**<br>*Sexual abstinence is considered a highly effective method only if defined as refraining from heterosexual intercourse during the entire period of risk associated with the study vaccine. The reliability of sexual abstinence needs to be evaluated in relation to the duration of the study and the preferred and usual lifestyle of the participant.*

NOTES:
[a] Typical use failure rates may differ from those when used consistently and correctly. Use should be consistent with local regulations regarding the use of contraceptive methods for participants participating in clinical studies.
[b] Hormonal contraception may be susceptible to interaction with the study vaccine, which may reduce the efficacy of the contraceptive method. As a general rule, use of hormonal contraception is not recommended if a clinically relevant interaction with contraceptive steroids has been observed or is suspected. If an interaction with contraceptive steroids has been observed or is suspected, but the effect is considered to be of limited clinical significance, the hormonal contraception method must be supplemented with a barrier method (preferably male condom).[c] Combination contraceptive injectables are allowed if licensed in the respective countries and have a failure rate <1% per year.

CONFIDENTIAL

May not be used, divulged, published or otherwise disclosed without written consent of Arcturus Therapeutics, Inc.

Pregnancy Testing

- Women of childbearing potential should only be included after a confirmed menstrual period and a negative highly sensitive urine pregnancy test or serum pregnancy test, as locally required.
- Additional pregnancy testing should be performed prior to the second vaccination and as required locally.

Pregnancy testing will be performed whenever a menstrual cycle is missed or when pregnancy is otherwise suspected.

Collection of Pregnancy Information

Pregnancy does not meet the definition of an AE. However, to fulfill regulatory requirements, any pregnancy should be reported to collect data on the outcome for both mother and fetus.

Female participants who become pregnant

- The investigator will collect pregnancy information on any female participant who becomes pregnant while participating in this study. Information will be recorded on the Pregnancy Report Form and submitted to the Sponsor within 24 hours of learning of a participant's pregnancy. The participant will be followed to determine the outcome of the pregnancy. The investigator will collect follow-up information on the participant and the neonate, and the information will be forwarded to the Sponsor.
- Any female participant who becomes pregnant while participating in the study will discontinue the study vaccine. The participant will be encouraged to remain in the study for safety follow-up until the end of the study.

Reporting of Pregnancy Information

Information will be recorded by the investigator on the appropriate paper Pregnancy Report Form and submitted to the Sponsor/Sponsor's designee within 24 hours of learning of a pregnancy case and upon learning of any follow-up pregnancy information. The contacts (email) for pregnancy reporting can be found in the instructions page of the paper Pregnancy Report Form.

A paper Pregnancy Report Form will be completed for any pregnancy occurring in participants after the first vaccination during the entire study period.

While pregnancy itself is not considered to be an AE, any pregnancy complications (e.g., congenital abnormalities/birth defects and spontaneous miscarriages) should also be reported as SAEs.

The outcome of all pregnancies (spontaneous miscarriage, elective termination, ectopic pregnancy, normal birth, or congenital abnormality) should be followed up and reported regardless of fetal status (presence or absence of anomalies) or indication for the procedure.

Any post-study pregnancy related SAE considered reasonably related to the study vaccine by the investigator will be reported to the Sponsor. While the investigator is not obligated to actively seek this information in former participants, he or she may learn of an SAE through spontaneous reporting.

CONFIDENTIAL

May not be used, divulged, published or otherwise disclosed without written consent of Arcturus Therapeutics, Inc.

Appendix 2 Regulatory, Ethical, and Study Oversight Considerations

Regulatory and Ethical Considerations

- This study will be conducted in accordance with the protocol and with the following:
  - Consensus ethical principles derived from international guidelines including the Declaration of Helsinki and Council for International Organizations of Medical Sciences (CIOMS) International Ethical Guidelines.
  - Applicable ICH GCP Guidelines.
  - Applicable laws and regulations.
- The protocol, protocol amendments, ICF, IB, and other relevant documents (e.g., advertisements) must be submitted to an IRB/IEC by the investigator and reviewed and approved by the IRB/IEC before the study is initiated.
- Any amendments to the protocol will require IRB/IEC and regulatory authority approval, when applicable, before implementation of changes made to the study design, except for changes necessary to eliminate an immediate hazard to participants.
- The investigator will be responsible for the following:
  - Providing written summaries of the status of the study to the IRB/IEC annually or more frequently in accordance with the requirements, policies, and procedures established by the IRB/IEC.
  - Notifying the IRB/IEC of SAEs or other significant safety findings as required by IRB/IEC procedures.
  - Providing oversight of the conduct of the study at the study center and adherence to requirements of 21 CFR, ICH guidelines, the IRB/IEC, European regulation 536/2014 for clinical studies (if applicable), and all other applicable local regulations.

After reading the protocol, each investigator will sign the protocol signature page and send a copy of the signed page to the Sponsor or representative (Appendix 5). The study will not start at any study center at which the investigator has not signed the protocol.

Financial Disclosure

Investigators and sub-investigators will provide the Sponsor with sufficient, accurate financial information as requested to allow the Sponsor to submit complete and accurate financial certification or disclosure statements to the appropriate regulatory authorities. Investigators are responsible for providing information on financial interests during the course of the study and for 1 year after completion of the study.

Insurance

The Sponsor will provide insurance in accordance with local guidelines and requirements as a minimum for the participants in this study. The terms of the insurance will be kept in the study files.

CONFIDENTIAL

May not be used, divulged, published or otherwise disclosed without written consent of Arcturus Therapeutics, Inc.

Informed Consent Process

- The investigator or his/her representative will explain the nature of the study to the participant and answer all questions regarding the study.
- Participants must be informed that their participation is voluntary. Participants will be required to sign a statement of informed consent that meets the requirements of 21 CFR 50, local regulations, ICH guidelines, Health Insurance Portability and Accountability Act requirements, where applicable, and the IRB/IEC or study center.
- The medical records must include a statement that written informed consent was obtained before the participant was entered in the study and the date the written consent was obtained. The authorized person obtaining the informed consent must also sign the ICF.
- Participants must be re-consented to the most current version of the ICF(s) during their participation in the study.
- A copy of the ICF(s) must be provided to the participant.
- The investigator (or designee) must provide a “participant card” to each participant. In an emergency situation, this card serves to inform the responsible attending physician that the participant is in a clinical study and that relevant information may be obtained by contacting the investigator. The address and telephone number of the main contact for information about the clinical study must appear on the card. Participants must be instructed to keep participant cards in their possession at all times during the study duration.

A participant who is rescreened is not required to sign another ICF if the rescreening occurs within 14 days from the previous ICF signature date.

Data Protection

- Participants will be assigned a unique identifier by the Sponsor. Any participant records or datasets that are transferred to the Sponsor will contain the identifier only; participant names or any information which would make the participant identifiable will not be transferred.
- The participant must be informed that his/her personal study-related data will be used by the Sponsor in accordance with local data protection law. The level of disclosure must also be explained to the participant.
- The participant must be informed that his/her medical records may be examined by clinical quality assurance auditors or other authorized personnel appointed by the Sponsor, by appropriate IRB/IEC members, and by inspectors from regulatory authorities.

May not be used, divulged, published or otherwise disclosed without written consent of Arcturus Therapeutics, Inc.

Administrative Structure

The administrative structure for this study is presented in Table 12.

Table 12 Study Administrative Structure

Function	Responsible Organization
Study Operations Management	CRO
Medical Monitoring	CRO/study center
Study Master File	CRO
Randomization Code	CRO
Data Management	CRO
Clinical Supply Management	Third party
Quality Assurance Auditing	Sponsor
Biostatistics	CRO
Medical Writing	Third party
Laboratory Assessments	Third parties
DSMB (see Section 10.5)	Sponsor/CRO

Abbreviations: CRO, contract research organization; DSMB: data safety monitoring board

Dissemination of Clinical Study Data

The results of the study should be reported within 1 year from the end of the clinical study. Irrespective of the outcome, the Sponsor will submit to the regulatory authority database a summary of the results of the clinical study within 1 year from the end of the clinical study. It shall be accompanied by a summary written in a manner that is understandable to laypersons.

Data Quality Assurance

- All participant data relating to the study will be recorded on eCRFs unless transmitted to the Sponsor or designee electronically (e.g., laboratory data). The investigator is responsible for verifying that data entries are accurate and correct by electronically signing the eCRF.
- The investigator must maintain accurate documentation (source data) that supports the information entered in the eCRF.
- The investigator must permit study-related monitoring, audits, IRB/IEC review, and regulatory agency inspections and provide direct access to source data documents.
- The Sponsor or designee is responsible for the data management of this study including quality checking of the data.
- Study monitors will perform ongoing source data verification to confirm that data entered into the eCRF by authorized study center personnel are accurate, complete, and verifiable from source documents; that the safety and rights of participants are being protected; and that the study is being conducted in accordance with the currently approved protocol and any other study agreements, ICH GCP, and all applicable regulatory requirements.

CONFIDENTIAL

May not be used, divulged, published or otherwise disclosed without written consent of Arcturus Therapeutics, Inc.

- Records and documents, including signed ICFs, pertaining to the conduct of this study must be retained by the investigator for 15 years after study completion unless local regulations or institutional policies require a longer retention period. No records may be destroyed during the retention period without the written approval of the Sponsor. No records may be transferred to another location or party without written notification to the Sponsor.

Source Documents

The investigator/institution should maintain adequate and accurate source documents and study records that include all pertinent observations on each of the study center's participants. Source data should be prior, legible, contemporaneous, original, accurate, and complete. Changes to source data should be traceable, should not obscure the original entry, and should be explained if necessary (e.g., via an audit trail).

- Source documents provide evidence for the existence of the participant and substantiate the integrity of the data collected. Source documents are filed at the investigator's study center.
- Data entered in the eCRF that are transcribed from source documents must be consistent with the source documents or the discrepancies must be explained. The investigator may need to request previous medical records or transfer records, depending on the study. Also, current medical records must be available.

Study and Study Center Closure

The Sponsor or designee reserves the right to close the study center or terminate the study at any time for any reason at the sole discretion of the Sponsor. Study centers will be closed upon study completion. A study center is considered closed when all required documents and study supplies have been collected and a study center closure visit has been performed. The investigator may initiate study center closure at any time, provided there is reasonable cause and sufficient notice is given in advance of the intended termination. Reasons for the early closure of a study center by the Sponsor or investigator may include but are not limited to:

- Failure of the investigator to comply with the protocol, the requirements of the IRB/IEC or local health authorities, the Sponsor's procedures, or GCP guidelines.
- Inadequate recruitment of participants by the investigator.
- Discontinuation of further study vaccine development.

Publication Policy

The data generated by this study are confidential information of the Sponsor. The Sponsor will make the results of the study publicly available. The publication policy with respect to the investigator and study center will be set forth in the Clinical Trial Agreement.

- The results of this study may be published or presented at scientific meetings. If this is foreseen, the investigator agrees to submit all manuscripts or abstracts to the Sponsor before submission. This allows the Sponsor to protect proprietary information and to provide comments.

May not be used, divulged, published or otherwise disclosed without written consent of Arcturus Therapeutics, Inc.

- The Sponsor will comply with the requirements for publication of study results. In accordance with standard editorial and ethical practice, the Sponsor will generally support publication of multicenter studies only in their entirety and not as individual study center data. In this case, a coordinating investigator will be designated by mutual agreement.
- Authorship will be determined by mutual agreement and in line with International Committee of Medical Journal Editors authorship requirements.

CONFIDENTIAL

May not be used, divulged, published or otherwise disclosed without written consent of Arcturus Therapeutics, Inc.

Appendix 3 Adverse Events of Special Interest

Table 13 List of Adverse Events of Special Interest Relevant to COVID-19 (Guidance Document from Safety Platform for Emergency vACcines [SPEAC])

Acute respiratory distress syndrome
Multisystem inflammatory syndrome (children and adults)
Myocarditis / pericarditis
Other forms of acute cardiac injury including arrhythmias, heart failure, coronary artery disease, myocardial infarction, stress cardiomyopathy
Thrombosis and Thromboembolism
Hemorrhagic disorders including DIC
Anosmia, ageusia
Chilblain like lesions
Erythema multiforme
Single organ cutaneous vasculitis
Acute kidney injury
Acute Liver injury
Acute pancreatitis
Rhabdomyolysis
Subacute thyroiditis
Anaphylaxis [1, 2]
Thrombocytopenia [1, 2, 3, 4]
Generalized convulsion [1, 2]
Acute disseminated encephalomyelitis [4]
Guillain Barre Syndrome [3, 4]
Idiopathic Peripheral Facial Nerve Palsy [Intranasal EColi Heat Labile Toxin Adjuvanted Vaccine]
Vaccine associated enhanced disease [1(Formalin inactivated measles/RSV; HIV), 2(Chimeric YF Dengue), 5 (SARS / MERS-CoVs)]
Thrombocytopenia and Thrombosis Syndrome[6]
Immune Thrombocytyopenia (ITP)[6]
Capillary Leak Syndrome[6] (Flare up in individuals with prior history of capillary leak syndrome)
Delayed hypersensitivity reaction[6]
Extensive limb swelling[6]
Facial swelling in individuals with dermal fillers[6]
Dizziness and tinnitus[6]

Abbreviations: AESI, adverse event of special interest; DIC, Disseminated intravascular coagulation.
[1] Proven association with immunization encompassing several different vaccines
[2] Proven association with vaccine that could theoretically be true for novel COVID-19 vaccines
[3] Theoretical concern based on wild type disease immunopathogenesis
[4] Theoretical concern related to viral replication during wild type disease
[5] Theoretical concern because it has been demonstrated in an animal model with ≥ 1 vaccine platform
[6] Signal recognized and validated during COVID-19 mass campaigns or regulator(s) required update to product information
* Acute kidney injury–consensus definition of Kidney Disease Improving Global Outcomes expert consensus group www.kdigo.org

- Increase in serum creatinine by ≥ 0.3 mg/dl (≥26 µmol/l) within 48 hours; OR
- Increase in serum creatinine to ≥ 1.5 times baseline, known or presumed to have occurred within prior 7 days OR
- Urine volume ≤0.5 ml/ kg/ hour for 6 hours

Acute liver injury – definition as used in majority of COVID-19 publications (but no international consensus):

- 3-fold elevation above the upper normal limit for Alanine transaminase or Aspartate aminotransferase OR

- 2-fold elevation above the upper normal limit for total serum bilirubin or Gamma-glutamyl Transferase or Alkaline phosphatase.

Case Definition and resources available at:
https://docs.google.com/spreadsheets/d/1QgF35nYcsaFN3DZTOtV_lP0TYqQzsDMUQBAd5M9brrM/edit#gid=1666959512 Accessed 29 April 2023.

May not be used, divulged, published or otherwise disclosed without written consent of Arcturus Therapeutics, Inc.

Appendix 4 Protocol Amendments

Protocol Amendment 1 (Version 1.1, 11 September 2023): Summary of Changes

Section Number and Name	Description of Change	Brief Rationale
Throughout Protocol	Corrected spelling, grammar, and formatting.	Improve clarity.
Title Page, Protocol Approval – Sponsor, Declaration of Investigator, Page Header	Revised version and date of the protocol from 24 July 2023, Version 1.0 to 12 September 2023, Version 1.1.	Updated version and date of protocol due to protocol amendment.
Synopsis – Study Design	Specified location of Figure 1 and Table 1.	Improve clarity.
Synopsis – Study Objectives, Protocol – Table 4	Numbering of primary objectives added, number of hypotheses for influenza strain testing (primary objective 3) added.	Improve clarity; align data between synopsis and protocol.
Synopsis – Study Objectives – Success Criteria, Protocol – Overall Design – Success Criteria	Success criteria and hierarchy of testing added.	Include additional details per U.S. FDA request
Synopsis – Sample Size	Overall power to demonstrate all 4 coprimary objectives added.	Improve clarity.
Synopsis – Sequence of the Analysis	Description of the sequence of analyses added. Step 3 of the analysis and associated hypotheses H_{05} (secondary objective 2) and H_{06} (secondary objective 1) now included.	Include additional details per U.S. FDA request.
Protocol – Table 1	Window for baseline ECG recording added.	Provide flexibility for clinical sites.
Protocol – Exclusion Criteria	Exclusion criteria #1 revised to specify requirements for inclusion of individuals with suspected COVID-19.	Improve clarity.
Protocol – Phone Contact Day 91	Requirements for safety information collection on Day 91 added.	Improve clarity.
Protocol – Assessment of Intensity of AEs	Additional information provided for investigator's convenience.	Improve clarity.
Protocol – Statistical Considerations	Description of hypotheses testing steps included.	Provide additional information per U.S. FDA request.
Protocol – Statistical Hypotheses and Hypothesis Testing	Step 3 Hypotheses testing approach added.	Improve clarity.
Protocol – Table 8	Definition of "Enrolled Population" corrected.	Improve clarity.
Protocol - Statistical Analyses	Approach for imputation of antibody titers above HLOQ and below LLOQ added.	Address U.S. FDA request.

CONFIDENTIAL

May not be used, divulged, published or otherwise disclosed without written consent of Arcturus Therapeutics, Inc.

Section Number and Name	Description of Change	Brief Rationale
Protocol – Table 9	Use of stratification factors in Miettinen-Nurminen method for SCR difference specified.	Address U.S. FDA request.
Protocol -References	Corrected grammar and formatting.	Improve clarity.

Protocol Amendment 2 (Version 2.0, 21 February 2024): Summary of Changes

Section Number and Name	Description of Change	Brief Rationale
Throughout Protocol	Corrected spelling, grammar, and formatting.	Improve clarity.
Synopsis – Main Inclusion and Exclusion Criteria	Main inclusion and exclusion criteria updated to include criteria related to pregnancy, breastfeeding, and adherence to required contraception methods for individuals of childbearing potential.	To address EC recommendations and align with the protocol.
Synopsis – Secondary Immunogenicity Objectives<br>Protocol - Table 4 Objectives, Endpoints, Estimands	SCR will not be performed at Day 181 (objectives #3 and #5).	Clarification.
Synopsis – Secondary Immunogenicity Objectives<br>Protocol - Overall Design	Randomization will not be stratified by site.	Clarification.
Synopsis, Protocol - Background	Specified that the Southern Hemisphere 2024 season vaccine composition for both influenza vaccines will be used in the study.	Clarification to address RA recommendation.
Protocol – Randomization	Stratification included.	Alignment throughout the protocol/synopsis.
Protocol - Table 1 Schedule of Assessments	Timepoints for collection of blood samples for immunogenicity assessment and cardiac enzymes and for collection of AEs clarified in footnotes.	Clarification and alignment throughout the protocol/synopsis.
Synopsis, Protocol – Exclusion Criteria	Exclusion criteria #1 revised to specify temperature is to be measured by any method and fever is defined as temperature of ≥ 38°C (≥101.4°F).	Alignment throughout the protocol/synopsis. Provide flexibility to sites.
Synopsis, Protocol – Exclusion Criteria	Exclusion criteria # 5 updated to include history of severe hypersensitivity to any vaccine.	Clarification and alignment.
Protocol – Exclusion Criteria; Prohibited Medications	Receipt of any licensed vaccines within 14 days prior to Day 1 or up	Clarification and alignment.

Section Number and Name	Description of Change	Brief Rationale
	to 14 days after the second study vaccination.	
Protocol – Study Assessments and Procedures; Pre-vaccination Procedures	Included collection of cardiac enzymes at baseline.	Alignment throughout the protocol/synopsis.
Synopsis, Protocol – Physical examination and Vital Signs	Specifications on vital signs collection.	Clarification.
Protocol – Serological Testing	A random subset of samples from Groups 2a and 2b will be tested using VNA to assess antibody persistence at Day 181.	Alignment with Table 6.
Protocol – eDiary Instructions	No paper diary used in the study.	Clarification.
Protocol – Assessment of Outcome of AE	All fatal events are considered SAEs.	Clarification.
Protocol – Table 7	Grade 3 fever defined as temperature ≥39°C	Correct typographical error.
Protocol – Follow-up of AESIs and SAEs	Specify follow-up of SAEs/AESIs ongoing at end of study.	Clarification.
Protocol – Pregnancy	Details of pregnancies in female partners of male participants will be collected according to local regulations.	Clarification.
Protocol – Adverse Events of Special Interest	No PT list of AESIs and questionnaires to be provided to investigators.	The list of AESIs is present in Table 13; targeted questionnaires are used by Sponsor/CRO to review reported event(s).
Protocol – Table 9	Remove Miettinen-Nurminen method from statement regarding application of stratification factors to analyze the SCR difference.	Methods similar to Miettinen-Nurminen may be used.
Appendix 1	Female Participant Reproductive Inclusion Criteria included.<br>Contraception guidance aligned with recommendations.<br>Pregnancy should be reported to collect data on the outcome for both mother and fetus.	Clarification and update as per CTFG recommendations.<br>Aligned with protocol.
Synopsis, Protocol, Appendix 1	Serum pregnancy test to be done as locally required.	Clarification.
References	CTFG Recommendations included.	Updated.

CONFIDENTIAL

May not be used, divulged, published or otherwise disclosed without written consent of Arcturus Therapeutics, Inc.

Appendix 5 Investigator Signature

PROTOCOL TITLE: A Phase 3, Multicenter, Observer-blind, Randomized, Controlled Study to Evaluate the Immunogenicity, Reactogenicity, and Safety of a Self-Amplifying RNA COVID-19 Vaccine (ARCT-2303), Administered Concomitantly with Quadrivalent Influenza Vaccines, in Adults

PROTOCOL NO: ARCT-2303-01

VERSION: Protocol Version 2.0 (21 February 2024)

This protocol is a confidential communication of the Sponsor. I confirm that I have read this protocol, I understand it, and I will work according to this protocol. I will also work consistently with the ethical principles that have their origin in the Declaration of Helsinki and that are consistent with Good Clinical Practices and the applicable laws and regulations. Acceptance of this document constitutes my agreement that no unpublished information contained herein will be published or disclosed without prior written approval from the Sponsor.

Instructions to the Investigator: Please SIGN and DATE this signature page. PRINT your name, title, and the name of the study center in which the study will be conducted. Return the signed copy to the CRO.

I have read this protocol in its entirety and agree to conduct the study accordingly:

Signature of the Investigator: ______________________________ Date: ___________

Printed Name: ______________________________

Investigator Title: ______________________________

Name/Address of Study Center: ____
