# Supplementary material for: Immunogenicity and safety of self-amplifying mRNA COVID-19 vaccine (ARCT-2303), with or without co-administration of seasonal inactivated influenza vaccine in adults: a phase 3, randomised, controlled, observer-blind, multicentre study
Source: eClinicalMedicine. 2025 Aug 20;87:103428. doi: 10.1016/j.eclinm.2025.103428 (PMC12396474; doi:10.1016/j.eclinm.2025.103428)
Supplement: Statistical Analysis Plan [file mmc2.pdf]

NCT #: NCT06279871

NOVOTECH HOLDINGS

STATISTICAL ANALYSIS PLAN

A Phase 3, Multicenter, Observer-blind, Randomized, Controlled Study to Evaluate the Immunogenicity, Reactogenicity, and Safety of a Self-Amplifying RNA COVID-19 Vaccine (ARCT-2303), Administered Concomitantly with Quadrivalent Influenza Vaccines, in Adults

PROTOCOL NO.: **ARCT-2303-01**

PRODUCT CODE: ARCT-2303

**PREPARED FOR:**	Arcturus Therapeutics, Inc.<br>10628 Science Center Dr #250<br>San Diego, CA, 92121<br>USA
**DATE OF ISSUE:**	**2024-12-18**
**VERSION/STATUS:**	Version 3.0 (Amendment 2.0)
**VERSION HISTORY:**	Version 1.0, 29-May-2024<br>Version 2.0 (Amendment 1.0), 23-Aug-2024
**AUTHOR:**	[redacted]
**CO-AUTHOR:**	[redacted]

Document History

Date	Version number	Protocol/ Study number	Summary of changes
29-May-2024	1.0	ARCT-2303-01 ver 2.0	First SAP
23-Aug-2024	2.0 (Amendment 1.0)	ARCT-2303-01 ver 2.0	Use the terms Reportable, Exclusionary, Other Reportable (non-exclusionary) PDs for analysis of PDs, and update how to list the Reportable PDs, and specify the Exclusionary PDs for PP (Day 29) and PP (Day 181) will be reported for first and second final analyses, respectively.<br>ANCOVA model will be adjusted by the stratification factors from CRF, not IRT.<br>Add two parameters in the summary of COVID vaccination history<br>- Primary series based on first 2 doses<br>- Name of last vaccine /booster vaccine<br>Specify to summarize data from left arm if any subjects reported solicited local AEs from both arms after Day 29 vaccination, and all solicited local AEs (from both left and right arms) will be presented in the reactogenicity listings.
18-Dec-2024	3.0 (Amendment 2.0)	ARCT-2303-01 ver 2.0	Section 2.1 Study Design: Update of Figure 1, Cohort A.<br>Section 3.1 General Considerations: Clarification for usage of data in case of duplicate eDiary entries which was decided and documented prior to any planned analysis.<br>Section 3.2 Key Definition: Clarification of definition for Prior Medications.<br>Section 3.8 Treatment Groups: Addition of a treatment group for additional analysis.<br>Section 3.9 Subgroup Analysis: Addition of subgroup analysis planned for second final analysis.<br>Section 4.1.4 Per-Protocol (PP) Set: Clarification that PP Day 181 is a randomly selected subset of participants; PP (Day 181) and PP (Day 181) for Cross-Neutralization are defined based on different immunogenicity test results.<br>Section 5.1.2 Analysis Populations: Addition of planned summaries description.<br>Section 5.2 Protocol Deviations: Clarification of reporting of cumulative exclusionary PDs across the entire study at study end.<br>Section 5.3 Demographics and Baseline Characteristics: Specification of the age for studies ARCT-2303-01 and ARCT-154-J01.<br>Section 5.7 Prior and concomitant medications: Clarification of concomitant medications during

			both Day 1 to Day 57 and Day 1 to EOS will be summarized.<br>Section 7.1 Solicited AEs: Addition of summaries for the solicited AEs during 7 days post Day 1 vaccination for Groups 2a and 2b combined, Groups 3a and 3b combined.<br>Handling of implausible data collected in eDiary, and analysis of reported symptoms from different arms on different days for one subject, which were decided and documented prior to any planned analysis.<br>Section 7.2 Unsolicited AEs: Addition of summaries for unsolicited AEs, related unsolicited AEs, and SAE 28 days post Day 1 vaccination for Groups 2a and 2b combined, Groups 3a and 3b combined.<br>Section 8.2 Descriptive Summaries: Removal of proportion of >=LLOQ at Day 181 consistent with the planned analysis.

SAP APPROVAL

The original version of SAP was prepared by Novotech. The author of the original version Maureen Royeca from Novotech was not involved in the amendment 1.0 and amendment 2.0, therefore the signature from Novotech is not required for this SAP 3.0 (Amendment 2.0).

Authorship for SAP 3.0 (Amendment 2.0) with signatures:
By my signature, I confirm that this SAP has been reviewed by Arcturus Therapeutics, Inc. and has been approved for use on the ARCT-2303-01 study:

Name	Role / Title, Company	Signature	Date
[redacted]	[redacted]<br>Arcturus Therapeutics, Inc	[redacted]	
	[redacted]<br>Arcturus Therapeutics, Inc		
	[redacted]<br>Arcturus Therapeutics, Inc		
	[redacted]<br>Arcturus Therapeutics, Inc		

Table of contents

1 INTRODUCTION 9
2 PROJECT OVERVIEW 10
2.1 Study Design 10
2.2 Study Objectives and Endpoints 12
2.3 Sample Size 14
2.4 Randomization 15
2.5 Blinding and Unblinding 15
3 STATISTICAL CONSIDERATIONS 17
3.1 General Considerations 17
3.2 Key Definitions 18
3.3 Inferential Analyses 19
3.4 Multiple Comparisons and Multiplicity Adjustments. 20
3.5 Handling of Missing Data 20
3.6 Conversion of categorical values 20
3.7 Coding of Events and Medications 20
3.8 Treatment Groups 21
3.9 Subgroup Analysis 21
4 ANALYSIS SETS 22
4.1 Population Descriptions 22
5 SUMMARY OF STUDY DATA 24
5.1 Participant disposition and analysis populations 24
5.2 Protocol deviations 24
5.3 Demographic and baseline information 24
5.4 Risk Factors 25
5.5 COVID Vaccination History 25
5.6 Treatment Exposure 25
5.7 Prior and concomitant medications 25
5.8 Medical history 26
5.9 Surgical and Medical Procedure 26
6 EFFICACY 27
7 SAFETY 28
7.1 Solicited Adverse Events 28
7.2 Unsolicited Adverse Events 29
7.3 Vital Signs 30
7.4 12-Lead Electrocardiogram (ECG) and Cardiac Enzyme Testing 30
7.5 Physical Examinations 30
7.6 Pregnancy Test Results 30
7.7 Laboratory Assessment SARS-CoV-2 31
7.8 Laboratory Assessment Safety 31
8 IMMUNOGENICITY 32
8.1 Comparison of immune responses 32

8.2 Descriptive Summaries 34
9 CHANGES TO THE PLANNED ANALYSIS 36
10 FINAL ANALYSES 37
10.1 First Final Analysis 37
10.2 Second Final Analysis 37
11 SOFTWARE 38
12 REFERENCES 39
13 APPENDIX 40
13.1 Sample Size Calculation 40

List of Abbreviations

Abbreviation	Description
AE	Adverse Event
AESI	Adverse Event of Special Interest
ANCOVA	Analysis of Covariance
aQIV	adjuvanted Quadrivalent Influenza Vaccine
ATC	Anatomical Therapeutic Class
BMI	Body Mass Index
CI	Confidence Interval
COVID-19	Coronavirus Disease 2019
CS	Clinically Significant
CSR	Clinical Study Report
CV	Continuous Variable
DBP	Diastolic Blood Pressure
DV	Dependent Variable
ECG	12-Lead Electrocardiogram
eCRF	Electronic Case Report Form
EDC	Electronic Data Capture
EOS	End of Study
ET	Early Termination
FDA	US Food and Drug Administration
GCP	Good Clinical Practice
GMFR	Geometric Mean Fold Rise
GMT	Geometric Mean Titer
HI	Hemagglutination Inhibition
ICF	Informed Consent Form
ICH	International Council for Harmonisation
IRT	Interactive Response Technology
ITT	Intent-to-Treat
IV	Independent Variables
LLOQ	Lower Limit of Quantitation
LS Means	Least-Square Means
MAAE	Medically Attended Adverse Event
MedDRA	Medical Dictionary for Regulatory Activities
mITT	Modified ITT
MN	Microneutralization
N/A	Not Applicable
NI	Noninferiority
NCS	Not Clinically Significant
NK	Not Known
PD	Protocol Deviation
PP	Per Protocol Set
PPS-1	Per-Protocol Set 1
PT	Preferred Term
QIV	Flucelvax Quadrivalent Vaccine
RTSM	Randomisation and Trial Supply Management
RT-PCR	Reverse Transcription Polymerase Chain Reaction
SAE	Serious Adverse Event
SAF	Safety Set
SAP	Statistical Analysis Plan
SARS-CoV-2	Severe Acute Respiratory Syndrome Coronavirus 2

Abbreviation	Description
SBP	Systolic Blood Pressure
SCR	Seroconversion Rate
SD	Standard Deviation
SoA	Schedule of Assessments
SOC	System Organ Class
SOP	Standard Operating Procedure
SRC	Safety Review Committee
TEAE	Treatment Emergent Adverse Event
ULOQ	Upper Limit of Quantitation
US	United States of America
VNA	Virus Neutralizing Assay
WHO-DD	World Health Organization Drug Dictionary

1 INTRODUCTION

The following Statistical Analysis Plan (SAP) provides the outline for the statistical analysis of the data collected from the ARCT-2303-01 study (protocol version *2.0* dated *21 February 2024*).

The analyses will be performed sequentially at two different timepoints (Day 57 and end of study). The first final analysis will be conducted at Day 57 and the second final analysis at the end of the study (see Section 10.1 and Section 10.2 for further details).

The planned analyses identified in this SAP may be included in clinical study reports (CSRs), regulatory submissions, or future manuscripts. Also, post hoc exploratory analyses not necessarily identified in this SAP may be performed to further examine study data. Any post hoc, or unplanned, exploratory analyses performed will be clearly identified as such in the final CSR.

Day 57 analysis includes the analyses of immunogenicity (Day 29), reactogenicity (within 7 days post each vaccination), unsolicited adverse events (AEs reported within 28 days post each vaccination), and SAEs, AESIs, MAAEs, and AEs leading to early termination from the study (Day 1 to Day 57). The final study analysis (Day 181) includes the analyses of immunogenicity (Day 181), and SAEs, AESIs, MAAEs, and AEs leading to early termination from the study from Day 1 to Day 181, so the safety analysis will be partially repeated. The database used for the Day 57 analyses will be “frozen” (i.e. soft lock) therefore any potential data modifications that occurs after that date will be reported (e.g. summaries or listings) at the time of the final analyses.

2 PROJECT OVERVIEW

2.1 Study Design

This is a multicenter, observer-blind, randomized, controlled phase 3 study to evaluate the immunogenicity, reactogenicity, and safety of the investigational COVID-19 vaccine (ARCT-2303), administered concomitantly with Quadrivalent Influenza Vaccines or standalone in adults who previously received primary vaccination series and at least one booster dose of the US-authorized mRNA COVID-19 vaccines.

Participants who received at least 3 doses (a 2-dose primary series and at least one booster dose) of the US-authorized mRNA COVID-19 vaccines, with the last mRNA booster dose (original strain or bivalent) ⩾5 months before enrolment will be recruited in one of the two cohorts.

Cohort A (participants 18 to 64 years of age; N=1200)

Individuals will be randomly assigned to one of the three study groups:

- Group 1a (ARCT-2303/ Flucelvax Quadrivalent, N=400): participants will receive one dose of ARCT-2303 and one dose of Flucelvax Quadrivalent (opposite arms) on Day 1, and one dose of placebo on Day 29.
- Group 2a (ARCT-2303, N=400): participants will receive one dose of ARCT-2303 and one dose of placebo (opposite arms) on Day 1, and one dose of Flucelvax Quadrivalent on Day 29.
- Group 3a (Flucelvax Quadrivalent, N=400): participants will receive one dose of Flucelvax Quadrivalent and one dose of placebo (opposite arms) on Day 1, and one dose of ARCT-2303 on Day 29.

Cohort B (participants ⩾65 years of age; N=480)

Individuals will be randomly assigned to one of the three study groups:

- Group 1b (ARCT-2303/ Fluad Quadrivalent, N=160): participants will receive one dose of ARCT-2303 and one dose of Fluad Quadrivalent (opposite arms) on Day 1, and one dose of placebo on Day 29.
- Group 2b (ARCT-2303, N=160): participants will receive one dose of ARCT-2303 and one dose of placebo (opposite arms) on Day 1, and one dose of Fluad Quadrivalent on Day 29.
- Group 3b (Fluad Quadrivalent, N=160): participants will receive one dose of Fluad Quadrivalent and one dose of placebo (opposite arms) on Day 1, and one dose of ARCT-2303 on Day 29.

In addition, all available serum samples from study participants of study ARCT-154-J01, who received a booster dose of candidate ARCT-154 vaccine according to the protocol, provided pre- and post-vaccination blood samples and did not have evidence of SARS-CoV-2 infection (no COVID-19 infections were reported between Day 1 and Day 29) and major protocol deviations, will be selected to use as a comparator. This set of samples will compose up to 385 individuals 18 years of age and above, who were included in the per-protocol set 1 (PPS-1; Day 29 primary analysis from final 12 months analyses database). All PPS-1 subjects who received ARCT-154 were selected from analysis datasets ADSL, where flag ADSL.PPROTFL = Y. The blood samples at Day 1 and Day 29 for these PPS-1 subjects will be selected and sent out for testing together with the Day 1 and Day 29 samples from ARCT-2303 vaccine recipients (Groups 2a and 2b). These new testing results of immunogenicity data for these samples will be used in this study for assessment of coprimary study objectives 1 and 2.

Figure 1: Study Design

Cohort A (individuals 18 to 64 years of age)

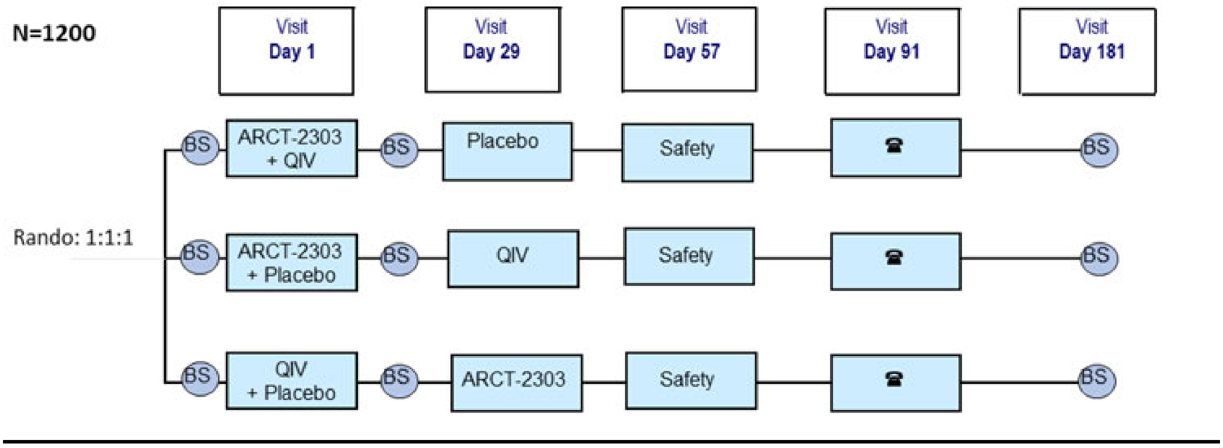

Abbreviations: QIV, Flucelvax Quadrivalent vaccine; BS, blood sample; rando, randomization.

Cohort B (individuals ≥65 years of age)

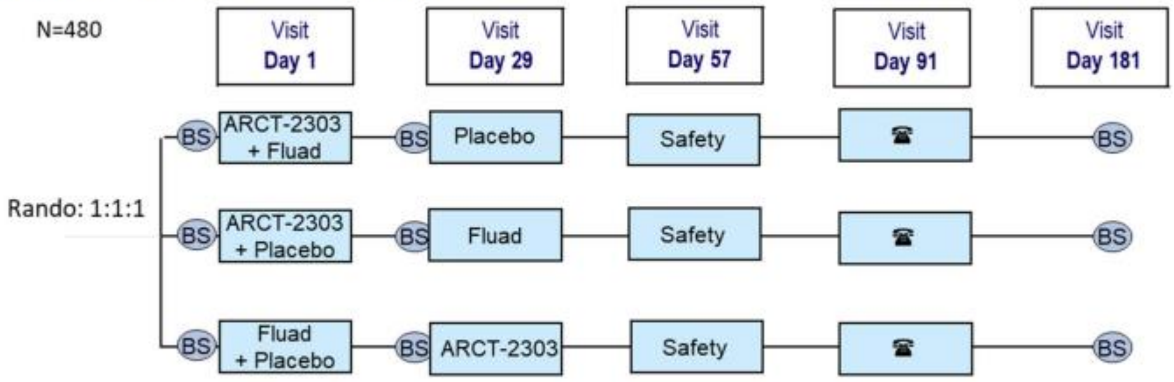

Abbreviations: Fluad, Fluad Quadrivalent vaccine; BS, blood sample; rando, randomization.

A set of participants from study ARCT-154-J01 (second booster dose)

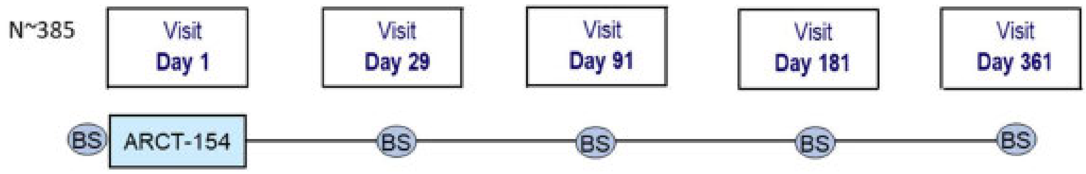

Abbreviation: BS, blood sample.

2.2 Study Objectives and Endpoints

2.2.1 Primary Objectives and Endpoints

The Primary Immunogenicity objectives are:

Objectives	Endpoints
1. To demonstrate that the ARCT-2303 vaccine, when given as a booster dose, elicits an immune response that is superior (simple superiority[a]) to those after a booster dose of ARCT-154 (in study ARCT-154-J01), as measured by GMTs of neutralizing antibodies against Omicron XBB.1.5 subvariant.	SARS-CoV-2 neutralizing antibody titers against Omicron XBB.1.5 subvariant on Day 29, in:<br>• ARCT-2303 vaccine recipients (Groups 2a and 2b)<br>• ARCT-154-J01 study participants who received a booster dose of ARCT-154 vaccine
2. To demonstrate that the ARCT-2303 vaccine, when given as a booster dose, elicits an immune response that is noninferior to those after a booster dose of ARCT-154 (in study ARCT-154-J01), as measured by SARS-CoV-2 neutralizing antibody seroconversion rate with Omicron XBB.1.5 subvariant	SARS-CoV-2 neutralizing antibody seroconversion rates against Omicron XBB.1.5 subvariant on Day 29, in:<br>• ARCT-2303 vaccine recipients (Groups 2a and 2b)<br>• ARCT-154-J01 study participants who received a booster dose of ARCT-154 vaccine
3. To demonstrate that vaccination with Flucelvax Quadrivalent, when given concomitantly with ARCT-2303, elicits an immune response that is noninferior to that of Flucelvax Quadrivalent, when given standalone, as measured by serum Hemagglutination Inhibition (HI) assay[b], at 28 days after vaccination	HI assay titers against influenza vaccine strains on Day 29 in:<br>• Co-admin group recipients (Group 1a)<br>• Standalone Flucelvax Quadrivalent group recipients (Group 3a)
4. To demonstrate that vaccination with ARCT-2303, when given concomitantly with Flucelvax Quadrivalent, elicits an immune response that is noninferior to that of ARCT-2303, when given standalone, as measured by GMTs of neutralizing antibodies against Omicron XBB.1.5 subvariant, at 28 days after vaccination.	SARS-CoV-2 neutralizing antibody responses against Omicron XBB.1.5 subvariant on Day 29, in:<br>• Co-admin group recipients (Group 1a)<br>• Standalone ARCT-2303 group recipients (Group 2a)

[a] As per FDA guidance: “simple” superiority (margin of >1-fold for GMT ratio). US FDA Guidance for Industry. Emergency Use Authorization for Vaccines to Prevent COVID-19. Document issued on March 31, 2022.
[b] In case of lack of agglutination for a specific strain using HI assay, immunogenicity for that strain will be assessed as measured by microneutralization (MN) assay as an acceptable alternative.

2.2.2 Secondary Objectives and Endpoints

The Secondary Immunogenicity objectives are:

Objectives	Endpoints
1. To demonstrate that the ARCT-2303 vaccine, when given as a booster dose, elicits an immune response that is superior (super superiority[a]) to those after a booster dose of ARCT-154 (in study ARCT-154-J01), as measured by GMTs of neutralizing antibodies against Omicron XBB.1.5 subvariant	SARS-CoV-2 neutralizing antibody titers against Omicron XBB.1.5 subvariant on Day 29, in:<br>• ARCT-2303 vaccine recipients (Groups 2a and 2b)<br>• ARCT-154-J01 study participants who received a booster dose of ARCT-154 vaccine
2. To demonstrate that the ARCT-2303 vaccine, when given as a booster dose, elicits an immune response that is superior to those after a booster dose of ARCT-154 (in study ARCT-154-J01), as measured by SARS-CoV-2 neutralizing antibody seroconversion rate with Omicron XBB.1.5 subvariant	SARS-CoV-2 neutralizing antibody seroconversion rates against Omicron XBB.1.5 subvariant on Day 29, in:<br>• ARCT-2303 vaccine recipients (Groups 2a and 2b)<br>• ARCT-154-J01 study participants who received a booster dose of ARCT-154 vaccine
3. To assess the immunogenicity of ARCT-2303 vaccine when administered with or without Flucelvax Quadrivalent, as measured by virus neutralization assay	SARS-CoV-2 neutralizing antibody responses against Omicron XBB.1.5 subvariant on Days 1, 29, and 181 (a subset of samples):<br>• Co-admin group recipients (Group 1a)<br>• Standalone ARCT-2303 group recipients (Group 2a)
4. To assess the immunogenicity of Flucelvax Quadrivalent when administered with or without ARCT-2303 as measured by HI assay	HI assay titers against influenza vaccine strains on Day 1 and Day 29, in:<br>• Co-admin group recipients (Group 1a)<br>• Standalone Flucelvax Quadrivalent group recipients (Group 3a)
5. To assess the immunogenicity of ARCT-2303 vaccine, when administered with or without Fluad Quadrivalent, as measured by virus neutralization assay, in participants ⩾65 years of age.	SARS-CoV-2 neutralizing antibody responses against Omicron XBB.1.5 subvariant on Days 1, 29, and 181:<br>• Co-admin group recipients (Group 1b)<br>• Standalone ARCT-2303 group recipients (Group 2b).

6. To assess the immunogenicity of Fluad Quadrivalent when administered with or without ARCT-2303 as measured by HI assay, in participants ⩾65 years of age	HI assay titers against influenza vaccine strains on Day 1 and Day 29, in:<br>• Co-admin group recipients (Group 1b)<br>• Standalone Fluad Quadrivalent group recipients (Group 3b).

[a] As per FDA guidance: "super" superiority (margin of >1.5-fold for GMT ratio). US FDA Guidance for Industry. Emergency Use Authorization for Vaccines to Prevent COVID-19. Document issued on March 31, 2022.

The Secondary Safety objective is:

Objective	Endpoints
1. To assess the safety and reactogenicity of the study vaccines when given in co-administration or standalone	• Local and systemic AEs reported within 7 days after each study vaccination<br>• Unsolicited AEs reported within 28 days after each vaccination<br>• SAE, AEs leading to early termination from study, MAAEs, and AESIs during the entire study period (6-month follow-up)

2.2.3 Exploratory Objectives and Endpoints

Objective	Endpoints
1. To assess the immune response elicited by the ARCT-2303 vaccine against a panel of SARS-CoV-2 strains, as measured by VNA	SARS-CoV-2 neutralizing antibody responses with a panel of historical and new emergent SARS-CoV-2 strains on Days 1 and 29 in a subset of:<br>• Standalone ARCT-2303 group recipients (Group 2a and/or 2b)<br>• Standalone QIV group recipients (Group 3a and/or 3b)
2. To assess the immunogenicity of influenza vaccine(s) when administered with or without ARCT-2303 as measured by microneutralization assay (in a subset of participants)	Microneutralization (MN) titers against influenza vaccine strains on Day 1 and Day 29, in:<br>• Co-admin group recipients (a subset of participants from Group 1a and/or 1b)<br>• Standalone QIV group recipients (a subset of participants from Group 3a and/or 3b).

2.3 Sample Size

A total sample size of approximately 1680 participants (1200 participants 18 to 64 years of age, and 480 participants ⩾65 years of age), and a sample size of 385 participants of study ARCT-154-J01 (PPS-1) is proposed for the study objectives. A full discussion of the sample size calculation can be found in the Section 13.

2.4 Randomization

After signing the informed consent form (ICF) each participant will be given a Participant ID according to the screening order. On Day 1, following confirmation of eligibility, a randomization number will be assigned, and participants will be allocated to treatment using an interactive response technology (IRT), Medidata Rave Randomisation and Trial Supply Management (RTSM).

On Day 1, within each age cohort (A and B), participants will be randomly assigned at a 1:1:1 ratio into three study groups:

Cohort A (participants 18 to 64 years of age; approximately 1200 participants)

- Group 1a (ARCT-2303/Flucelvax Quadrivalent, N=400): participants will receive one dose of ARCT-2303 and one dose of Flucelvax Quadrivalent (opposite arms) on Day 1, and one dose of placebo on Day 29.
- Group 2a (ARCT-2303, N=400): participants will receive one dose of ARCT-2303 and one dose of placebo (opposite arms) on Day 1, and one dose of Flucelvax Quadrivalent on Day 29.
- Group 3a (Flucelvax Quadrivalent, N=400): participants will receive one dose of Flucelvax Quadrivalent and one dose of placebo (opposite arms) on Day 1, and one dose of ARCT-2303 on Day 29.

Cohort B (participants ⩾65 years of age; approximately 480 participants)

- Group 1b (ARCT-2303/Fluad Quadrivalent, N=160): participants will receive one dose of ARCT-2303 and one dose of Fluad Quadrivalent (opposite arms) on Day 1, and one dose of placebo on Day 29.
- Group 2b (ARCT-2303, N=160): participants will receive one dose of ARCT-2303 and one dose of placebo (opposite arms) on Day 1, and one dose of Fluad Quadrivalent on Day 29.
- Group 3b (Fluad Quadrivalent, N=160): participants will receive one dose of Fluad Quadrivalent and one dose of placebo (opposite arms) on Day 1, and one dose of ARCT-2303 on Day 29.

Randomization will be stratified based on their COVID-19 vaccination history:

1) Total number of COVID-19 vaccine doses
    a. 3 doses
    b. 4+ doses
2) Composition of the last booster dose
    a. original strain
    b. bivalent/XBB1.5
    c. unknown.

The stratification groups are designed to be balanced based on actual N of participants in each group, and the maximum percent of participants in each stratification group is not pre-specified.

2.5 Blinding and Unblinding

This study will be observer-blind.

Due to the visual differences between the study vaccines, the participants will be blinded before receiving the study vaccinations. An unblinded dosing team, not involved with study participant's evaluation, will prepare and administer the study vaccine doses. The study vaccine syringe will be opacified to avoid unblinding of the participant.

The administration of the study vaccine will also be performed behind a closed curtain to ensure that the other blinded staff members do not become unblinded. The investigative study center personnel, as well as the sponsor personnel involved in the monitoring or conduct of the study, will be blinded to the study vaccine code.

The laboratories in charge of immunogenicity and safety testing will also be blinded, so associating the sample with an assigned treatment or study visit will not be possible.

The treatment code should be broken only if the investigator/physician in charge of the participant feels that the case cannot be treated without knowing the identity of the study vaccine. Except in cases of medical necessity, a participant’s treatment should not be unblinded without the approval of the sponsor. If unblinding should occur (by either accidental unblinding or emergency unblinding) before completion of the study, the investigator must promptly contact the sponsor or designee and document the date and reason that the blind was broken in the source documentation and eCRF. Instructions regarding emergency unblinding will be provided to the investigator.

All personnel involved in the study will remain fully blinded until the first final analysis (Day 57). For Day 57 analysis, immunogenicity and safety data will be reported on a group level; no individual listings will be generated. Access to participant-level information about study groups will be restricted to specified personnel involved in the statistical analysis.

The blinded site staff, CROs (not involved in the statistical analysis), and sponsor representatives (not involved in the statistical analysis/writing of CSR) will remain blinded until final database lock.

3 STATISTICAL CONSIDERATIONS

Statistical analyses and generations of Tables, Listings and Figures (TLFs) will be handled and processed per the sponsor's representative (Cytel) Standard Operating Procedures (SOPs), which are written based on the principles of Good Clinical Practice (GCP).

3.1 General Considerations

Data collected on the electronic case report form (eCRF) will be presented in the data listings for the second analysis (study end) and will be listed and sorted by cohort, treatment group, participant number and visit, where applicable. No individual listings will be generated at the first analyses (Day 57).

All safety and descriptive summaries will be presented by the treatment group and nominal visit/time point (where applicable). All disposition and concomitant medication descriptive summaries will be presented by treatment group.

Unless otherwise stated, the following methods will be applied:

- Continuous variables: Descriptive statistics will include the number of non-missing values (n), arithmetic mean, standard deviation (SD), median, minimum, and maximum values.

  The minimum and maximum values will be displayed to the same decimal precision as the source data, the arithmetic mean, SD, and median values will be displayed to one more decimal than the source data for the specific variable.

  95% Confidence Intervals (CIs), mean differences (among treatments and from baseline) and least-square (LS-Means) values will be displayed to one more decimal than the source data for a specific variable.

  The appropriate precision for derived variables will be determined based on the precision of the data on which the derivations are based, and statistics will be presented in accordance with the above-mentioned rules.

  For source data with > 5 decimals, rounding to a maximum of 3 decimals will be performed.

- Categorical variables: Descriptive statistics will include counts and percentages per category. The denominator in all percentage calculations will be the number of participants in the relevant analysis population with non-missing data, unless specifically stated otherwise. Percentages will be displayed to one decimal place. Proportions will be displayed to 3 decimal places.

  95% Confidence Intervals (CIs), differences, and other categorical parameters will be displayed to one decimal place for percentages. 95% Confidence Intervals (CIs) and differences in proportions will be displayed to 3 decimal places.

- Repeat/unscheduled assessments: Only values collected at scheduled study visits/time points will be presented in summary tables. If a repeat assessment was performed, the result from the original assessment will be presented as the result at the specific visit/time point (early termination visits will not be summarized). All collected data will be included in the data listings.
- Duplicate entries in eDiary: For duplicate daily entries in the electronic diary with at least one different value collected between 2 entries, the last data entered will be considered for the analysis (rule established before any planned analysis).
- Assessment windows: All assessments will be included in the data listings and no visit windows will be applied to exclude assessments that were performed outside of the protocol specified procedure windows. For safety summaries (where applicable), the table below defines the analysis time windows that correspond to the targeted visit.

Visit (target day)	Visit window (days)
Study Day 1: Screening and Vaccination 1	-7
Study Day 29: Follow-up Visit 1 and Vaccination 2	± 3
Study Day 57: Follow-up Visit 2	± 3
Study Day 91: Safety Call	± 7
Study Day 181: Final Visit (EOS)	± 7

- Result display convention: Results will be center aligned in all summary tables and listings. Participant identifiers visit and parameter labels may be left-aligned if required.
- Date and time display conventions: The following display conventions will be applied in all outputs where dates and/or times are displayed:

  Date only: YYYY-MM-DD

  Date and time: YYYY-MM-DD HH:MM

If only partial information is available, unknown components of the date or time will be presented as 'NK' (not known), i.e., '2016-NK-NK'. Times will be reported in military time.

3.2 Key Definitions

The following definitions will be used:

- Baseline: The baseline value is defined as the last available valid (quantifiable continuous or categorical value), non-missing observation for each participant prior to first study vaccine administration. If an assessment is taken on the day of first study vaccine administration, but the time is not available, this assessment will be considered for baseline assessment. Repeat and unscheduled assessments will be included in the derivation of the baseline values.
- Change from Baseline: The change from baseline value is defined as the difference between the result collected/derived at a post-baseline visit/time point and the baseline value.

  The change from baseline value at each post-baseline visit/time point will be calculated for all continuous parameters using the following formula:

  Change from Baseline Value = Result at Visit/Time Point – Baseline Value

  The change from baseline value will only be calculated if the specific post-baseline visit/time point result and the baseline value for the parameter are both available and will be treated as missing otherwise.

- Study day: The study day of an event is defined as the relative day of the event starting with the date of the first study vaccine administration (reference date) as Day 1 (there will be no Day 0).

  The study day of events occurring before the first study vaccine administration will be calculated as:

  Study Day = (Date of Event - Date of First Study Vaccine Administration)

  For events occurring on or after Day 1, study day will be calculated as:

  Study Day = (Date of Event - Date of First Study Vaccine Administration) + 1

  Study days will only be calculated for events with complete dates and will be undefined for events that are 'Ongoing' at the time of database lock (either Day 57 or end of study).

Relative days compared to an alternative reference point will be calculated similarly, but the alternative starting reference start date will be used instead of the date of the first study vaccine dosing.

- Prior Medications: Prior medications are defined as any medication stopped prior to the first administration of the study vaccine.
- Concomitant Medications: Concomitant medications are defined as any medication (other than the study vaccine) that was used at least once after the first administration of the study vaccine. Medications that were stopped on the same date as the first study vaccine administration will be defined as concomitant medications. If a clear determination cannot be made (partial medication end dates) the medication will be classified as concomitant
- Treatment Emergent Adverse Events (TEAEs) are defined as adverse events that occurred following the first administration of study medication. Adverse events without enough evidence to identify whether it occurred before or after the first administration will be considered as a TEAE.

3.3 Inferential Analyses

3.3.1 Hypotheses

A sequential, unidirectional testing procedure is planned to account for multiplicity, i.e., hypotheses in step 2 will only be tested if hypotheses in step 1 are successfully proven. Therefore, the total 5% Type I error (alpha) can be allocated to the step 2 testing, and so on. Within each step, a parallel testing strategy is applied, i.e., the hypotheses will be tested simultaneously at a 5% alpha level (2-sided), and a successful demonstration of all hypotheses within the step is required to continue to the next step.

The following hypotheses will be tested:

STEP 1 - Parallel testing of H_{01} and H_{02}

H_{01}: Day 29 $GMT_{ARCT\text{-}2303\ booster\ dose}$ /Day 29 $GMT_{ARCT\text{-}154booster\ dose} \leqslant 1.0$

H_{02}: Day 29 $SCR_{ARCT\text{-}2303\ booster\ dose}$ - Day 29 $SCR_{ARCT\text{-}154booster\ dose} \leqslant -5.0\%$

STEP 2 - Parallel testing of H_{03} and H_{04}

H_{03}: Day 29 GMT QIV $_{QIV+ARCT\text{-}2303}$ /Day 29 GMT QIV $_{QIV+Placebo} \leqslant 0.67$

H_{04}: Day 29 GMT ARCT-2303 $_{QIV+ARCT\text{-}2303}$ /Day 29 GMT ARCT-$2303_{ARCT\text{-}2303+Placebo} \leqslant$ 0.67

STEP 3 – Sequential Testing of H_{05} then H_{06}

H_{05}: Day 29 SCR $_{ARCT\text{-}2303\ booster\ dose}$ - Day 29 SCR $_{ARC\text{-}154booster} \leqslant 0\%$

H_{06}: Day 29 GMT $_{ARCT\text{-}2303\ booster\ dose}$ /Day 29 GMT $_{ARC\text{-}154booster} \leqslant 1.5$

See for more details, Section 10.1 of the protocol.

3.3.2 Analysis of covariance

Analysis of covariance (ANCOVA) is a general linear model that evaluates whether the means of a dependent variable (DV) are equal across levels of one or more categorical independent variables (IV) and across one or more continuous variables (CV). Mathematically, ANCOVA decomposes the variance in the DV into variance explained by the CV(s), variance explained by the categorical IV, and residual variance (Keppel, G. (1991)).

The GMT ratio will be assessed using the ANCOVA model with treatment as factor and baseline antibody titer level (log-scale), age and sex as covariates, adjusted for the stratification factors from the CRF: number of previous doses and composition of last booster, if applicable. Two-sided 95% CIs for GMT ratio will be obtained by taking the anti-log of the confidence limits for

the adjusted mean difference of the logarithmically transformed assay results which is calculated using the t-distribution.

The general code to be executed in SAS for ANCOVA is as follows:

ODS OUTPUT LSMeanDiffCL = *output_data_name*;

PROC GLM DATA=*input_data*;

class Treatment Factor* sex**;

model LogValue=Treatment Covariate# Factor* sex**/solution;

lsmeans Treatment/pdiff cl;

run;

**sex as a categorical variable (Male, Female)

#Covariate: age as a continuous variable, baseline antibody titers in log scale

*stratification factors (not applicable when comparing the historical control group)

1. number of previous COVID-19 vaccine doses (3 vs 4+)
2. composition of the booster dose (original strain, bivalent/XBB1.5, unknown).

3.4 Multiple Comparisons and Multiplicity Adjustments.

No adjustment for multiplicity is planned for this study due to the hierarchical testing strategy.

3.5 Handling of Missing Data

For the classification of Treatment emergent adverse event (TEAE) and Concomitant medication, the following will be applied in the following order:

a. If all dates/times (start and stop) are missing, the event/medication will automatically be classified as a TEAE/Concomitant medication.
b. For AEs with a missing start date/time, if the event end date/time is prior to first study vaccine administration, the event will not be classified as a TEAE.
c. If only the AE start year/ medication end year is present and is the same or is after the first study vaccine administration year unit, the event/medication will be classified as a TEAE/Concomitant medication.
d. If the AE start month and year/medication end month and year are present and are the same or after the first study vaccine administration month and year units, the event/medication will be classified as a TEAE/Concomitant medication.

Missing results will not be imputed for all the immunogenicity and safety analyses.

3.6 Conversion of categorical values

In some safety assessments, continuous variables are expressed as a range (i.e. < 10). In such cases, values may be converted to the range boundary (upper or lower limit as applicable). As an example, a value of <10 may be converted to 10. Such substitutions will be clearly documented in the footnotes of relevant outputs.

For the calculation of GMT and GMFR, imputation will be applied. Antibody titer values above the upper limit of quantitation (ULOQ) will be set to ULOQ, while antibody titer values below the lower limit of quantitation (LLOQ) will be imputed as ½ LLOQ (i.e., 0.5*LLOQ).

3.7 Coding of Events and Medications

Medical history, Surgical and Medical Procedure and AE verbatim terms will be coded using the Medical Dictionary for Regulatory Activities (MedDRA) using the latest version available at

the time of study commencement. Terms will be coded to the full MedDRA hierarchy, but the system organ class (SOC) and preferred terms (PT) will be of primary interest for the analysis.

Prior and concomitant medications will be coded using the World Health Organization Drug Dictionary (WHO-DD) using the latest version available at the time of study commencement. Medications will be mapped to the full WHO-DD Anatomical Therapeutic Chemical (ATC) class hierarchy, but PTs will be of primary interest in this analysis.

3.8 Treatment Groups

Group 1a: ARCT-2303 + QIV

Group 2a: ARCT-2303 + Placebo

Group 3a: QIV + Placebo

Group 1b: ARCT-2303 + aQIV

Group 2b: ARCT-2303 + Placebo

Group 3b: aQIV + Placebo

ARCT-2303 (Gr 2a and 2b)

QIV/aQIV (Gr 3a and 3b)

ARCT-154

If any actual treatments received cannot belong to any one of the treatment groups above (e.g. subject receives only one injection), the participant will be summarized under the randomized treatment group.

3.9 Subgroup Analysis

Subgroup analysis for selected parameters including immunogenicity, solicited AEs, and unsolicited AEs will be performed for the second Final Analysis.

The following subgroups will be assessed for subgroup analysis (last 2 subgroups will be used for immunogenicity data only):

- Gender: Male or Female
- Race: White and Asian
- BMI: <30 and ⩾30 kg/m^2
- Country: Australia, Philippines, Costa Rica and Honduras
- Ethnicity: Hispanic or Latino, not Hispanic or Latino
- Vaccine composition of last dose: ancestral, bivalent (BA.1 or BA.5 containing)
- Number of last COVID-19 doses: 3 doses or >=4 doses

The descriptive summaries will be presented for subgroup analysis: Groups 2a and 2b combined vs Groups 3a and 3b combined for safety data; Groups 2a and 2b combined for immunogenicity data.

4 ANALYSIS SETS

In this study 6 analysis populations are defined: Enrolled Set, Exposed Set / Intent to Treat (ITT) Analysis Set, Modified ITT Analysis Set, Per-protocol (PP) set, Safety analysis set (SAF) and Historical control set.

Furthermore, any additional exploratory analysis not identified in the SAP will be identified in the final CSR as exploratory post hoc analyses, including analyses for additional study populations or subgroups of interest.

All disposition and demographic data analysis will be based on the ITT population. All listings will be presented by the ITT population (or Enrolled Set for disposition).

4.1 Population Descriptions

The evaluation of participant’s inclusion or exclusion to Day 29 analysis population will be done prior data base lock. The evaluation of participant's inclusion or exclusion to Day 181 analysis population will be done prior to final unblinding.

4.1.1 Enrolled Set

All screened participants who provided informed consent, received a participant ID, and were randomized.

4.1.2 Exposed Set / Intent to Treat (ITT) Analysis Set

All participants who received at least one dose of a study vaccine.

The ITT Analysis Set will be analyzed according to the vaccine assigned.

4.1.3 Modified ITT (mITT) Analysis Set

All participants who are randomized, received at least one dose of a study vaccine and provided at least one non-missing post-baseline immunogenicity result. mITT analyses will not be conducted for Day 181.

The mITT will be analyzed according to vaccine assigned.

4.1.4 Per-protocol (PP) set

Participants in the mITT who correctly received all protocol-required doses of study vaccines at Day 1 and who at or prior to the specified timepoint (Day 29 and Day 181) have no evidence of SARS-CoV-2 infection and no protocol deviations impacting the analysis of immunogenicity data. As PP Set for Day 181 is a subset population, these rules are only applicable for the subset. Two PP sets for Day 181 are defined: one is PP (Day 181) and the other one is PP (Day 181) for Cross-Neutralization. Participants in PP (Day 181) need to have Omicron XBB.1.5 results available at Day 181, and participants in PP (Day 181) for Cross-Neutralization need to have cross-neutralization results available at Day 181.

The PP set will be analyzed according to the vaccine assigned.

The following reportable protocol deviations are expected to affect the immunogenicity and will lead to exclusion of a participants from the PPS:

- No informed consent obtained
- Deviations related to dose and administration
- Randomization error/code broken
- Received non-study COVID-19 vaccine
- Received prohibited concomitant medication/other vaccines
- Deviations related to immunogenicity samples up to analysis time point (e.g. no blood sample taken, blood sample taken out of window, blood sample not taken/treated in accordance with lab manual).
- Underlying medical condition forbidden by the protocol or which may influence the immune response

4.1.5 Safety analysis set (SAF)

All participants in the Exposed Set who provide any evaluable post-vaccination reactogenicity and/or safety data (including VS and ECG data).

Should there be an error in administration where the actual study vaccine that the participant received was different than the one to which he/she was assigned, the Safety Analysis Set will be analyzed according to the vaccine actually received.

Note: in case of incorrect vaccine received, the actual vaccine received will be provided after database lock by unblinded monitor.

4.1.6 Historical control set (ARCT-154-J01)

All participants from study ARCT-154-J01 who received a booster with ARCT-154 vaccine, provided evaluable pre- and post-vaccination blood samples, and did not have SARS-CoV-2 infection and protocol deviations that impact on immunogenicity assessment. These participants were included in the per-protocol set 1 for the primary immunogenicity analysis.

5 SUMMARY OF STUDY DATA

5.1 Participant disposition and analysis populations

Participant disposition and analysis population analysis will be based on the Enrolled Set. Participant disposition and analysis populations will be summarized descriptively as described in section 3.1 (categorical descriptive analysis).

5.1.1 Participant Disposition

Participant disposition will include the number of participants who complete the full course of study treatment, participants who did not complete treatment, reason treatment was not completed, participants who completed the study, participants withdrawn from the study, as well as the primary reason for early discontinuation. Participant disposition will be summarized descriptively overall and by group.

5.1.2 Analysis Populations

The number of participants included in each analysis set will be summarized descriptively.

Reasons for exclusions from mITT, PP Day 29, and PP Day 181, including corresponding exclusionary PDs for PP Day 29 and PP Day 181, will be summarized accordingly.

In addition, the inclusion/exclusion of each participant into/from each of the defined analysis sets will be presented in the by-participant data listings.

5.2 Protocol deviations

Reportable protocol deviations (PD), including Exclusionary PDs and Other Reportable (non-Exclusionary) PDs, will be summarized for Day 1 to Day 57 and for Day 1 to End of Study (EOS) by treatment group for the ITT analysis set.

For first final analysis, Exclusionary PDs for PP (Day 29) and Other Reportable PDs up to the date of data lock for this analysis will be reported. For second final analysis, all Exclusionary PDs and Other Reportable PDs for Day 1 to End of Study will be reported.

All Reportable protocol deviations will be presented for each participant in by-participant data listings (for Day 1 to Day 57 and Day 1 to EOS).

Protocol deviations will be reviewed by medical monitors and categorized as noted in the Project Specific Protocol Deviation Specifications, including assessment of severity (major or minor).

Prior to database lock protocol deviations and analysis sets will be reviewed and confirmed to decide which subjects and/or subject data will be excluded from certain analyses. Decisions regarding exclusion of subjects and/or data from analyses will be documented and approved prior to database lock.

5.3 Demographic and baseline information

Demographic and baseline information analysis will be presented for the ITT and PP analysis sets.

Demographic and baseline information will be summarized descriptively overall and by treatment group, as described in section 3.1.

Demographics

The following demographic and baseline parameters will be analyzed:

<u>Continuous descriptive analysis:</u>

Age (years)

Height (cm)

Weight (kg)

BMI (kg/m^2)

Categorical descriptive analysis:

Age Category (18-64 years, 65+ years)

Sex

Race

Ethnicity

Country (derived by using the first digit of the site number)

Similarly, the available demographic and baseline parameters will be summarized for ARCT-154 recipients (study ARCT-154-J01) too.

For study ARCT-2303-01 participants, age collected on CRF is used for the analysis, while age at the time of first vaccination is considered for study ARCT-154-J01 participants.

5.4 Risk Factors

Risk factors for severe COVID-19 at enrolment will be summarized descriptively overall and by study group for the ITT analysis set, as described in section 3.1.

5.5 COVID Vaccination History

COVID vaccination history data will be summarized by treatment groups for the ITT analysis set:

- Subject received at least 3 doses of vaccine (last dose >=5 months prior to enrolment) (Yes, No)
- Number of COVID-19 vaccine doses
- Name of licensed COVID-19 vaccine
- Primary series based on first two doses (derived)
- Vaccine composition of last dose
- Name of last vaccine/booster vaccine

COVID vaccination history responses will be presented in the by-participant data listings. A listing comparing stratification factors as per CRF v. IRT will be generated.

5.6 Treatment Exposure

All study vaccine administration information on Day 1 and Day 29, including study vaccine administered (Yes/No), date and time of administration, location (left arm/right arm) will be presented in the by-participant data listings. Information on diary dispensation, activation and training will also be included in the same listings.

Number and percentage of participants who received each vaccine will be summarized by study group for the Safety analysis set, as described in section 3.1. Contraindications for the Second Vaccination will be presented in the by-participant data listings. Vaccination errors (or mis-dose) will be listed.

5.7 Prior and concomitant medications

Prior and concomitant medications for Day 1 to Day 57, as well as concomitant medications from Day 1 to EOS will be summarized for the Safety analysis set by Anatomical Therapeutic Chemical (ATC) class Level 2 and PT as noted in section 3.1 (categorical descriptive analysis). Participant who used the same medication on multiple occasions will only be counted once in the specific category. ATC Level 2 and PTs will be sorted alphabetically. In addition to the summaries by the coded terms, the number of participants who used at least one concomitant medication during the study will be presented.

Prior and Concomitant medications will be presented in the by-participant data listings.

5.8 Medical history

Medical history will be coded using MedDRA® and will be summarized by treatment groups, SOC and PT, for the Safety analysis set.

Medical history data will be presented in the by-participant data listings.

5.9 Surgical and Medical Procedure

Surgical and medical procedure as collected on the “Surgical and Medical Procedures” CRF page will be presented in the by-participant data listings.

6 EFFICACY

No efficacy analysis is planned for this study.

7 SAFETY

Safety endpoints will be analyzed using the Safety analysis set and will be summarized descriptively as described in section 3.1.

7.1 Solicited Adverse Events

Information on Solicited Adverse Events will be summarized descriptively by treatment group, as described in section 3.1.

- Solicited local reactions and systemic AEs will be recorded daily within 7 days after each study vaccination:
    - Local reactions: pain, erythema and swelling at the injection site.
    - Systemic AEs: fatigue, headache, myalgia, arthralgia, nausea, dizziness, chills, and fever.

Severity grading for these AEs is presented in Table 1. Severity will be categorized programmatically as Mild, Moderate and Severe based on data collected on the eDiary and applying the definition provided in Table 1.

Table 1 Severity Grading for Solicited Local Reactions and Systemic Adverse Events

	**Mild**	**Moderate**	**Severe**
Pain	No interference with daily activities	Interferes with daily activities	Prevents daily activity
Erythema	25-50 mm	51-100 mm	> 100 mm
Swelling	25-50 mm	51-100 mm	> 100 mm
Fatigue	No interference with daily activities	Interferes with daily activities	Prevents daily activity
Headache	No interference with daily activities	Interferes with daily activities	Prevents daily activity
Myalgia	No interference with daily activities	Interferes with daily activities	Prevents daily activity
Arthralgia	No interference with daily activities	Interferes with daily activities	Prevents daily activity
Nausea	No interference with daily activities	Interferes with daily activities	Prevents daily activity
Dizziness	No interference with daily activities	Interferes with daily activities	Prevents daily activity
Chills	No interference with daily activities	Interferes with daily activities	Prevents daily activity
Fever (°C)	38.0-38.4	38.5-38.9	⩾39.0

Note: Based on Food and Drug Administration (FDA) Guidance Document: Toxicity Grading Scale for Healthy Adult and Adolescent Volunteers Enrolled in Preventive Vaccine Clinical Trials.

The following parameters will be summarized by treatment group using descriptive statistics:

- Time to onset of event (first occurrence)
- Duration of event: that is the max duration for those events that have no interruptions during the 7 days (eg if event is on day 1, on day 3, day 4 and day 5 then duration is 3 days. Duration will be calculated considering the stop date from the AEs CRF page for those events that last beyond day 7)

The Solicited AEs will be summarized by treatment group using percentages of participants, overall and by severity (i.e. tables will include any severity, Mild, Moderate and Severe)

- Solicited AEs during 7 days after each vaccination
- Solicited AEs during 7 days after each vaccination overall (any) and by maximum severity (mild, moderate, severe)

Additionally, the following summaries will be provided for Groups 2a and 2b combined (ARCT-2303), Groups 3a and 3b combined (QIV/aQIV):

- Solicited AEs during 7 days post Day 1 vaccination
- Solicited AEs during 7 days post Day 1 vaccination by reaction
- Solicited AEs during 7 days post Day 1 vaccination by grade
- Solicited AEs during 7 days post Day 1 vaccination by reaction and grade

Implausible data values reported by the study participants in the electronic diary will be excluded from analysis (rule established before any planned analysis).

Implausible values are defined as:

- A measurement >200 mm for any local injection site erythema and swelling.
- A body temperature of <33°C and >43°C.

For the reported solicited local AEs from both arms after Day 29 vaccination: If a subject reports symptoms from different arms on different days, the symptoms should be combined; If a subject reports symptoms from both arms on the same day, only the data from the left arm will be analyzed and reported in the summary tables, and all solicited local AEs (from both left and right arms) will be presented in the reactogenicity listings (rule established before any planned analysis).

The following will be summarized by treatment groups:

- Use of antipyretics and analgesics will be summarized by frequency and percentage of participants reporting use.
- Body temperature will be summarized by 0.5°C increments from 38.0°C up to ≥40.0°C.

7.2 Unsolicited Adverse Events

All unsolicited AEs including SAEs, MAAEs and AESIs will be coded using MedDRA. Medical occurrences that begin before the study vaccination but after obtaining informed consent will be recorded in the medical history/current medical conditions section of the eCRF, not the AE section, all unsolicited AEs collected on the AE CRF page following the first administration will be considered TEAEs and will be included in the summaries.

Summary tables will include the number of participants (%) experiencing an event along with the number of events. Participants will be counted only once for each SOC and PT level (categorical descriptive analysis). All TEAE summaries will be presented by study group, SOC and PT.

The TEAE summaries will include:

- Overview summary of unsolicited AEs (Day 1 – Day 57)
- Summary of unsolicited AEs reported within 30 minutes after each study vaccination (derived by onset date time if event after vaccination date time) by SOC and PT. Please note that for Day 1, when 2 vaccinations are given, the 30 minutes interval is from the second vaccination.
- Summary of unsolicited AE within 28 days after each study vaccination by SOC and PT.
- Summary of related unsolicited AEs within 28 days after each study vaccination by SOC and PT
- Summary of unsolicited AE within 28 days after each study vaccination by SOC, PT and severity.
- Summary of related unsolicited AEs within 28 days after each study vaccination by SOC, PT and severity
- Summary of SAEs by SOC and PT
- Summary of related SAEs by SOC and PT
- Summary of SAEs by SOC, PT and severity
- Summary of related SAEs by SOC, PT and severity
- Summary of MAAEs by SOC and PT
- Summary of related MAAEs by SOC and PT
- Summary of AESIs by SOC and PT
- Summary of related AESIs by SOC and PT
- Summary of AEs leading to discontinuation from study by SOC and PT
- Summary of related AEs leading to discontinuation from study by SOC and PT
- Summary of (S)AEs with fatal outcome by SOC and PT

Additionally, the following summaries up to 28 days post Day 1 vaccination will be provided for Groups 2a and 2b combined (ARCT-2303), Groups 3a and 3b combined (QIV/aQIV):

- Summary of unsolicited AEs by SOC and PT
- Summary of related unsolicited AEs by SOC and PT
- Summary of SAEs by SOC and PT

For final analysis, summary of SAEs, MAAEs, AESI, AE leading to discontinuation and death will be presented cumulatively (Day 1 to EOS).

7.3 Vital Signs

The following vital signs measurements will be taken at the time points specified in the Schedule of Assessments (refer to the Protocol):

- Systolic blood pressure (SBP) (mmHg)
- Diastolic blood pressure (DBP) (mmHg)
- Heart Rate (beats/min);
- Respiratory rate (breaths/min)
- Body Temperature (ºC)

All vital signs data collected at scheduled and unscheduled visits will be included in the listings, but only results collected at scheduled visits will be included in the summary tables. Summary tables including results from Day 1, Day 29 and Day 57 will be included in the first analysis and results at Day 181 will be included in the final analysis.

The parameter names that will be used in the outputs will comprise of the test name and the unit of measure, for example, ‘Systolic Blood Pressure (mmHg)’. Parameters will be sorted in the order that the measurements were collected in on the Vital Signs eCRF page within the tables and listings.

Vital signs measurements will present summary statistics for the results at the baseline and each scheduled post-baseline visit for each of the parameters. In addition, summaries will be presented for the change from baseline values at each scheduled post-baseline visit (continuous descriptive analysis).

The decimal precision to which the summaries for each parameter will be presented will be based on the maximum number of decimals to which the results were reported on the eCRF.

7.4 12-Lead Electrocardiogram (ECG) and Cardiac Enzyme Testing

A baseline 12-lead ECGs at baseline will be performed for study participants to identify potential asymptomatic myocarditis and pericarditis cases.

In case of suspected myocarditis or pericarditis cases during the study period, detailed evaluation, including ECG, cardiac enzyme testing, and other relevant data should be evaluated.

Additional ECG evaluation at unscheduled visits is at the investigator’s discretion.

12-Lead ECG and cardiac enzyme testing results will be presented in the by-participant data listings.

7.5 Physical Examinations

By-participant data listings will be created for all physical examination parameters and all time points.

7.6 Pregnancy Test Results

All information related to pregnancy testing (urine and serum based) will be presented in the by-participant data listings.

7.7 Laboratory Assessment SARS-CoV-2

Results of SARS-CoV-2 rapid antigen testing and any additional SARS-CoV-2 RT-PCR testing will be presented in the by-participant data listings.

7.8 Laboratory Assessment Safety

NA.

8 IMMUNOGENICITY

All Immunogenicity analyses will be done using the PP set. For secondary immunogenicity objectives 3, 4, 5 and 6, if more than 5% participants are excluded from PP set as compared to mITT set, then the analysis will be repeated for the mITT analyses set.

Humoral immune responses against Omicron XBB.1.5 subvariant will be assessed by means of Virus Neutralizing assay (VNA) in Groups 1a and 1b, Groups 2a and 2b, and in samples (Days 1 and 29) from ARCT-154 recipients, included in the PPS-1 in study ARCT-154-J01.

Humoral immune responses against QIV and aQIV will be assessed by means of Hemaggluination Inhibition assay (H1N1, H3N2, two B strains, separately) will be assessed in Groups 1a and 1b, and Groups 3a and 3b. In case of lack of agglutination for a specific strain using HI assay, immunogenicity for that strain will be assessed as measured by microneutralization assay as an acceptable alternative.

The per protocol (PP) set will be used for the primary objectives to assess superiority and noninferiority. PPS and PPS1 will be used for primary objectives 1 and 2, whereas PPS will be used for primary objectives 3 and 4.

The GMT ratio for each of the primary immunogenicity endpoints will be assessed by using analysis of covariance (ANCOVA) model that will include covariates and stratification factors (see Section 3.3.2). Two-sided 95% CIs for GMT ratio will be obtained by taking the antilog of the confidence limits for the adjusted mean difference of the logarithmically transformed assay results which is calculated using t-distribution.

Descriptive immunogenicity analysis including GMT, GMFR, and SCR will be produced for each group at all available time points, along with the 95% CIs.

Geometric mean will be calculated as the mean of the antibody results after the data are log-transformed and then taking the antilog of the log-mean to present the results on the original scale. Two-sided 95% CI will be obtained by taking the log transformation of the antibody results and calculated based on t-distribution and then taking the anti-log of the confidence limit. For the calculation of GMT and GMFR, imputation will be applied to antibody titer values above the upper limit of quantitation; ULOQ will be used. Antibody titer values below the lower limit of quantitation will be imputed as ½ LLOQ (i.e., 0.5*LLOQ).

GMFR analysis will include participants with antibody results available at both baseline (prior to dose) and post-vaccination. It will be calculated as the mean of the difference after log-transformed results (post baseline minus baseline for each participant) and exponentiating the mean. Two-sided 95% CI will be obtained by taking the log transform of the antibody results and calculating the 95% CI based on t-distribution for the mean difference after the data are log-transformed, then taking the anti-log of the confidence limit. Percentage of participants with seroconversion will be summarized along with the 95% CI by the Clopper-Pearson method (Clopper C, Pearson ES (1934)).

Seroconversion is defined as binary variable for participants with non-missing values at pre-vaccination and post-vaccination as:

= 1, if there is a post-vaccination titer ⩾4xLLOQ for pre-vaccination titer <LLOQ

= 1, if there is at least 4-fold increase (post-vaccination) from pre-vaccination titer ⩾LLOQ

= 0, otherwise

An alternative SCR is also defined if there is at least a 4-fold increase (post vaccination) from pre-vaccination titer.

8.1 Comparison of immune responses

Hypothesis testing will be done in a sequential manner, and all the success criteria at each step need to be met to continue to the next step.

STEP 1: Co-Primary Objectives 1 and 2

- Simple Superiority of immune response of ARCT-2303 compared to ARCT-154 to the Omicron XBB.1.5 subvariant based on GMT ratio
- Noninferiority of immune response of ARCT-2303 compared to ARCT-154 against the Omicron XBB.1.5 subvariant based on the SCR

↓

STEP 2: Co-Primary Objectives 3 and 4

- Noninferiority of immune response of co-administration of ARCT-2303 and Flucelvax Quadrivalent compared to standalone Flucelvax Quadrivalent against influenza vaccine strains based on the HI assay
- Noninferiority of immune response of co-administration of ARCT-2303 and Flucelvax Quadrivalent compared to ARCT-2303 against Omicron XBB.1.5 subvariant based on GMT ratio

↓

STEP 3a: Secondary Objective 2

Superiority of immune response of ARCT-2303 compared to ARCT-154 against the Omicron XBB.1.5 subvariant based on the SCR

↓

STEP 3b: Secondary Objective 1

Super Superiority of immune response of ARCT-2303 compared to ARCT-154 against the Omicron XBB.1.5 subvariant based on GMT ratio

8.1.1 Superiority for the Omicron XBB.1.5 subvariant on Day 29 based on GMT

Immunological superiority will be demonstrated by calculating the GMT ratio and the corresponding 95% CI of the SARS-CoV-2 neutralizing antibody titers against Omicron XBB.1.5 subvariant for ARCT-2303 vaccine recipients and ARCT-154-J01 study participants:

$$\text{GMT ratio} = \frac{\text{GMT}_{\text{ARCT}-2303(\text{Groups 2a and 2 b})(\text{Day 29})}}{\text{GMT}_{\text{ARCT}-154(\text{booster})\ (\text{Day 29})}}$$

The analysis of the GMT ratio will be done using ANCOVA, as described in Section 3.3.2. Simple superiority will be concluded if the lower limit of the 2-sided 95% CI for the GMT ratio is higher than 1.0.

8.1.2 Noninferiority for the Omicron XBB.1.5 subvariant on Day 29 based on the SCR

SARS-CoV-2 neutralizing antibody seroconversion rates against Omicron XBB.1.5 subvariant for ARCT-2303 vaccine recipients and ARCT-154-J01 study participants will also be compared:

$$\text{SCR difference} = \text{SCR}_{\text{ARCT-2303 (Groups 2a and 2b)}} - \text{SCR}_{\text{ARCT-154 (booster)}}$$

Seroconversion rate is defined as the percentage of participants with a ≥4-fold increase in titer from that at Day 1 (or from LLOQ if Day 1 titer <LLOQ).

The seroconversion rate and its two-sided 95% CI for each group will be calculated using the Clopper-Pearson method. Age group and sex will be applied to calculate the SCR difference based on MH method. Noninferiority will be concluded if the lower limit of the 2-sided 95% CI for SCR difference is higher than - 5% (i.e., minus 5%).

8.1.3 Noninferiority for the influenza vaccine strains on Day 29 based on the HI assay

If simple superiority of the GMT and noninferiority of the SCR are concluded in the 2 tests discussed above, immune response for each influenza vaccine strain based on the HI assay

after co-administration of ARCT-2303 and Flucelvax Quadrivalent will be compared to immune response after standalone administration of Flucelvax Quadrivalent

$$\text{GMT ratio} = \frac{\text{GMT}_{\text{QIV+ ARCT−2303(Day 29)}}}{\text{GMT}_{\text{QIV+Placebo (Day 29)}}}$$

The analysis of the GMT ratio will be done using ANCOVA, as described in Section 3.3.2. Non-inferiority will be concluded if the lower limit of the 2-sided 95% CI for the GMT ratio is higher than 0.67 for all 4 influenza strains.

8.1.4 Noninferiority for the Omicron XBB.1.5 subvariant on Day 29 for GMT

Together with the noninferiority of immune response for influenza vaccine strains, SARS-CoV-2 neutralizing antibody responses against Omicron XBB.1.5 subvariant on Day 29 after co-administration of ARCT-2303 and Flucelvax Quadrivalent will be compared to immune response after standalone administration of ARCT-2303

$$\text{GMT ratio} = \frac{\text{GMT}_{\text{QIV+ ARCT−2303(Day 29)}}}{\text{GMT}_{\text{ARCT−2303+Placebo (Day 29)}}}$$

The analysis of the GMT ratio will be done using ANCOVA, as described in Section 3.3.2. Non-inferiority will be concluded if the lower limit of the 2-sided 95% CI for the GMT ratio is higher than 0.67.

8.1.5 Super superiority for the Omicron XBB.1.5 subvariant on Day 29 for GMT

Using the same analysis as Section 8.1.1, super superiority of ARCT-2303 to ARCT-154 in terms of neutralizing antibodies GMTs against Omicron XBB.1.5 subvariant will be demonstrated if the lower limit of the 2-sided 95% CI for GMT ratio is higher than 1.5.

8.1.6 Superiority for the Omicron XBB.1.5 subvariant on Day 29 for the difference of SCR

Using the same analysis as Section 8.1.2, superiority of ARCT-2303 to ARCT-154 in terms of neutralizing antibodies SCR against Omicron XBB.1.5 subvariant will be demonstrated if the lower limit of the 2-sided 95% CI for difference of SCRs is higher than 0%.

8.2 Descriptive Summaries

Results from VNA assay will also be summarized using descriptive statistics for the following parameters:

- Geometric mean titer (GMT) on Days 1, 29 and 181;
- Geometric mean-fold rise (GMFR) as increases of the post-vaccination titer over the pre-vaccination titer;
- Seroconversion rate (SCR) on Day 29 as the percentage of participants in each arm with either:
    - A pre-vaccination titer below the lower limit of quantitation (LLOQ) and a post-vaccination titer ≥4xLLOQ; or
    - A pre-vaccination titer ≥LLOQ and a ≥4-fold increase in post-vaccination titer.
- Proportion of participants with antibody titer ≥LLOQ on Days 1 and 29.

The results of HI and MN will be summarized using descriptive statistics for the following parameters:

- Geometric mean titer (GMT) on Days 1 and 29.
- Geometric mean-fold ratio (GMFR) as increases of the post-vaccination titer over the pre-vaccination titer.
- SCR on Day 29 as the percentage of participants in a group with either:
    - A pre-vaccination titer below the LLOQ for the respective assay and a post-vaccination titer ≥4xLLOQ.
    - A pre-vaccination titer ≥ LLOQ and a ≥4-fold increase in post-vaccination titer.
- Proportion of participants with HI titer ≥1:10, 1:20, 1:40, 1:80, 1:160 and 1:320 on Days 1 and 29.

- Proportion of participants with MN titer ≥1:10, 1:20, 1:40, 1:80, 1:160 and 1:320 on Days 1 and 29.

There is no formal hypothesis testing for evaluating the co-administration of ARCT-2303 and Fluad Quadrivalent. The results of the analysis will be presented as described above.

9 CHANGES TO THE PLANNED ANALYSIS

Not applicable.

10 FINAL ANALYSES

The results of this study will be released sequentially in two separate final analyses.

The two final analyses will be based on the final version of the SAP. Any deviations from the planned analysis will be documented in the CSR.

10.1 First Final Analysis

The first final analysis will include all immunogenicity data (up to Day 29), reactogenicity data (7 days post-vaccination), unsolicited AEs (28 days post-vaccination), and safety data collected from Day 1 to Day 57 and associated primary and secondary objectives. This analysis will be conducted on cleaned and frozen data. The results of this analysis will be presented in the initial clinical study report (CSR). Immunogenicity and safety data will be reported on a group level only. No individual listings will be generated. Access to participant-level information about study groups will be restricted to specified personnel involved in the statistical analysis.

10.2 Second Final Analysis

The second final analysis of immunogenicity and safety data collected from Day 57 to the study end will be performed as soon as all data are available. For SAE, MAAE, AESI and AEs leading to discontinuation that will be reported cumulatively (ie from Day 1 to end of study as well). The results of this analysis will be presented in the final CSR. All individual data listings with information on participant study group will be presented in the addendum to the CSR.

11 SOFTWARE

- SAS® Version 9.4 or higher (SAS Institute, Cary, North Carolina, USA).

12 REFERENCES

ICH guidelines E3, E6, E9.

Clinical Study Protocol Version 2.0 dated *21 February 2024*.

Emergency Use Authorization for Vaccines to Prevent COVID-19 Guidance for Industry Document issued on March 31, 2022

Keppel, G. (1991). Design and analysis: A researcher’s handbook (3rd ed.). Englewood Cliffs: Prentice-Hall, Inc.

Clopper C, Pearson ES (1934). The use of confidence or fiducial limits illustrated in the case of the binomial. Biometrika. 1934;26 (4): 404–413.

Miettinen OS, Nurminen M. (1985). “Comparative analysis of two rates.” Statistics in Medicine 4:213-226 (1985).

13 APPENDIX

13.1Sample Size Calculation

<u>STEP 1</u>

Objective 1:

The coprimary objective of simple superiority of the ARCT-2303 vaccine booster (groups 2a and 2b) compared to the ARCT-154 booster for the Omicron XBB.1.5 subvariant

Superiority of $GMT_{ARCT\text{-}2303\ (booster)}$ to $GMT_{ARCT\text{-}154\ (booster)}$

The superiority margin for the Omicron XBB.1.5 subvariant is defined as 1.0 for the GMTs ratio, thus the lower limit of the 2-sided 95% CI for GMT ratios (ARCT-2303/ARCT-154) is higher than 1.0.

Statistical power for the coprimary objective 1 is calculated assuming that:

a. the GMT ratio between ARCT-2303 (Day 29) and ARCT-154 (booster; ARCT-154-J01 study) (Day 29) groups is 1.5,
b. the common standard deviation is expected to be 0.60 in the log10 scale.

If the GMT ratio is 1.5 that means that the difference of the 2 means in log scale is 0.176 (δ). The differences between those two means is equal to 0 (=SM) when the GMT ratio is equal to 1.

Numeric Results for an Equal-Variance T-Test

Solve For:	Power
Difference:	δ = μ1 – μ2 = Mt – Mr
Higher Means Are:	Better
Hypotheses:	H0: δ ⩽ SM vs. H1: δ > SM

Power	N1	N2	N	SM	δ	σ	Alpha
0.98768	500	350	850	0	0.176	0.6	0.025

Power	The probability of rejecting a false null hypothesis when the alternative hypothesis is true.
N1 and N2	The number of items sampled from each population.
N	The total sample size. N = N1 + N2.
SM	The magnitude of the margin of superiority. Since higher means are better, this value is positive and is the distance above the reference mean that is required to be considered superior.
□	The difference between the treatment and reference means at which power and sample size calculations are made. □ = μ1 – μ2 = Mt – Mr.
σ	The assumed population standard deviation for each of the two groups.
Alpha	The probability of rejecting a true null hypothesis. One-sided Alpha=0.025 that is equivalent to Two-sided Alpha=0.05

The null (H0) and alternative (H1) hypotheses for one-sided tests are defined as

$H0$: $\mu1 – \mu2 \leqslant \delta0$ versus $H1$: $\mu1 – \mu2 > \delta0$
or equivalently

$H0$: $\delta \leqslant \delta 0$ versus $H1$: $\delta > \delta 0$.

Rejecting this test implies that the mean difference is larger than the value $\delta 0$.

Superiority by a Margin tests are special cases of the above directional tests. It will be convenient to adopt the following specialized notation for the discussion of these tests.

Parameter Interpretation
$\mu 1$: Mean of population 1. Population 1 is assumed to consist of those who have received the new vaccine.
$\mu 2$: Mean of population 2. Population 2 is assumed to consist of those who have received the reference vaccine.
MS (SM): Margin of superiority. This is a tolerance value that defines the magnitude of difference. This may be thought of as the smallest difference (>=0).
δ: is the value of $\mu 1 - \mu 2$, the difference between the means. This is the value at which the power is calculated.

Note that the actual values of $\mu 1$ and $\mu 2$ are not needed. Only their difference is needed for power and sample size calculations.

Superiority by a Margin Tests

A superiority by a margin test tests that the treatment mean is better than the reference mean by more than the superiority margin. The actual direction of the hypothesis depends on the response variable being studied.

In this case, higher values are better. The hypotheses are arranged so that rejecting the null hypothesis implies that the treatment mean is greater than the reference mean by at least the margin of superiority.

The value of δ at which power is calculated must be greater than $|MS|$. The null and alternative hypotheses with $\delta 0 = |MS|$ are

$H0$: $\mu 1 \leqslant \mu 2 + |MS|$ versus $H1$: $\mu 1 > \mu 2 + |MS|$
$H0$: $\mu 1 - \mu 2 \leqslant |MS|$ versus $H1$: $\mu 1 - \mu 2 > |MS|$

$H0$: $\delta \leqslant |MS|$ versus $H1$: $\delta > |MS|$

Objective 2

The coprimary noninferiority of the ARCT-2303 vaccine booster (groups 2a and 2b) compared to the ARCT-154 booster for the Omicron XBB.1.5 subvariant

Noninferiority (NI) of $SCR_{ARCT\text{-}2303\ (booster)}$ - $SCR_{ARCT\text{-}154}$

Statistical power for the coprimary objective 2 is calculated assuming that:

a. the difference between SCR in ARCT-2303 (Day 29) and SCR in ARCT-154 (booster; ARCT154-J01 study) (Day 29) groups is expected to be >10%
b. the SCR is equal to 50% in the ARCT-154 group

Non-Inferiority Tests for the Difference Between Two Proportions

Numeric Results

Solve For:	Power
Difference:	Z-Test with Unpooled Variance
Hypotheses:	H0: P1 – P2 ⩽ δ0 vs. H1: P1 – P2 > δ0

Power	N1	N2	N	Ref. P2	P1|H0 P1.0	P1|H1 P1.1	NI Diff δ0	Diff δ1	Alpha
0.99136	500	350	850	0.5	0.45	0.6	-0.05	0.1	0.025

* Power was computed using the normal approximation method.

Power	The probability of rejecting a false null hypothesis when the alternative hypothesis is true.
N1 and N2	The number of items sampled from each population.
N	The total sample size. N = N1 + N2.
P2	The proportion for group 2, which is the standard, reference, or control group.
P1	The proportion for group 1, which is the treatment or experimental group.
P1.0	The smallest group 1 proportion that still yields a non-inferiority conclusion. P1.0 = P1|H0.
P1.1	The proportion for group 1 under the alternative hypothesis at which power and sample size calculations are made. P1.1 = P1|H1.
□0	The non-inferiority difference under H0. Δ0 = P1.0 – P2.
□1	The non-inferiority difference assumed by the alternative hypothesis, H1. Δ1 = P1.1 – P2.
Alpha	The probability of rejecting a true null hypothesis. One-sided Alpha=0.025 that is equivalent to Two-sided Alpha=0.05

The two populations from which dichotomous (binary) responses are analyzed (the higher proportions are better) are investigational and control. The probability of response in population 1 (the new vaccine group: investigational) is $p1$ and in population 2 (the reference group) is $p2$. Random samples of $n1$ and $n2$ individuals are obtained from these two populations. The data from these samples can be displayed in a 2-by-2 contingency table as follows.

	Success	Failure	Total
Investigational	x11	x12	n1
Control	x21	x22	n2
Totals	m1	m2	N

The binomial proportions, $pp1$ and $pp2$, are estimated from these data using the formulae

$\hat{p}1 = x11/n1$

$\hat{p}2 = x21/n2$

Let $p1.0$ represent the group 1 proportion tested by the null hypothesis, $H0$. The power of a test is computed at a specific value of the proportion which we will call $p1.1$. Let $\delta0$ represent the smallest difference (margin of non-inferiority that in our case is 0.05) between the two proportions that still results in the conclusion that the investigational agent is not inferior to the reference. For a non-inferiority test, $\delta0 < 0$. The set of statistical hypotheses that are tested is

$H0$: $p1 – p2 \leqslant \delta0$ versus $H1$: $p1 – p2 > \delta0$, which can be rearranged to give

$H0$: $p1 \leqslant p2 + \delta0$ versus $H1$: $p1 > p2 + \delta0$

The most direct is to simply give values for $p2$ and $p1.0$. However, it is often more meaningful to give $p2$ and then specify $p1.0$ implicitly by specifying the difference.

Parameter	Computation	Hypotheses
Difference	$\delta 0 = p1.0 - p2$	$H0$: $p1 - p2 \leqslant \delta 0$ versus $H1$: $p1 - p2 > \delta 0$

Non-Inferiority

For coprimary objective 2 the assumption is that SCR is 50% in the reference method ($p2 = 0.50$). If the SCR of the investigational is no less than 5 percentage points worse ($\delta 0 = -0.05$) than the reference, it will be considered non-inferior. Substituting these figures into the statistical hypotheses gives

$H0$: $p1 - p2 \leqslant -0.05$ versus $H1$: $p1 - p2 > -0.05$

The overall power for STEP 1 is calculated assuming that coprimary objectives 1 and 2 are independent; therefore overall power is 0.979 (0.98768*0.99136) as stated in the protocol (section 10.2)

A sample size of up to 385 participants of study ARCT-154-J01, who received ARCT-154 vaccine booster, provided evaluable pre- and post-vaccination blood samples, and did not have SARS-CoV-2 infection and protocol deviations that impact on immunogenicity assessment (PPS-1), and up to 560 participants in the ARCT-2303 (groups 2a and 2b) provides approximately 99% power (n=350 and n=500 in the two groups if there is drop out of approximately 10%) to demonstrate each of the 2 coprimary objectives; therefore, there is at least 97% overall power to demonstrate the two coprimary objectives, 1 and 2.

STEP 2

If coprimary objectives 1 and 2 are met, then the second step will be assessing the noninferiority of immune response after co-administration and standalone administration of ARCT-2303 and Flucelvax Quadrivalent vaccines. The following null hypotheses will be tested:

H03: Day 29 GMT $QIV_{QIV+ARCT-2303}$ /Day 29 GMT $QIV_{QIV+Placebo} \leqslant 0.67$
(Group 1a vs 3a, i.e. co-admin group recipients vs standalone ARCT-2303 recipients)

H04: Day 29 GMT $ARCT\text{-}2303_{QIV+ARCT-2303}$ /Day 29 GMT $ARCT\text{-}2303_{ARCT-2303+Placebo} \leqslant$ 0.67 (Group 1a vs 2a, i.e. co-admin group recipients vs standalone Flucelvax recipients)

Statistical power is calculated assuming that:

a. GMT ratios are expected to be 1.0
b. the common standard deviation is expected to be 0.60 in the log10 scale.

Two-Sample T-Tests for Non-Inferiority Assuming Equal Variance

Solve For:	Power
Difference:	δ = μ1 – μ2 = Mt – Mr
Higher Means Are:	Better
Hypotheses:	H0: δ ⩽ -NIM vs. H1: δ > -NIM

Power	N1	N2	N	-NIM	δ	σ	Alpha
0.97578	360	360	720	-0.176	0	0.6	0.025

Power	The probability of rejecting a false null hypothesis when the alternative hypothesis is true.
N1 and N2	The number of items sampled from each population.
N	The total sample size. N = N1 + N2.

-NIM	The magnitude and direction of the margin of non-inferiority. Since higher means are better, this value is negative and is the distance below the reference mean that is still considered non-inferior.
□	The difference between the treatment and reference means at which power and sample size calculations are made. $\Delta = \mu 1 - \mu 2 = Mt - Mr$.
σ	The assumed population standard deviation for each of the two groups.
Alpha	The probability of rejecting a true null hypothesis.

No adjustment for multiplicity is planned, as both H03 and H04 (all 5 null hypotheses: 4 strains for QIV and 1 strain for ARCT-2303) must be rejected to declare noninferiority of immune response.

The overall power for STEP 2 is calculated assuming that objectives 3 and 4 are independent therefore overall power is 0.885 (0.97578 to the power of 5) as stated in the protocol (section 10.2)

A sample size of 400 participants per group (groups 1a, 2a, and 3a) provides 97.5 power (n=360 per group if there is drop out is approximately 10%) to demonstrate noninferiority for each one of the 5 strains (4 strains for influenza vaccine and 1 strain for ARCT-2303); therefore, there is at least 88% overall power to demonstrate all of the noninferiority objectives.

The overall power to demonstrate all 4 coprimary objectives is at least 85% (0.979*0.885=0.86).

STEP 3

If objectives 1, 2, 3 and 4 are met, then secondary objectives 2 (superiority of SCR) and 1 ('super superiority' of GMTs) of the ARCT-2303 vaccine, when given as a booster dose compared to a booster dose of ARCT-154 (in study ARCT-154-J01), will be tested in a sequential manner as follows (if H05 is rejected then H06 is tested too):

H05: Day 29 SCR ARCT-2303 booster dose minus Day 29 SCR ARC-154booster $\leqslant$ 0%

H06: Day 29 GMT ARCT-2303 booster dose /Day 29 GMT ARC-154booster $\leqslant$ 1.5

No adjustment for multiplicity is planned: H05 will be tested only if H01,02,03,04 are rejected and H06 will be tested only if H01,02,03,04,05 are rejected. No statistical power is calculated for STEP 3 as it includes only secondary objectives.
