# Supplementary material for: Immunogenicity and safety of self-amplifying mRNA COVID-19 vaccine (ARCT-2303), with or without co-administration of seasonal inactivated influenza vaccine in adults: a phase 3, randomised, controlled, observer-blind, multicentre study
Source: eClinicalMedicine. 2025 Aug 20;87:103428. doi: 10.1016/j.eclinm.2025.103428 (PMC12396474; doi:10.1016/j.eclinm.2025.103428)

**Supplementary material**

**Study schematic 2**

**Study outcomes 3**

**Inclusion and Exclusion criteria 4**

**Toxicity Grading Scale for adverse events 5**

**Influenza vaccine compositions 7**

**Immunology assays 8**

**Sample size justification 9**

**GMTs, GMFR and Seroconversion rates against Omicron XBB 1.5.6 by gender 10**

**GMTs, GMFR and Seroconversion rates against Omicron XBB 1.5.6 by country 11**

**GMFR at Days 29 and 181 against variant strains 12**

**Supplementary figure 1**. Schematic figure of the study design

**Cohort A (individuals aged 18 to 64 years)**


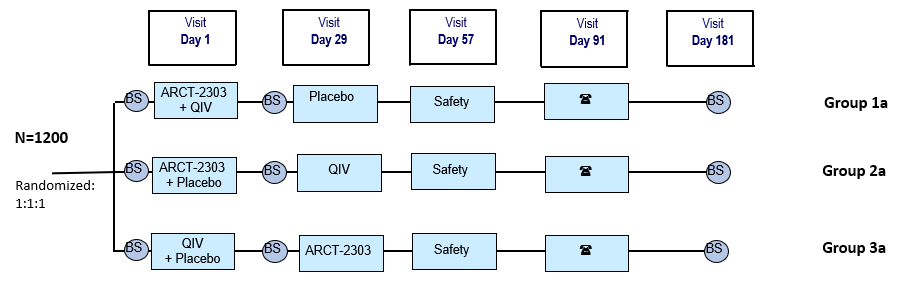


Abbreviations: BS, blood sample; QIV, Flucelvax Quadrivalent vaccine; rando, randomisation.

**Cohort B (individuals aged ≥65 years)**


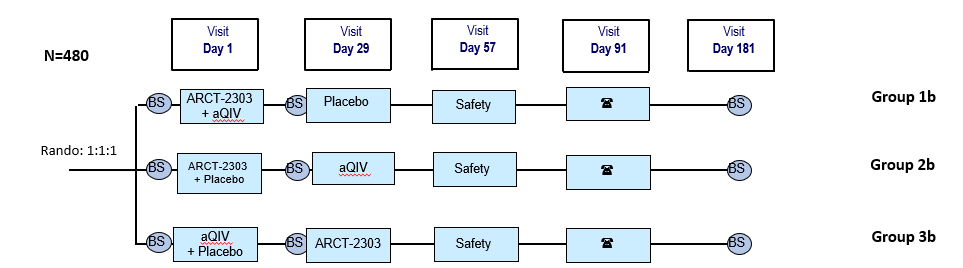


Abbreviations: BS, blood sample; aQIV, adjuvanted quadrivalent influenza vaccine; rando, randomisation.

**Supplementary table 1. Study outcomes**

Primary Outcomes

| **Measure** | **Measure Description** | **Time Frame** |
| --- | --- | --- |
| SARS-CoV-2 neutralizing antibody titers | Immune response as measured by geometric mean titers (GMTs) of neutralizing antibodies against Omicron XBB.1.5 subvariant (Groups 2a and 2b; a comparator group from a previous study) | Day 29 |
| SARS-CoV-2 neutralizing antibody seroconversion rates | Immune response as measured by SARS-CoV-2 neutralizing antibody seroconversion rates against Omicron XBB.1.5 subvariant (Groups 2a and 2b; a comparator group from a previous study) | Day 29 |
| Hemagglutination Inhibition (HI) titers | Immune response as measured by GMTs against influenza vaccine strains (Group 1a; Group 3a) | Day 1, Day 29 |
| SARS-CoV-2 neutralizing antibody titers | Immune response as measured by GMTs of neutralizing antibodies against Omicron XBB.1.5 subvariant (Group 1a; Group 2a) | Day 29 |

Secondary Outcomes

| **Outcome Measure** | **Measure Description** | **Time Frame** |
| --- | --- | --- |
| SARS-CoV-2 neutralizing antibody titers | GMTs of SARS-CoV-2 neutralizing antibody titers against Omicron XBB.1.5 subvariant (Groups 2a and 2b; a comparator group from a previous study) | Day 29 |
| SARS-CoV-2 neutralizing antibody seroconversion rates | SARS-CoV-2 neutralizing antibody seroconversion rates against Omicron XBB.1.5 subvariant (Groups 2a and 2b; a comparator group from a previous study) | Day 29 |
| SARS-CoV-2 neutralizing antibody response (Group 1a; Group 2a) | SARS-CoV-2 neutralizing antibody responses against Omicron XBB.1.5 subvariant as measured by GMT, Geometric Mean Fold Rise (GMFR, post/pre-vaccination), proportion of participants with seroconversion (SCR) and proportion of participants with antibody titer ≥ lower limit of quantitation (LLOQ) (Group 1a; Group 2a) | Days 1, 29 and 181 |
| Hemagglutination Inhibition (HI) titers | HI assay titers against influenza vaccine strains as measured by GMT, GMFR (post/pre-vaccination), proportion of participants with SCR and proportion of participants with HI titers ≥1:40 (Group 1a; Group 3a) | Day 1, Day 29 |
| SARS-CoV-2 neutralizing antibody responses | SARS-CoV-2 neutralizing antibody responses against Omicron XBB.1.5 subvariant as measured by GMT, GMFR (post/pre-vaccination), proportion of participants with SCR, and proportion of participants with antibody titer ≥ LLOQ (Group 1b; Group 2b) | Day 181 |
| Hemagglutination Inhibition (HI) assay titers | HI assay titers against influenza vaccine strains as measured by GMT, GMFR (post/pre-vaccination), proportion of participants with SCR and proportion of participants with HI titers ≥1:40 (Group 1b; Group 3b) | Day 29 |
| Local and systemic adverse events (AEs) | Proportion of participants with local and systemic solicited AEs | Day 1 to Day 8 |
| Unsolicited AEs | Proportion of participants with unsolicited AEs | Day 1 to Day 29 |
| SAE, Medically Attended AEs, AEs of Special Interest, and AEs leading to early termination | Proportion of participants with SAE/MAAE/AESI/AE leading to early termination from the study | Day 1 to Day 181 |

**Inclusion Criteria**

Participants were eligible to be included in the study only if all of the following criteria applied:

1. Male, female, or transgender adults ≥18 years of age

2. Healthy or with pre-existing stable medical conditions

3. Freely provided and documented informed consent prior to any study procedures

4. Previously vaccinated with COVID-19 vaccines as follows:

• Received at least 3 doses (a 2-dose primary series and at least one booster dose) of the US-authorized mRNA COVID-19 vaccines

• US-authorized mRNA COVID-19 vaccine (original strain or bivalent) as last booster dose, administered ≥5 months prior to enrollment

• Documented COVID-19 vaccination

5. Agree to comply with all study visits and procedures

6. Individuals of childbearing potential willing to adhere to protocol contraceptive requirements and local regulations

**Exclusion Criteria**

Participants were to be excluded from the study if any of the following criteria applied:

1. Acute medical illness or febrile illness, including temperature ≥100.4°F (≥38.0°C; measured by any method) within 3 days prior to randomization, with the opportunity to enter the study after fever and illness stabilization, or if COVID-19 had been ruled out

2. Positive SARS-CoV-2 rapid antigen test at screening

3. History of COVID-19 or virologically confirmed SARS CoV 2 infection within the past 5 months or history of COVID-19 with ongoing sequelae

4. Any medical, neurological, or psychiatric condition that, in the opinion of the investigator, could place the participant at an unacceptable risk of injury or render the participant unable to comply with all study procedures and achieve successful completion of the study

5. Known history of severe hypersensitivity reactions, including anaphylaxis, or other significant adverse reactions to any vaccine, any components of mRNA vaccine or influenza vaccine, including egg protein

6. Positive pregnancy test at screening or intention to become pregnant or to breastfeed during the study

7. History of myocarditis, pericarditis, myopericarditis or cardiomyopathy

8. History of Guillain-Barré syndrome, encephalomyelitis, or transverse myelitis

9. Known bleeding disorder that would, in the opinion of the investigator, contraindicate intramuscular (IM) injection

10. History of congenital or acquired immunodeficiency

11. Receipt of immunomodulatory, immunostimulatory, or immunosuppressant drugs including interferon and cytotoxic drugs within 3 months of Screening/Day 1 or planned receipt during the study

12. Requirement for systemic corticosteroids exceeding 10 mg/day of prednisone equivalent for ≥10 days within 30 days of Screening

13. Receipt of immunoglobulins and/or any blood or blood products within the 3 months before the first vaccine administration or planned receipt of such products at any time during the study

14. Immunosuppressive or immunodeficient state, asplenia, or recurrent severe infections

15. Documented history of HIV infection, or currently known to have active tuberculosis

16. Chronic illness that, in the opinion of the investigator, may interfere with study participation or interpretation of study results

17. Individuals receiving treatment with another investigational drug, biological agent, or device within 28 days of screening, or 5 half-lives of the investigational drug, whichever was longer; or currently enrolled in or planning to participate in another clinical study with an investigational agent during the period

18. Individuals who have received any investigational COVID-19 vaccines

19. Receipt of any influenza vaccine within 6 months prior to enrollment or planned receipt of an influenza vaccine during the study period

20. Receipt of any other licensed vaccines within 14 days prior to enrollment in this study or planned receipt of any vaccine up to 14 days after the second study vaccination

21. Investigator site staff members, employees of the sponsor or the clinical research organization (CRO) directly involved in the conduct of the study, or site staff members otherwise supervised by the investigator or immediate family members of any of the previously mentioned individuals

| **Supplementary table 2. Toxicity Grading Scale**  Solicited local reactions and systemic adverse events with severity scale   \|  \| **Mild**  (Grade 1) \| **Moderate**  (Grade 2) \| **Severe**  (Grade 3) \| \| --- \| --- \| --- \| --- \| \| Pain \| No interference with daily activities \| Interferes with daily activities \| Prevents daily activity \| \| Erythema \| 25-50 mm \| 51-100 mm \| > 100 mm \| \| Swelling \| 25-50 mm \| 51-100 mm \| > 100 mm \| \| Fatigue \| No interference with daily activities \| Interferes with daily activities \| Prevents daily activity \| \| Headache \| No interference with daily activities \| Interferes with daily activities \| Prevents daily activity \| \| Myalgia \| No interference with daily activities \| Interferes with daily activities \| Prevents daily activity \| \| Arthralgia \| No interference with daily activities \| Interferes with daily activities \| Prevents daily activity \| \| Nausea \| No interference with daily activities \| Interferes with daily activities \| Prevents daily activity \| \| Dizziness \| No interference with daily activities \| Interferes with daily activities \| Prevents daily activity \| \| Chills \| No interference with daily activities \| Interferes with daily activities \| Prevents daily activity \| \| Fever (°C) \| 38.0-38.4 \| 38.5-38.9 \| ≥39.0 \|   Note: Based on [Food and Drug Administration (FDA) Guidance Document: Toxicity Grading Scale for Healthy Adult and Adolescent Volunteers Enrolled in Preventive Vaccine Clinical Trials](#FDA2023) [15]  Abbreviations: mm, millimetres |
| --- | --- | --- | --- | --- | --- | --- | --- | --- | --- | --- | --- | --- | --- | --- | --- | --- | --- | --- | --- | --- | --- | --- | --- | --- | --- | --- | --- | --- | --- | --- | --- | --- | --- | --- | --- | --- | --- | --- | --- | --- | --- | --- | --- | --- | --- | --- | --- | --- |

**Influenza vaccine compositions**

Each 0.5 mL of **FlucelVax Quad** influenza vaccines contained 15 μg of the influenza virus haemagglutinin from each of the four types of influenza virus (60 μg total) as recommended by the Australian Influenza Vaccine Committee for the 2024 Southern Hemisphere winter:

• A/Wisconsin/67/2022 (H1N1)pdm09-like virus (A/Georgia/12/2022 CVR-167)

• A/Massachusetts/18/2022 (H3N2)-like virus (A/Sydney/1304/2022)

• B/Austria/1359417/2021-like virus (B/Singapore/WUH4618/2021)

• B/Phuket/3073/2013-like virus (B/Singapore/INFTT-16-0610/2016)

Each dose of **Fluad Quad** contained 15 μg of the influenza virus haemagglutinin from each of the four types of influenza virus (60 μg total) as recommended by the Australian Influenza Vaccine Committee for the 2024 Southern Hemisphere winter and also contained the MF59C.1 adjuvant (9.75 mg squalene, 1.175 mg of polysorbate 80, 1.175 mg of sorbitan trioleate, 0.66 mg of sodium citrate dihydrate and 0.04 mg of citric acid monohydrate) at pH 6.9-7.7.

• A/Victoria/4897/2022 (H1N1)pdm09-like virus (A/Victoria/4897/2022 IVR-238)

• A/Thailand/8/2022 (H3N2)-like virus (A/Thailand/8/2022 IVR-237)

• B/Austria/1359417/2021-like virus (B/Austria/1359417/2021 BVR-26)

• B/Phuket/3073/2013-like virus (B/Phuket/3073/2013 BVR-1B)

**Immunology assays**

MN-CPE assay

The micro-neutralisation (MN) assay was performed as described. Briefly, serial 2-fold dilutions of human serum samples, starting from 1:10 to 1: 5120, were incubated with an equal volume of SARS-CoV-2 viral solution containing 25 tissue culture infective dose 50% (TCID_50_) for 1 h at room temperature. After incubation, 100 µl of the serum–virus mixture was transferred to a 96-well plate containing an 80% sub-confluent Vero E6 cell monolayer. The plates were incubated at 37℃ and 5% CO_2_. At the end of incubation, the presence/absence of a cytopathic effect (CPE) was assessed by means of an inverted optical microscope. A CPE higher than 50% was indicative of infection. The MN titre was expressed as the reciprocal of the highest serum dilution showing protection from viral infection and CPE. A titre of 10 was considered as the lower limit of quantitation (LLOQ) and a titre equal to 5 was considered as negative. All experiments with live SARS-CoV-2 viruses were performed inside the Biosecurity Level 3 laboratories of VisMederi Srl.

Haemagglutinin inhibition assay:

All serum samples were pre-treated with receptor destroying enzyme (RDE) (ratio 1:5) from Vibrio Cholerae (Sigma Aldrich, St. Louis, MO, USA) for 18 h at 37ºC in a water bath and then heat inactivated for 1 h at 56ºC in a water bath with 8% sodium citrate (ratio 1:4). Turkey red blood cells (TRBCs) were centrifuged two times, washed with 0.9% saline solution, and adjusted to a final dilution of 0.35%.

From an initial dilution of 1:10, serum samples were 2-fold diluted in duplicate with 0.9% saline solution in a 96-well plate. Twenty-five μL of standardized viral antigen was added to each well and the mixture was incubated at room temperature for one hour. TRBCs were added and, after one hour of incubation at room temperature, the plates were evaluated for the presence of agglutination inhibition. The antibody titrei s expressed as the reciprocal of the highest serum dilution showing complete inhibition of agglutination. Since the starting dilution was 1:10, the lower limit of detection (LoD) for the antibody titre was 10. When the titre was under the detectable threshold, the results were conventionally expressed as 5 (half the lowest detection threshold) for calculation of geometric mean titres.

**References**

Manenti A, Maggetti M, Casa E, Martinuzzi D, Torelli A, Trombetta CM, et al. Evaluation of SARS-CoV-2 neutralizing antibodies using a CPE-based colorimetric live virus micro-neutralization assay in human serum samples. J Med Virol. (2020) 92:2096–104.

Manenti A, Molesti E, Maggetti M, Torelli A, Lapini G, Montomoli E. The theory and practice of the viral dose in neutralization assay: Insights on SARS-CoV-2 “doublethink” effect. J Virol Methods. (2021) 297:114261.

Marchi S, Manini I, Kistner O, Piu P, Remarque EJ, Manenti A, Biuso F, Carli T, Lazzeri G, Montomoli E, Trombetta CM. Serologically-based evaluation of cross-protection antibody responses among different A(H1N1) influenza strains. Vaccines (Basel). 2020 Nov 5; 8(4):656.

**Sample size justification**

A total sample size of approximately 1680 participants (1200 participants 18 to 64 years of age, and 480 participants ≥65 years of age), and a sample size of 385 participants of study ARCT-154-J01 (PPS-1) is proposed for the following study objectives:

1) the coprimary objective of simple superiority of the ARCT-2303 vaccine booster (groups 2a and 2b) compared to the ARCT-154 booster for the Omicron XBB.1.5 subvariant

Superiority of GMT_ARCT-2303 (booster)_ to GMT_ARCT-154 (booster)_

The superiority margin for the Omicron XBB.1.5 subvariant is defined as 1.0 for the GMTs ratio, thus the lower limit of the 2sided 95% CI for GMT ratios (ARCT-2303/ARCT-154) is higher than 1.0.

Assumptions for the above calculations for objective 1) are:

The GMT ratio between ARCT-2303 (Day 29) and ARCT-154 (booster; ARCT-154-J01 study) (Day 29) groups is expected to be 1.5, and the common standard deviation is expected to be 0.60 in the log_10_ scale.

2) the coprimary noninferiority of the ARCT-2303 vaccine booster (groups 2a and 2b) compared to the ARCT-154 booster for the Omicron XBB.1.5 subvariant

Noninferiority (NI) of SCR_ARCT-2303 (booster)_ minus SCR_ARCT-154_

Assumptions for the above calculations for objective 2 are:

The difference between SCR in ARCT-2303 (Day 29) and SCR in ARCT-154 (booster; ARCT-154-J01 study) (Day 29) groups is expected to be >10% and the SCR is equal to 50% in the ARCT-154 group.

The noninferiority margin for the Omicron XBB.1.5 subvariant is defined as 5% for the SCRs difference, thus the lower limit of the 2sided 95% CI for SCR difference (ARCT-2303 minus ARCT-154) is higher than - 5% (minus 5%).

A sample size of up to 385 participants of study ARCT-154-J01, who received ARCT-154 vaccine booster, provided evaluable pre- and post-vaccination blood samples, and did not have SARS-CoV-2 infection and protocol deviations that impact on immunogenicity assessment (PPS-1), and up to 560 participants in the ARCT-2303 (groups 2a and 2b) provides approximately 99% power (n=350 and n=500 in the two groups if there is drop out of approximately 10%) to demonstrate each of the 2 coprimary objectives; therefore, there is at least 97% overall power to demonstrate the two coprimary objectives, 1 and 2.

If coprimary objectives 1 and 2 are met, then the second step was to be assessing the noninferiority of immune response after co-administration and standalone administration of ARCT-2303 and Flucelvax Quadrivalent vaccines.

The noninferiority objectives for the co-administration are:

3) vaccination with Flucelvax Quadrivalent, when given concomitantly with ARCT-2303, compared to that of Flucelvax Quadrivalent, when given standalone

NI of GMT _QIV+ ARCT-2303_ to GMT _QIV + Placebo_ (group 1a vs group 3a)

4) vaccination with ARCT-2303, when given concomitantly with Flucelvax Quadrivalent, compared to that of ARCT-2303, when given standalone

NI of GMT _ARCT-2303 + QIV_ to GMT _ARCT-2303+placebo_ (group 1a vs group 2a)

The noninferiority margin is defined as 0.67 for the GMTs ratios.

The assumptions for the above calculations are:

the GMT ratios between the

a-Flucelvax Quadrivalent, when given concomitantly with ARCT-2303, compared to Flucelvax Quadrivalent, when given standalone,

b- ARCT-2303, when given concomitantly with Flucelvax Quadrivalent, compared to ARCT2303, when given standalone,

is expected to be 1.0 and the common standard deviation is expected to be 0.60 in the log_10_ scale.

A sample size of 400 participants per group (groups 1a, 2a, and 3a) provides 97.5% power (n=360 per group if there is drop out is approximately 10%) to demonstrate noninferiority for each one of the 5 strains (4 strains for influenza vaccine and 1 strain for ARCT-2303); therefore, there is at least 88% overall power to demonstrate all of the noninferiority objectives. The overall power to demonstrate all 4 coprimary objectives is at least 85%.

| **Supplementary table 3.**  Geometric meant titers (GMT), geometric mean fold rises (GMFR) and seroconversion rates (SCR) of neutralizing antibodies against the Omicron XBB.1.5.6 sub-lineage 28 days after vaccination with ARCT-2303 by gender. | | |
| --- | --- | --- |
| **Group -** | | **2a and 2b** |
| **Vaccines -** | | **ARCT-2303 + Placebo** |
| **Males** | **n =** | **166** |
|  | **Day 1 GMT^a^** (95% CI) | **140** (114-173) |
|  | **Day 29 GMT^a^** (95% CI) | **1000** (846–1182) |
|  | **GMFR^a^** (95% CI) | **7.13** (5.98–8.51) |
|  | **SCR^a^**  n (**%**) | 124 (**74.7**) |
| **Females** | **N =** | **307** |
|  | **Day 1 GMT^a^** (95% CI) | **158** (136-182) |
|  | **Day 29 GMT^a^** (95% CI) | **1214** (1068–1379) |
|  | **GMFR^a^** (95% CI) | **7.70** (6.81–8.70) |
|  | **SCR^a^**  n (**%**) | 225 (**74.9**) |
| a:  From unadjusted calculations. | | |

| **Supplementary table 4.**  Geometric meant titers (GMT), geometric mean fold rises (GMFR) and seroconversion rates (SCR) of neutralizing antibodies against the Omicron XBB.1.5.6 sub-lineage 28 days after vaccination with ARCT-2303 by country. | | |
| --- | --- | --- |
| **Group -** | | **2a and 2b** |
| **Vaccines -** | | **ARCT-2303 + Placebo** |
| **Australia** | **n =** | **207** |
|  | **Day 1 GMT^a^** (95% CI) | **130** (107-157) |
|  | **Day 29 GMT^a^** (95% CI) | **835** (717–972) |
|  | **GMFR^a^** (95% CI) | **6.44** (5.56–7.46) |
|  | **SCR^a^** n (**%**) | 138 (**66.7**) |
| **Philippines** | **n =** | 203 |
|  | **Day 1 GMT^a^** (95% CI) | 157 (133-186) |
|  | **Day 29 GMT^a^** (95% CI) | **1394** (1203–1615) |
|  | **GMFR^a^** (95% CI) | **8.86** (7.53–10.43) |
|  | **SCR^a^** n (**%**) | 161 (**79.3**) |
| **Honduras** | **n =** | 27 |
|  | **Day 1 GMT^a^** (95% CI) | **340** (206-561) |
|  | **Day 29 GMT^a^** (95% CI) | **2440** (1664–3577) |
|  | **GMFR^a^** (95% CI) | **7.17** (4.61–11.16) |
|  | **SCR^a^** n (**%**) | 22 (**81.5**) |
| **Costa Rica** | **n =** | 36 |
|  | **Day 1 GMT^a^** (95% CI) | **161** (105-248) |
|  | **Day 29 GMT^a^** (95% CI) | **1163** (792–1707) |
|  | **GMFR^a^** (95% CI) | **7.21** (5.50–9.45) |
|  | **SCR^a^** n (**%**) | 28 (**77.8**) |
| a:  From unadjusted calculations. | | |

**Supplementary figure 2.**

Geometric fold Increase of neutralizing antibodies on Days 29 and 181 versus Day 1 for randomly selected study participants who received ARCT-2303 vaccine on Day 1.


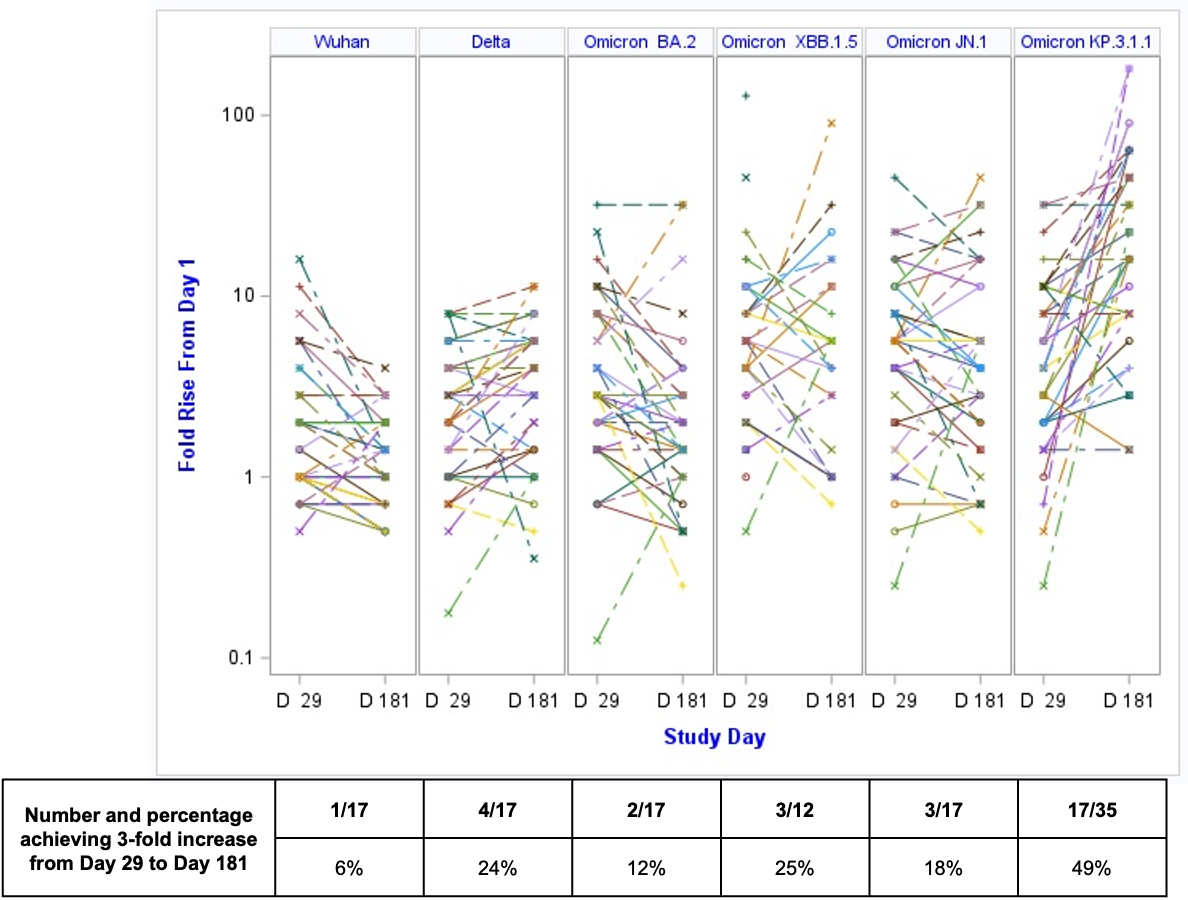

Supplement: Manuscript_clean version_24 July 2025.docx (pg no: 1–12) [file mmc3.docx]
